# Genetic and sociodemographic factors associated with trajectories of physical and mental health multimorbidity in a South Asian cohort in the UK: A multistate modelling analysis

**DOI:** 10.1371/journal.pmed.1004844

**Published:** 2026-07-09

**Authors:** Daniel Stow, Ruby S. M. Tsang, Ioanna K. Katzourou, Qinqin Huang, Miriam Samuel, Megan L. Wood, Jack F. G. Underwood, Genes and Health Research Team, The LIfespaN multimorbidity research Collaborative (LINC), Rupert A. Payne, James T. R. Walters, Nicholas J. Timpson, Inês Barroso, Hilary C. Martin, Peter A. Holmans, Marianne B. M. van den Bree, Rohini Mathur, Sarah Finer

**Affiliations:** 1 Wolfson Institute of Population Health, Queen Mary University of London, London, United Kingdom; 2 Population Health Sciences, Bristol Medical School, University of Bristol, Bristol, United Kingdom; 3 MRC Integrative Epidemiology Unit, University of Bristol, Bristol, United Kingdom; 4 Centre for Neuropsychiatric Genetics and Genomics, Cardiff University, Cardiff, United Kingdom; 5 Wellcome Sanger Institute, Cambridge, United Kingdom; 6 School of Psychology, University of Leeds, Leeds, United Kingdom; 7 Neuroscience and Mental Health Innovation Institute, Division of Psychological Medicine and Clinical Neurosciences, Cardiff University, Cardiff, United Kingdom; 8 Medical School, University of Exeter, Exeter, United Kingdom; 9 Exeter Centre of Excellence for Diabetes Research (EXCEED), University of Exeter Medical School, University of Exeter, Exeter, United Kingdom; Rigshospitalet, DENMARK

## Abstract

**Background:**

UK South Asian populations are at high risk of physical and mental health multimorbidity, which means they live with multiple long-term conditions. The life course emergence of multimorbidity, its underlying aetiology, and consequences for future health and mortality have yet to be studied in this population.

**Methods and findings:**

We studied Internalising (depression, anxiety, somatoform disorders) and Cardiometabolic (hypertension, obesity, type 2 diabetes, chronic kidney disease, dyslipidaemia) MultiMorbidity (ICM-MM): the lifetime occurrence of ≥1 internalising mental health condition AND ≥1 cardiometabolic condition in a longitudinal cohort of Genes and Health study participants with linked genetic and health data from 1st April 1997–24th November 2024. We used multi-state models to investigate trajectories in ICM-MM and risk of major cardiovascular or renal events (CVR) or non-CVR death. We used flexible parametric models to estimate baseline hazards for health state transitions, adjusting for sociodemographic factors, a polygenic risk score (PRS) for ICM-MM (ICM-MM_PRS_), and describe 10-year simulated health state probabilities. Over 10.2 years median follow-up of 23,554 British Bangladeshi and Pakistani participants (median baseline age 31.1 years, 12,934 [54.9%] women), 3,159 (13.4%) developed ICM-MM; 1,522 (6.5%) CVR; and there were 103 (0.4%) non-CVR deaths. Women were less likely to remain healthy, with higher probability of developing internalising conditions and subsequent ICM-MM, but lower risk of CVR than men. Younger age was associated with higher risk of developing internalising conditions. Bangladeshi ethnicity, higher deprivation, and smoking were all associated with higher probability of ICM-MM. 10-year CVR risk was highest for people who developed ICM-MM via the trajectory cardiometabolic-to-internalising (versus internalising-to-cardiometabolic) in mid-life (age 40). Higher ICM-MM_PRS_ was associated with higher probability of ICM-MM via cardiometabolic conditions rather than internalising conditions. Our findings are based on routinely collected electronic health records from East London, which incompletely capture individual-level and time-varying risk factors, remission or recovery, and may not reflect British Bangladeshi and British Pakistani communities across the UK. The PRS was derived largely from GWAS of European ancestry populations, which may limit its transferability to this cohort.

**Conclusions:**

The burden of multimorbidity is high in British Bangladeshi and British Pakistani populations. Young Bangladeshi women are at high risk of ICM-MM, while men are at higher risk of CVR. Detection and intervention strategies for physical and mental health multimorbidity should be targeted early in the lifecourse, for those at highest risk.

## Author summary

**Why was this study done?:**

- Multimorbidity is defined as the co-occurrence or lifetime occurrence of two or more long-term health conditions. South Asian populations living in the UK are at high risk of multimorbidity, which has a major impact on their health and quality of life.
- A common and serious type of multimorbidity, involves the co-occurrence of internalising mental health conditions (depression, anxiety, somatoform disorders) and cardiometabolic conditions. Little is known about the order in which these conditions develop, or what drives this in South Asian populations.
- Understanding the role of social demographic, and genetic factors in how these health trajectories develop could help identify those at greatest risk and inform intervention strategies.

**What did the researchers do and find?:**

- We followed over 23,000 British Bangladeshi and Pakistani volunteers in the Genes & Health study, using secure, anonymised health and linked genetic data to track the development of internalising and cardiometabolic multimorbidity over 10 years.
- Women, younger people, those living in more deprived areas, and those of Bangladeshi ethnicity were most likely to develop this type of multimorbidity, and men were at higher risk of major cardiovascular or renal events.
- People who developed a cardiometabolic condition before an internalising health condition had a slightly higher risk of major cardiovascular or renal events than those whose mental health condition came first.
- A polygenic risk score for multimorbidity was associated with a higher likelihood of following this higher-risk trajectory.

**What do these findings mean?:**

- Bangladeshi women may benefit from earlier routine health screening given the high probability of developing multimorbidity before the age at which screening is currently offered in England.
- Polygenic risk scores show modest potential as an addition to future precision health approaches for identifying people at high risk of multimorbidity.
- These findings are based on routinely collected electronic health records from East London, which do not capture individual-level risk factors such as diet and exercise and may not fully represent British Bangladeshi and Pakistani communities across the UK.

## Abstract 3

In a multistate modelling analysis, Daniel Stow and colleagues investigate genetic and sociodemographic factors associated with trajectories of physical and psychological multimorbidity in a South Asian cohort in the UK.

## Introduction

Over a third of the world’s population live with multimorbidity [1], often referred to as multiple long-term conditions, and defined as the co-occurrence [2] or lifetime occurrence [3] of two or more long-term health conditions. Multimorbidity has a profound impact on the lives of patients and their families, and has led to increasing demands on health and social care systems [4]. Multimorbidity disproportionately affects those living in deprived circumstances [5], and the prevalence of multimorbidity is projected to rise as populations age [6], further increasing existing health inequalities.

Population-based studies in the United Kingdom (UK) have identified patterns of frequently co-occurring long-term conditions (sometimes referred to as multimorbidity clusters), highlighting some of the most prevalent combinations, including hypertension, type 2 diabetes, dyslipidaemia, obesity, depression, and anxiety [7]. UK-based studies have also highlighted the impact of multimorbidity on health service utilisation and associated costs (including increased frequency of primary and secondary healthcare contacts, and number of prescribed medications) [8,9]. However, despite its major impact at individual and population level and on health services, multimorbidity remains under-studied. There is an urgent need to study how and why multimorbidity develops so that effective prevention and intervention strategies can be evaluated and implemented [3,4]. Some research is starting to address these knowledge gaps by examining longitudinal ordering of long-term conditions or ‘trajectories’ leading to multimorbidity.

Trajectory-based approaches can offer insights as to the most common or harmful trajectories, as well as identifying index conditions to prioritise for intervention to prevent multimorbidity. Existing studies have identified the trajectories most strongly associated with reduced life-expectancy (in a physical-mental health multimorbidity cluster comprising diabetes, psychosis, and congestive heart failure) [10], and highlighted associations between sociodemographic factors and longitudinal accrual of long-term conditions leading to multimorbidity [11,12]. However, this prior research does not elucidate aetiological mechanisms underlying the development of multimorbidity trajectories and these studies use broad ethnicity categories (e.g., South Asian) that are confounded by socio-economic position and do not reflect the social, behavioural and ancestral differences within them.

In the UK, South Asian populations are at the highest risk of early-onset multimorbidity, leading to greater health burden, and reduced life expectancy [13]. Pakistani and Bangladeshi populations specifically are also more likely than other South Asian (and European populations) to live with multiple long-term conditions and report poorer health [14,15]. The early emergence of multimorbidity in these populations, is supported by epidemiological and genomic literature demonstrating high genetic risk, and early onset of conditions such as type 2 diabetes [16], which are a common component of most multimorbidity clusters. There is emerging literature suggesting that commonly co-occurring conditions such as depression and type 2 diabetes may share some common aetiology through immunological and genetic pathways [17,18]. However, whilst the genetic basis of single common conditions is well-characterised and leading to combined genetic and clinical risk models being implemented within health systems such as the National Health Service (NHS) [19,20], there is very little understanding of the shared genetic risk of multimorbidity, and how this manifests across the lifecourse. Without such knowledge, the prediction and prevention of multimorbidity may be limited to existing clinical risk tools (particularly for single conditions), which may not offer effective screening for multimorbidity risk.

Many operationalisations of multimorbidity have been used in existing research [2,4]. Here, we use a collaborative-specific operationalisation: the lifetime co-occurrence of internalising (depression, anxiety, somatoform disorders) and cardiometabolic conditions (hypertension, obesity, type 2 diabetes, chronic kidney disease, dyslipidaemia) to characterise physical-mental health multimorbidity health trajectories in a large UK sample of British Bangladeshi and Pakistani individuals and investigate whether a multimorbidity polygenic risk tool alongside traditional risk factors would identify those at risk. We selected this population due to the greater burden and earlier onset of several of the single conditions in this multimorbidity cluster, and to redress the lack of representation of South Asian populations in genomic research [21].

In this study, we focus on a cluster of physical and mental health multimorbidity comprising internalising (as opposed to externalising) [22] mental-health conditions and cardiometabolic conditions: one of the most common types of physical and mental health multimorbidity in older age [23]. This constellation of conditions, or similar, are commonly observed in clustering studies [1,24], and account for a significant cost to health systems [25], and yet there is a lack of evidence on how to prevent, or manage them effectively in multimorbidity care models [26,27]. In this study, we used multi-source electronic health records to describe the transitions from a healthy state to the development of either an internalising or cardiometabolic condition and subsequent multimorbidity, followed by a major cardiovascular or renal event and death. We then investigated the influence of ethnicity (Bangladeshi and Pakistani), sociodemographic and genetic risk, using a novel polygenic risk score for multimorbidity, on progression through these trajectories.

## Methods

### Patient and public involvement

Nine members of the public with lived experiences of multiple long-term conditions worked with the Lifespan Multimorbidity Research Collaborative (LINC), including Jane Sprackman and Shahid Khan who were also Patient and Public Involvement (PPI) co-applicants on LINC and represented the wider PPI group at study management meetings. PPI partners worked in collaboration with primary and secondary care doctors, and other members of the research team to define the study-specific multimorbidity operationalisation. They also contributed to the design of this analysis, highlighting priority areas and informing the research questions, including the focus on the impact of condition ordering on future health events related to multimorbidity.

### Setting

Genes & Health (G&H) is an ongoing, population-based study that recruits participants of self-reported British Pakistani and British Bangladeshi ethnicity aged 16 and over from East London, Manchester, Birmingham, and Bradford in the United Kingdom [28]. Volunteers are recruited by bilingual health researchers from community (including mosques, markets and libraries), and healthcare settings (including primary care surgeries and outpatient clinics). We restricted this study to participants with primary care data, meaning only individuals in East London were in the analysis sample (see Data sources). The convenience sample is broadly representative of the background population in East London—with some over-representation of women, and those aged <45 years [28]. The majority of G&H volunteers in East London live in areas characterised by high levels of deprivation Table A in S3 Text.

### Data sources

At recruitment, participants complete a brief demographic questionnaire, consent to secure linkage to their NHS electronic health records, and provide a saliva sample for DNA studies. Genes & Health hosts data for researchers in a secure ISO27001-certified Trusted Research Environment. The Trusted Research Environment is currently hosted by Google Cloud Platform in London. The Data Controller is Queen Mary University of London. Health phenotypes in G&H are captured from primary care providers across the inner north-east London boroughs of City and Hackney, Newham, Tower Hamlets and Waltham Forest) [29]. Linkage to NHS Digital datasets provides national-level coverage of healthcare data from Hospital Episode Statistics (HES) Admitted Patient Care (APC) records for events occurring in hospitals in England and Wales. We accessed information on underlying cause and date of death from linked Office of National Statistics (ONS) Civil Registrations data (CRD). All health phenotypes were defined using existing SNOMED (primary care data) and ICD-10 (HES-APC) publicly available codelist resources [30]. Saliva sample genotyping was performed with the Illumina Infinium Global Screening Array V3 Chip and imputation via TOPMed servers [31] using the r3 reference panel [32].

### Ethics

Genes & Health was approved by the National Research Ethics Committee—London and Southeast (reference 13/LO/124). Written informed consent allowing the collection, analysis, and publication of results from health and genetic data is obtained from all study volunteers.

### Cohort design

We generated a longitudinal cohort nested in G&H using routine electronic health record linkages, and following a prospective analysis plan developed in March 2023. Cohort entry was defined as the most recent of (i) registration with a primary care practice contributing primary care data to G&H, (ii) turning 16 years of age (minimum eligible age of G&H volunteers), or (iii) HES-APC linkage initiation date (1st April 1997 per Herbet and colleagues, 2017) [33]. Start of follow-up was initially defined as the cohort entry date, but was revised to cohort entry date plus a six-month lag (or ‘washout’) period, to exclude prevalent or pre-existing conditions from the incidence risk set following peer review in December 2025 [34,35]. The end of follow-up was defined as the earliest of (i) loss to follow-up (12 months after the most recent primary care or HES-APC record) [36], (ii) date of death, from ONS-CRD, (iii) administrative censoring (date of data refresh—set to November 2024), or (iv) de-registration from a primary care practice contributing data to G&H.

### Participants

Eligible participants were age ≥16 years old, with ≥1 record in G&H primary care data, no record of any internalising or cardiometabolic conditions before start of follow-up, and total follow-up time >0 days.

### Multimorbidity definition

This work is part of the LIfespaN multimorbidity research Collaborative (LINC) [37]. LINC defines Internalising and CardioMetabolic MultiMorbidity (ICM-MM) as the lifetime occurrence of ≥1 internalising mental health condition (depression, anxiety, somatoform disorders) AND ≥one cardiometabolic condition (hypertension, type 2 diabetes, obesity, dyslipidaemia, chronic kidney disease). All of these conditions are commonly diagnosed and managed in primary care settings in the UK—for further information on the LINC multimorbidity definition please see S1 Text. Age of onset for each condition was defined as the first occurrence of any relevant code in primary care or HES records.

### Outcome measures

The primary endpoint for the multi-state model employed in this study was a major cardiovascular [38] or renal event (CVR). These are among the leading causes of death globally, and a common consequence of the cardiometabolic conditions in our ICM-MM definition [39]. CVR was defined as the earliest occurrence in the electronic healthcare record data of, or where the underlying cause of death in ONS mortality data [40] was due to: atrial fibrillation and flutter, heart failure, coronary heart disease, peripheral vascular disease, cerebrovascular disease (transient ischaemic attack/stroke), and end-stage renal disease. All other underlying causes of death were grouped together as a competing risk state: ‘non-CVR death’ in the multi-state model.

### Exposure measures

#### Demographic variables.

Participants’ self-reported gender, and ethnicity (Bangladeshi or Pakistani) were taken from the G&H demographic questionnaire. Participants responding ‘other’ to the ethnicity item were excluded from this analysis. Date of birth was taken from participants’ primary care electronic records. We used Index of Multiple Deprivation (IMD 2019) [41] quintiles from primary care records, collapsing the top three quintiles (1 = most deprived… 3+ = least deprived) due to the very small number of participants in our background population and sample living in the least deprived areas, and to aid model convergence. Smoking status categories were defined as ‘ever’ or ‘never’ at study baseline using an existing codelist [42].

#### Genetic risk.

PRSs measure an individual’s genetic predisposition to a given trait. They are calculated by combining the effects of many genetic variants (called single-nucleotide polymorphisms, SNPs), with effect sizes drawn from large-scale genome-wide association studies (GWAS) [43].

In this study, we used a novel PRS developed for ICM-MM called ‘ICM-MM_PRS_’, which we have described in full previously [44]. Briefly, the score was constructed in three steps: Firstly, we identified GWAS studies for each of the individual ICM-MM conditions, selecting those that did not include the UK Biobank study [45], or made available summary statistics excluding UK Biobank participants. Secondly, we used a polygenic prediction method called ‘PRS-CS’ [46] to create separate PRSs for each condition (referred to as ‘PRS_TRAIT_’) using UK Biobank data. Finally, we used elastic net regression, implemented in the R package ‘PRSmix’ [47], to find the optimal combination and weighting of the seven PRS_TRAIT_ scores for predicting ICM-MM in UK Biobank participants. The model was adjusted for self-reported gender and 10 genetic principal components to account for population stratification.

The final ICM-MM_PRS_ retained PRS_TRAIT_ for depression, pulse pressure, type 2 diabetes, BMI and LDL-cholesterol (listed in the order of their weighting, from highest to lowest). For the present analysis, we calculated the retained PRS_TRAIT_ for individuals in G&H using PLINK 2.0 (a free, open-source genomic analysis toolset) [48] and then combined these into ICM-MM_PRS_ using the PRSMix weights from UK Biobank. To account for population stratification in G&H we regressed out the first 20 genetic principal components. We then standardised ICM-MM_PRS_ (z-transformation) to make scores easier to interpret in our multi-state models.

### Statistical analysis

**Fig 1:**
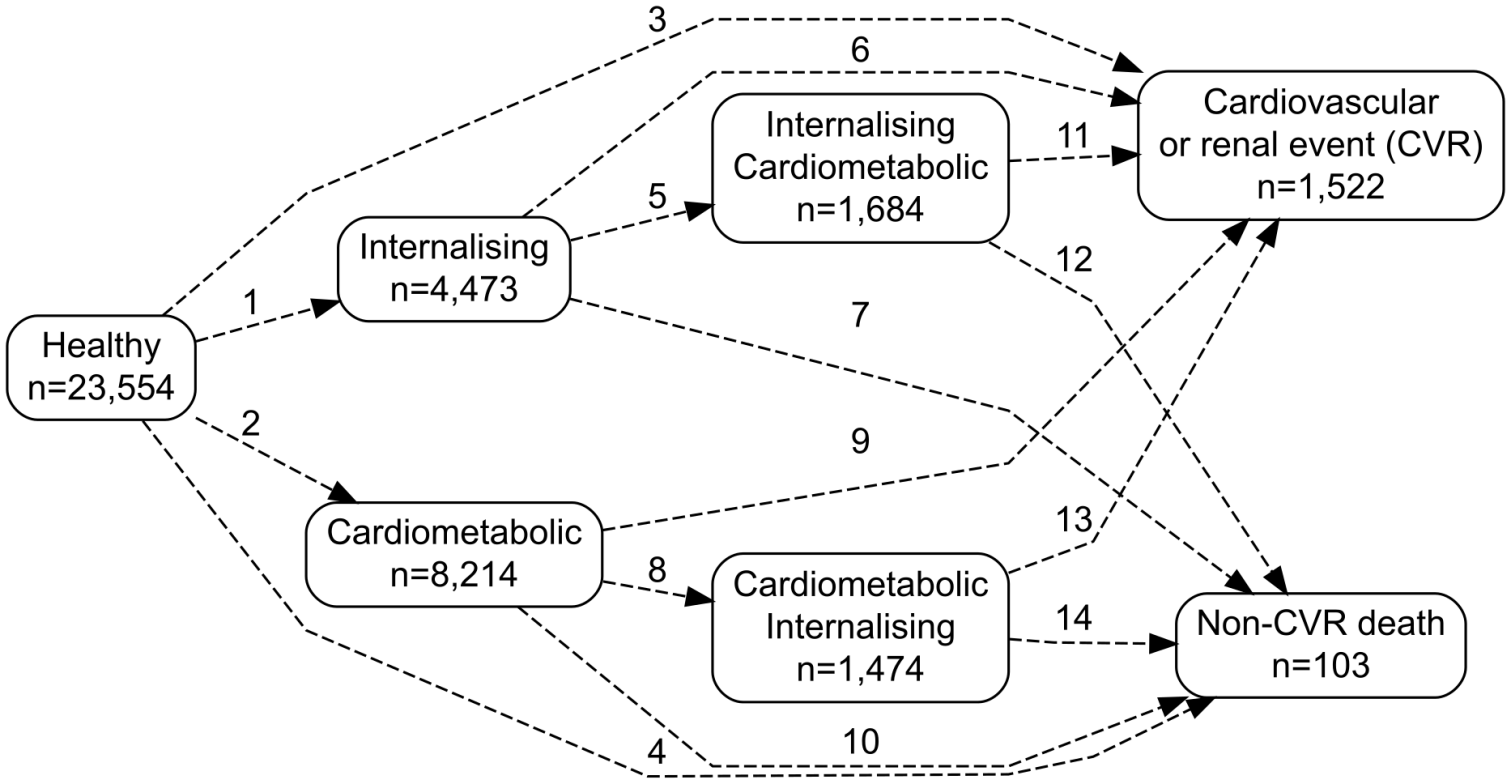
The seven-state, 14 transition multi-state model used to define health transitions. Internalising conditions are anxiety, depression, and somatoform disorders; cardiometabolic conditions are hypertension, type 2 diabetes, obesity, dyslipidaemia, chronic kidney disease. Major cardiovascular or renal event (CVR) is coded occurrence of, or death due to: atrial fibrillation and flutter, heart failure, coronary heart disease, peripheral vascular disease, cerebrovascular disease, and end-stage renal disease. Non-CVR death is all other (non-CVR) cause mortality.

We used a semi-Markov (‘clock-reset’) multi-state model [49] to describe 14 transitions between seven health states (Fig 1), starting from ‘otherwise healthy’ or free from any ICM-MM conditions at study baseline. The multi-state model was made using the ‘fmsm’ command in the flexsurv package [50]. Transitions were allowed from ‘healthy’ to single disease states (any internalising OR any cardiometabolic condition) and then to ICM-MM, accounting for disease ordering (i.e., internalising → cardiometabolic versus cardiometabolic → internalising), and then to CVR or non-CVR death from either ICM-MM trajectory. Direct transitions to CVR and non-CVR death were allowed from any other health state in the model (e.g., otherwise healthy → death, internalising → CVR), but transitions from CVR or non-CVR death to other health states were not (i.e., these were ‘absorbing states’). Individuals with internalising, cardiometabolic, CVR or death events recorded on the same day do not inform transitions in the multi state model, and these participants (*n* = 56) were excluded from the study.

Baseline hazards for each transition were estimated separately using restricted cubic splines as described in Royston and Parmar, 2002 [51] using ‘flexsurvspline’ from the ‘flexsurv’ package [50]. We selected the number of interior knots for each model via Akaike information criterion (AIC) and comparison of modelled versus observed survival plots for each transition (see S2 Text for details). All models were adjusted for continuous age (modelled with splines) and ICM-MM_PRS_, and categorical IMD, gender, ethnicity, and smoking status. Age was defined as the age at entry into each state separately (i.e., baseline age for the ‘otherwise healthy’ state is equal to the age at cohort entry and increases for each subsequent change in health state). Participants with missing sociodemographic covariates were dropped (i.e., a complete case analysis). We assessed the proportionality assumption via Schoenfeld residuals plotted against transformed time. Where we observed non-proportional hazards, we allowed spline parameters to vary with the relevant covariate values (i.e., time-varying effects).

#### State occupation probabilities.

Predicted probabilities of being in state_i_ at time_t_ were simulated using 10,000 individuals for 20 evenly spaced time points over a 10-year window using the sim.fmsm command in the ‘flexsurv’ package [50]. All simulations were for a pre-specified covariate patterns for men and women: a Bangladeshi participant living in IMD quintile 1 (most deprived) with ICM-MM_PRS_ *z*-score of 0 (‘average risk’), and a ‘never’ smoker age 40 at the start of follow-up, chosen due to its relevance and generalisability to the NHS Health Check which is offered from age 40 in England and Wales [52]. We also examined the impact of varying age at start of follow-up, retaining all other reference covariate patterns for comparison.

#### Condition ordering and CVR risk.

To test the impact of multimorbidity on future risk of CVR, we simulated state occupation probabilities for all transitions in the multi-state model and then contrasted single condition trajectories with the multimorbid equivalent, e.g., *P(CVR* | *cardiometabolic)* versus *P(CVR | cardiometabolic*→ *internalising)*. To test the impact of condition ordering in multimorbid trajectories on future risk of CVR, we simulated state occupation probabilities for all transitions in the multi-state model, then contrasted *P(CVR | cardiometabolic* → *internalising)* with *P(CVR | internalising* → *cardiometabolic)* over a range of ages of ICM-MM onset.

#### Sociodemographic and genetic contrasts.

To test the impact of sociodemographic factors and ICM-MM_PRS_ on state occupation probabilities, we generated contrasts between the female reference covariate pattern, and the contrast of interest (e.g., *P(state*_*i*_ *time*_*t*_ *| female) − P(state*_*i*_ *time*_*t*_ *| male)*. Confidence intervals for contrasts were generated using bootstrapping via the bootci.fmsm command in flexsurv [50], with *n* = 400 iterations.

## Results

**Fig 2:**
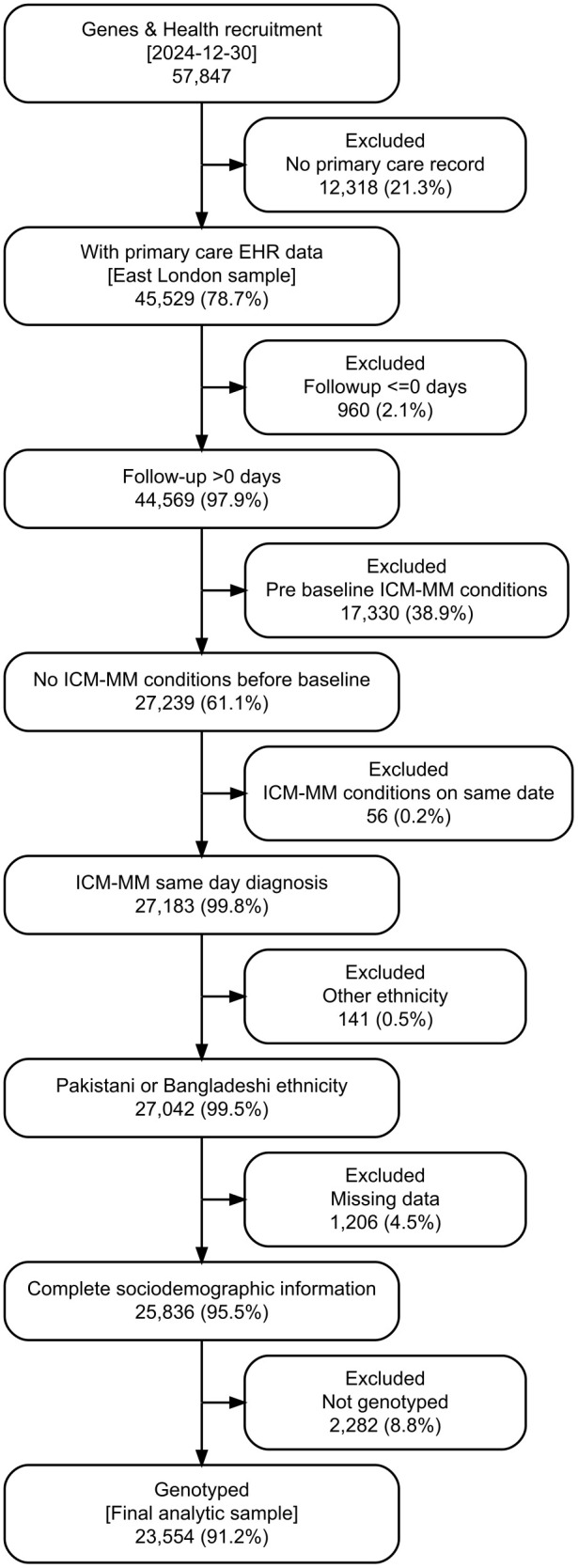
Flow chart showing eligibility of Genes & Health volunteers for this study.EHR, electronic health record; ICM-MM, internalising and cardiometabolic multimorbidity.

The flow diagram for cohort eligibility is shown in Fig 2: 23,554 G&H participants were included in the complete case analysis;12,934 (54.9%) were women, and 10,620 (45.1%) men. Baseline characteristics for the cohort are shown in Table 1, characteristics of excluded participants in Table A in S3 Text. Median age at cohort entry was 31.1 years (IQR [interquartile range]: 24.1, 38.2), median follow-up time was 10.2 years (IQR: 6.1, 16.7), and loss to follow-up (*n* = 509, 2.2%) was acceptable [53].

**Table 1:** Characteristics of participants at study baseline.

	Bangladeshi		Pakistani		Overall	
	Female	Male	Male	Female	Female	Male
	(*N* = 8,488)	(*N* = 6,963)	(*N* = 4,446)	(*N* = 3,657)	(*N* = 12,934)	(*N* = 10,620)
**Age at cohort entry**						
Mean (SD)	29.9 (9.1)	33.2 (10.7)	31.1 (10.3)	33.3 (11.8)	30.3 (9.56)	33.2 (11.1)
Median [Min, Max]	29.3 [16.5, 75.3]	33.5 [16.5, 84.5]	30.0 [16.5, 84.9]	32.7 [16.5, 82.5]	29.6 [16.5, 84.9]	33.2 [16.5, 84.5]
IMD quintile*						
1	2,886 (34.0%)	2,304 (33.1%)	656 (14.8%)	569 (15.6%)	3,542 (27.4%)	2,873 (27.1%)
2	4,410 (52.0%)	3,646 (52.4%)	2,628 (59.1%)	2,130 (58.2%)	7,038 (54.4%)	5,776 (54.4%)
3+	1,192 (14.0%)	1,013 (14.5%)	1,162 (26.1%)	958 (26.2%)	2,354 (18.2%)	1971 (18.6%)
**Smoking status**						
Never	7,435 (87.6%)	3,149 (45.2%)	4,017 (90.4%)	2,237 (61.2%)	11,452 (88.5%)	5,386 (50.7%)
Ever	1,053 (12.4%)	3,814 (54.8%)	429 (9.6%)	1,420 (38.8%)	1,482 (11.5%)	5,234 (49.3%)
**Follow up (years)**						
Mean (SD)	11.9 (7.3)	11.0 (6.9)	12.8 (7.7)	12.0 (7.5)	12.2 (7.5)	11.4 (7.2)
Median [Min, Max]	10.2 [>0.0, 27.1]	9.61 [>0.0, 27.1]	11.2 [>0.0, 27.1]	10.4 [>0.0, 27.1]	10.5 [>0.0, 27.1]	9.90 [>0.0, 27.1]

*IMD, Index of Multiple Deprivation, Lowest tertile [1] is most deprived. SD, standard deviation.

Events during follow-up are shown in Table 2. During follow up, *n* = 5,948 (25.3%), participants developed internalising conditions (median age 35.8 years, IQR: 28.7, 43.5), of which anxiety and phobia was the most common index condition (*n* = 2,620 [11.1%] – Table B in S3 Text); *n* = 9,898 (42.0%) developed cardiometabolic conditions (median age 41.7, IQR: 35.6, 48.4), of which dyslipidaemia was the most common index condition (*n* = 3,091 [13.1%] – Table B in S3 Text); and 3,159 (13.4%) developed ICM-MM (median age 42.8, IQR: 36.3, 50.2). Major cardiovascular or renal event (CVR) was experienced by *n* = 1,522 (6.5%) participants (median age 53.2, IQR:45.6, 61.3), and n *=* 103 (0.4%) participants died due to all (non-CVR) causes (median age 58.3, IQR:48.5, 70.2).

**Table 2:** Health events during study follow-up.

	Bangladeshi		Pakistani		Overall	
	Female	Male	Male	Female	Female	Male
**Internalising conditions (INT)**	2,680 (31.6%)	1,308 (18.8%)	1,353 (30.4%)	607 (16.6%)	4,033 (31.2%)	1915 (18.0%)
**Cardiometabolic conditions (CMD)**	3,585 (42.2%)	2,918 (41.9%)	1931 (43.4%)	1,464 (40.0%)	5,516 (42.6%)	4,382 (41.3%)
**ICM-MM via CMD- > INT**	636 (7.5%)	346 (5.0%)	320 (7.2%)	173 (4.7%)	956 (7.4%)	519 (4.9%)
**ICM-MM via INT- > CMD**	785 (9.2%)	336 (4.8%)	405 (9.1%)	158 (4.3%)	1,190 (9.2%)	494 (4.7%)
Cardiovascular or renal event*	307 (3.6%)	618 (8.9%)	228 (5.1%)	369 (10.1%)	535 (4.1%)	987 (9.3%)
Died**	29 (0.3%)	36 (0.5%)	20 (0.4%)	18 (0.5%)	49 (0.4%)	54 (0.5%)

*Including deaths due to cardiovascular or renal event.

**All other cause mortality (ONS civil registrations).

### Multi-state models

#### Sequence and number of events.

In total, 1,684 (28.3%) of 5,948 participants with an internalising condition went on to develop a cardiometabolic condition, median age 41.4 years (IQR 35.2, 48.2) and 1,475 (18.2%) of 9,898 people with a cardiometabolic condition went on to develop an internalising condition, median age 44.5 years (IQR 37.4, 53.2). For further details, please see Table C in S3 Text Table C.

#### State occupation probabilities.

**Fig 3:**
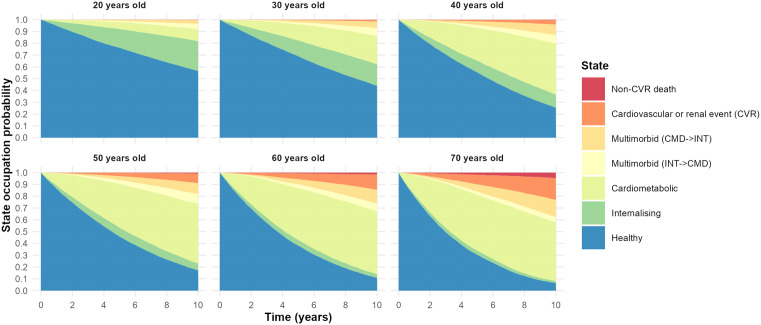
Ten-year state occupation probabilities for women across six baseline ages. Probability estimates for a Bangladeshi woman in the lowest IMD tertile, never smoker, with average genetic risk. Projections stratified across six baseline ages.

**Fig 4:**
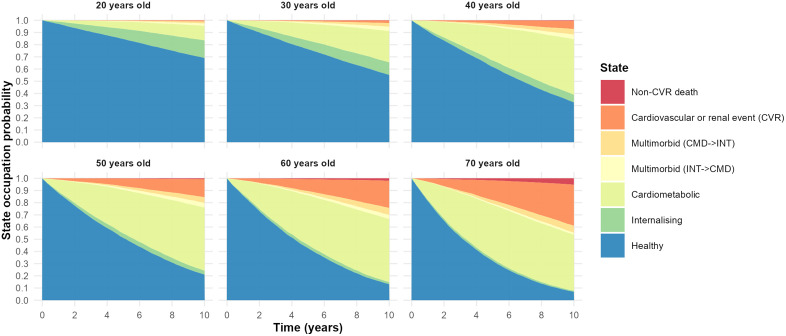
Ten-year state occupation probabilities for men across six baseline ages. Probability estimates for a Bangladeshi man in the lowest IMD tertile, never smoker, with average genetic risk. Projections stratified across six baseline ages.

Figs 3 and 4 show the state occupation probabilities for the reference covariate patterns for women (Fig 3) and men (Fig 4) across five baseline ages. These projections show how the probability of developing internalising and cardiometabolic conditions, and ICM-MM, increases over time, and with baseline age. We also observed a non-proportional hazard for baseline age in transitions to internalising states, suggesting that people were more likely to report internalising conditions at younger ages. Across all age groups, the probability of remaining healthy decreased over the 10-year period, with this decline especially pronounced in older age groups, where a high proportion of individuals were likely to experience cardiometabolic conditions. Even in younger age groups (20 and 30) the probability of experiencing multimorbidity over a 10-year time frame was between 5% and 10%. In the youngest age groups (20 and 30), multimorbid states were equally probable via cardiometabolic → internalising and internalising → cardiometabolic. From 40 onwards, multimorbidity was more likely via cardiometabolic → internalising, and the probability of experiencing death or CVR over a 10-year timeframe increased greatly after the age of 50. For details of 10-year state occupation probabilities for men and women, please see Tables D and E in S3 Text.

#### Condition ordering and CVR risk.

The 10-year probability of CVR was higher for cardiometabolic→ internalising for most baseline ages tested, and for both women and men Fig A to D in S4 Text), but the difference was only conventionally statistically significant (*p* < 0.05) for women who developed ICM-MM at the age of 40:


$$\begin{array}{cc} & P_{\text{1}}(\text{CVR }|\;internalising\;\to \;cardiometabolic)\;=\;\text{0}.\text{056},\;P_{\text{2}}(CVR\;|\;cardiometabolic\;\to \;internalising)\;\\ & \;=\;\text{0}.\text{116},\;contrast(P_{2},P_{1})=0.058\;(95\%𝐂𝐈[0.017,\;0.100]) \end{array}$$

The equivalent contrast for men age 40 was


$$\begin{array}{cc} & P_{\text{1}}(\text{CVR }|\;internalising\;\to \;cardiometabolic)\;=\;\text{0}.\text{095},\;P_{\text{2}}\;(CVR\;|\;cardiometabolic\;\to \;internalising)\;\\ & \;=\;\text{0}.\text{161},\;contrast(P_{2},P_{1})=0.078\;(95\%𝐂[-0.00,\;0.140]) \end{array}$$

#### Sociodemographic and genetic contrasts.

**Fig 5:**
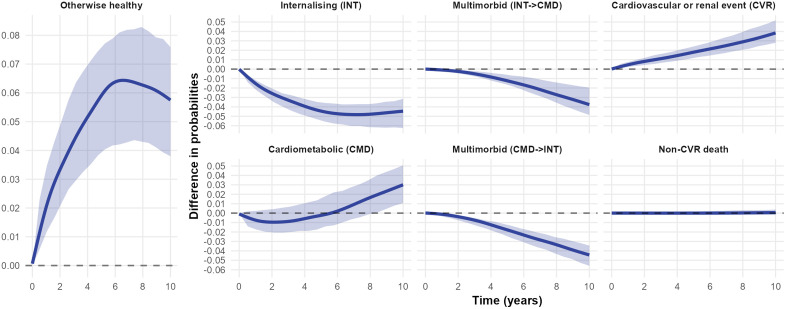
Contrast in 10-year state occupation probabilities for men vs. woman (reference). Difference in probabilities and associated 95% confidence interval of being in each health state for a Bangladeshi man age 40 in IMD tertile 1, non-smoking and with an ICM-MM-PGS of 0 vs. a Bangladeshi woman with the same covariate pattern.

For details of 10-year contrasts for all sociodemographic and genetic variables, please see Table F in S3 Text. Fig 5 shows the difference in probabilities and 95%CI for being in each health state for a man, versus the reference covariate pattern for a woman over a 10-year window and associated confidence intervals (i.e., the contrast between Figs 3 and 4). Men were more likely to remain free of ICM-MM conditions (‘otherwise healthy’), and less likely to experience internalising states (and hence ICM-MM, either via *internalising* → *cardiometabolic* or *cardiometabolic* → *internalising*). However, men had a higher probability of experiencing a CVR over the 10-year window.

**Fig 6:**
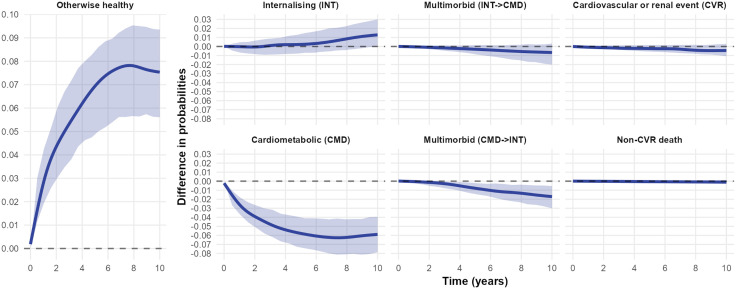
Contrast in 10-year state occupation probabilities for Pakistani vs. Bangladeshi ethnicity (reference). Difference in probabilities and associated 95% confidence interval of being in each health state for a Pakistani woman age 40 in IMD tertile 1, non-smoking and with an ICM-MM-PGS of 0 vs. a Bangladeshi woman with the same covariate pattern.

Fig 6 shows the difference in probabilities and 95%CI of health state occupation for a Pakistani woman versus the reference individual. The probability of remaining otherwise healthy (free from ICM-MM conditions) remains higher over the 10-year window for the Pakistani individual—primarily due to the reduced probability of developing a cardiometabolic condition. However, the difference in probabilities of developing ICM-MM is only slightly lower for the Pakistani individual for multimorbid states, particularly for *internalising* → *cardiometabolic*.

**Fig 7:**
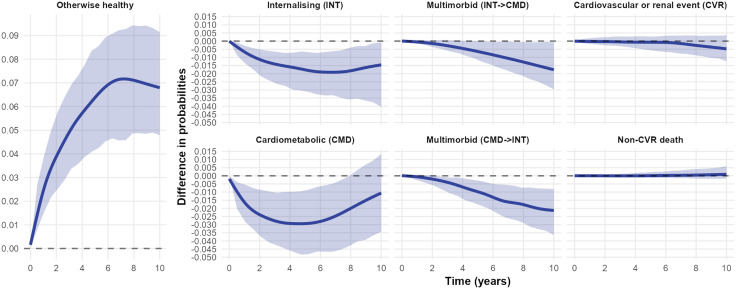
Contrast in 10-year state occupation probabilities for highest Index of Multiple Deprivation (IMD) tertile (3+) vs. IMD tertile 1 (reference). Difference in probabilities and associated 95% confidence interval of being in each health state for a Bangladeshi woman age 40 in IMD tertile 3 + , non-smoking and with an ICM-MM-PGS of 0 vs. a woman in IMD tertile 1 with the same covariate pattern.

Fig 7 shows the difference in probabilities and 95%CI, of health state occupation for someone living in the least deprived tertile (IMD 3+) versus the reference (IMD 1). The probability of remaining healthy was higher for the individual living in the least deprived tertile. Differences in overall health are driven by a reduced probability of experiencing internalising, cardiometabolic and ICM-MM health states, though there was no difference in the probability of experiencing CVR or death.

**Fig 8:**
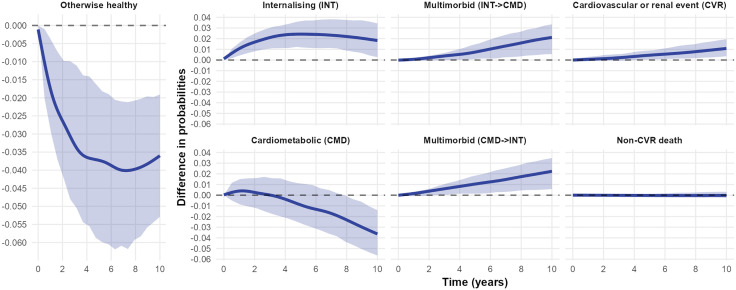
Contrast in 10-year state occupation probabilities for ‘ever’ vs. ‘never’ smoking (reference). Difference in probablities and associated 95% confidence interval of being in each health state for a Bangladeshi woman age 40 in IMD tertile 1, ever smoker and with an ICM-MM-PGS of 0 vs. a non-smoker with the same covariate pattern.

Fig 8 shows the contrast in probabilities and 95%CI for an ever versus never smoker at study baseline. The probability of an ever smoker remaining free from ICM-MM conditions was up to 3% lower over the 10-year window versus never smokers. Ever smoking was also associated with increased probability of internalising conditions as the index condition, and increased ICM-MM risk along both trajectories (*cardiometabolic* → *internalising* and *internalising* → *cardiometabolic*) following the index condition. The probability of CVR was also higher over the 10-year window.

**Fig 9:**
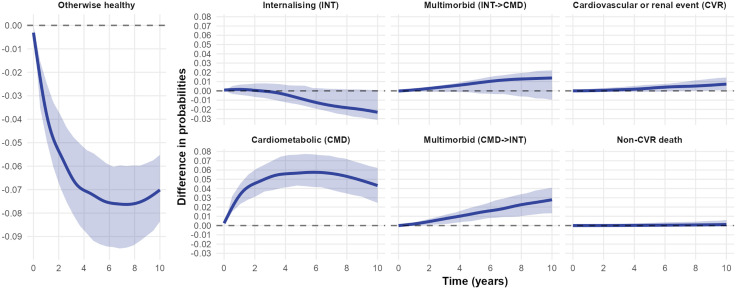
Contrast in 10-year state occupation probabilities for high genetic risk (ICM-MM_PGS_ = 2) vs. average genetic risk (ICM-MM_PGS_ = 0). Difference in probabilities and associated 95% confidence interval of being in each health state for a Bangladeshi woman age 40 in IMD tertile 1, ever smoker and with an ICM-MM-PGS of 2 vs. a an ICM-MM-PGS of 0.

Fig 9 shows the contrast in state occupation probabilities and 95%CI for someone in the top 2.5% of polygenic risk (ICM-MM_PRS_ *z*-score = 2) versus someone with average genetic risk (ICM-MM_PRS_ *z*-score = 0). Participants with a higher ICM-MM_PRS_ had a higher probability of experiencing cardiometabolic → internalising (but not internalising → cardiometabolic) trajectories and higher probability of CVR.

## Discussion

In this study, we examined internalising and cardiometabolic multimorbidity trajectories and risk of cardiovascular and renal events or death in a large cohort of British Pakistani and British Bangladeshi participants. Combining real-world healthcare data with genetic information, we examined the impact of sociodemographic and genetic factors on these multimorbidity trajectories. We found rapid accumulation of illness and multimorbidity, even in young age groups. Assuming good health at the age of 40, the probability that Bangladeshi participants living in the most deprived areas will remain free from ICM-MM conditions over the next 10 years is below 40%. For a healthy Bangladeshi woman aged 30, the probability that she will remain free of ICM-MM conditions by age 40 is below 50%. In the UK, individuals are eligible for routine screening as part of the nationwide NHS health check from age 40; our findings suggest that specific populations, such as Bangladeshi women, may benefit from earlier initiation of routine health screening with the potential for improved public health outcomes.

Methodological advances mean that multimorbidity research has progressed beyond simple counts of conditions to account for complexity and clustering, but temporality is still under-studied [54]. We found only limited evidence that condition ordering impacts later CVR risk: people who developed ICM-MM along the trajectory cardiometabolic → internalising had a slightly higher probability of developing a cardiovascular event over the next 10 years than the internalising → cardiometabolic trajectory, though the evidence for this was only conventionally statistically significant (*p* < 0.05) for women who developed ICM-MM at age 40. Our findings contribute to an established literature showing that individuals with type 2 diabetes are more likely to have a CVR if they have co-existent depression [55]. The lack of longitudinal data in these prior studies meant it was not possible to see whether these associations were driven by depression prior to, or after, the diagnosis of type 2 diabetes. By characterising the ordering of conditions, we can hypothesise why there are small (but directionally consistent) differences in progression to CVR. Individuals who develop internalising conditions after cardiometabolic disease may be experiencing a greater treatment burden from that cardiometabolic disease, leading to depression and/or anxiety, or may be non-adherent to medications that offer protection against CVR [56]. In contrast, individuals who have established internalising conditions at the time of diagnosis of a cardiometabolic disease may receive holistic care that addresses both, e.g., in the UK, screening and management of depression and anxiety is encouraged at type 2 diabetes diagnosis [57], and structured education programmes delivered to people with type 2 diabetes, typically at or soon after diagnosis, have well-characterised psychosocial benefits [58].

A key strength of our study is the availability of self-reported information on ethnicity, meaning we can highlight important health inequalities within UK South Asian populations*.* We found that Bangladeshi participants were more likely than Pakistani participants to experience ICM-MM conditions, ICM-MM and CVR, highlighting a health inequality previous large-scale registry based studies on multimorbidity trajectories in the UK would not have been able to detect due to aggregation across ethnicity groupings in the case of Chen and colleagues [12], and absence of information on ethnicity in Owen and colleagues [10] and Lyons and colleagues [11]. Ethnicity is a complex multidimensional construct that encapsulates cultural, religious and many other factors [14]. Smoking is one important potential source of variation associated with ethnicity [59] and cardiometabolic multimorbidity [60]. In our sample, Bangladeshi participants were more likely to be ‘ever’ smokers than Pakistani participants: we adjusted for smoking in our analysis to remove this potential source of confounding, but future work should investigate other environmental, sociodemographic and lifestyle factors that may be responsible for the differences we observed.

Our findings also build on existing evidence of the shared genetic architecture of many cardiometabolic and mental health traits [61,62], demonstrating that this architecture has consequences for ordering of long-term condition pairs. The polygenic risk score developed to predict ICM-MM (ICM-MM_PRS_), was associated with higher risk of ICM-MM via the trajectory cardiometabolic → internalising (the trajectory resulting in the highest probability of CVR) than for the trajectory internalising → cardiometabolic. This earlier onset cardiometabolic profile with higher internalising condition risk (and subsequent risk of cardiovascular event) should be a target for early screening and intervention. Lipid profiles are one of the current screening measures used in the NHS health check [52] and dyslipidaemia was the most common index cardiometabolic condition in our study. This builds on other recent studies in UK biobank that found lipid profiles are an important precursor to cardiometabolic multimorbidity [63].

We also found that younger otherwise healthy participants in this population were more likely to transition to internalising states (in any order) than older participants. This finding is in line with large scale studies on increasing prevalence of depression in the UK, especially in younger cohorts [64]. Our findings may have implications on future ICM-MM prevalence in this cohort (especially under the ‘lifetime occurrence’ definition of multimorbidity [3])—as younger participants continue to age. This observation may also reflect changes in behaviour and attitudes towards depression in South Asian populations [65], as well as improved ascertainment by primary care clinicians [66].

We observed similar associations between area level deprivation (IMD) and disease transitions to those found in previous multimorbidity studies [5,12,67]. Participants living in the most deprived quintile were more likely to experience internalising and cardiometabolic conditions, ICM-MM and CVR than those living in three least deprived quintiles. However, most G&H participants in our sample live in areas of East London that are in the two most deprived IMD quintiles, meaning many participants are likely to have similar exposure to environmental factors that are known to be associated with multimorbidity (air pollution in particular) [68]—but that are not well reflected in the aggregated IMD.

Our study uses routinely collected electronic healthcare records, the limitations of which are well reported (see Farmer and colleagues for an example) [69]. One key limitation is the lack of information on individual-level risk factors associated with cardiometabolic and internalising conditions. With the notable exception of smoking [70] many of these factors, such as alcohol consumption [71] and exercise [72], are poorly captured in electronic health records. We were able to adjust for smoking behaviours at study baseline, but we note that some ‘never’ smokers may have started smoking after study baseline, and ‘ever’ smokers may have stopped smoking: cessation is likely to reduce or delay onset of cardiometabolic multimorbidity [60].

We adjusted our models for deprivation using the index of multiple deprivation (IMD), which is a commonly used metric to assess area-level environmental risk exposures in England, but we note this is subject to the ecological fallacy [73]. Additionally, as IMD was ascertained at study baseline, any changes in living circumstances (and their impact on health trajectories over the course of follow-up) are not reflected in our results. Due to the very low numbers of people living in IMD quintiles four and five in this population, we collapsed these categories, further reducing the granularity of this measure. The G&H sample in East London is broadly representative of the British Bangladeshi and British Pakistani population in East London, but over-represents slightly women and people age under 45. Future work should target strategies to reweight the G&H sample to fully reflect the background population in terms of sociodemographic structure, and health [74].

In common with other studies that have used electronic healthcare records to define health phenotypes we have made a series of assumptions about ascertainment and chronicity of the conditions, as there is often no triggering contact that would make a clinician record remission/recovery. This has implications for our multi-state framework as individuals could not re-enter a previous health state, which may not reflect the episodic nature of the internalising mental health conditions. Using diagnosed obesity as part of the cardiometabolic condition definition also removed our ability to investigate BMI as a potential moderator on the proposed causal relationship between depression and diabetes [75].

One limitation of our novel ICM-MM_PRS_ is the lack of cross-ancestry GWAS available to create the contributing PRS, which are drawn from predominantly white European ancestry populations. Previous work has demonstrated the power of cross-and multi-ancestry genetic instruments to predict cardiometabolic traits [76] and further work is needed to improve our understanding of cross-ancestry and ancestry specific genetic risk for multimorbidity. We also note that a recent study of a similar albeit more restricted physical-mental health multimorbidity cluster [77], reported a PRS with similar performance to ICM-MM_PRS_, which suggests we are near the ceiling for prediction of heterogeneous multimorbidity clusters via polygenic risk scores rather than a limitation of our instrument per se.

In this study, we have investigated a specific form of internalising and cardiometabolic multimorbidity, and downstream health events that are common consequences of this cluster of conditions. Future work should focus on alternate definitions of multimorbidity, and examine the impact of other conditions such as dementia and cancers on multimorbidity trajectories [78,79]. Future work should also replicate our findings in larger cohorts, with genetic and healthcare data for a wider range of ancestral groups—this may be possible with the emerging availability of data from the ‘Our Future Health’ study [80]. This future research could also use trial emulation to assess existing models of clinical care, examining whether progression between multimorbidity states can be delayed or prevented.

Our work highlights the high burden of morbidity and multimorbidity in UK Pakistani and Bangladeshi populations, with clear social and demographic patterning of physical and mental health morbidity. The early onset of many of the conditions in our ICM-MM cluster, and ICM-MM itself suggests earlier implementation of the NHS health check may be required in these populations to reduce the impact of living with multiple long-term conditions, and future risk of major cardiovascular or renal events. Constructing genetic risk models for physical and mental health multimorbidity has a modest impact on determining risk of multimorbidity, and may be a useful adjunct to future precision-based care models using genotypic data.

We demonstrate the utility of taking a lifecourse approach to studying the development of multimorbidity, observing patterns such as the emergence of ICM-MM in young Bangladeshi women following a single internalising condition. This observation highlights the need for holistic care models that proactively screen for and intervene early in women with internalising conditions.

## Supporting information

S1 Text LINC statement on the ICM-MM phenotype and supporting references. (DOCX)

S2 Text Knot selection and plots of fitted vs observed survival for all transitions in the multi state model. (DOCX)

S3 Text Supplemental tables referred to in the main manuscript. **Table A:** A comparison of sociodemographic characteristics of people included in the analytic sample with the Genes & Health sample in East London. **Table B:** Frequency of internalising and cardiometabolic conditions, and cardiovascular/renal events in the analytic sample. **Table C:** Proportion of participants with internalising and cardiometabolic conditions, ICM-MM, CVR and all-cause mortality by age group. **Table D:** State occupation probabilities 10 years after baseline by gender and 10-year baseline age groups (women). **Table E:** State occupation probabilities 10 years after baseline by gender and 10-year baseline age groups (men). **Table F:** Contrasts in state occupation probabilities 10 years after baseline.
(DOCX)

S4 Text Supplemental figures referred to in the main manuscript. **Fig A:** Probabilities of CVE following ICM-MM over a 10-year window (men). **Fig B:** Probabilities of CVE following ICM-MM over a 10-year window (women). **Fig C:** Contrast in probability of CVE following CMD → INT vs INT → CMD(reference) 10 years after ICM-MM onset (men). **Fig D:** Contrast in probability of CVE following CMD → INT vs INT → CMD(reference) 10 years after ICM-MM onset (women).
(DOCX)

S1 Checklist Checklist of items that should be included in reports of cohort studies (The Strengthening the Reporting of Observational Studies in Epidemiology (STROBE) Statement: guidelines for reporting observational studies), original from https://www.equator-network.org/reporting-guidelines/strobe/). (DOCX)

## Abbreviations

AIC: Akaike information criterion
APC: Admitted Patient Care
CRD: Civil Registrations data
CVR: cardiovascular or renal events
HES: Hospital Episode Statistics
G&H: Genes & Health
IMD: Index of Multiple Deprivation
LINC: Lifespan Multimorbidity Research Collaborative
NHS: National Health Service
ONS: Office of National Statistics
PPI: Patient and Public Involvement
PRS: polygenic risk score
SNPs: single-nucleotide polymorphisms
