# Supplementary material for: Genetic and sociodemographic factors associated with trajectories of physical and mental health multimorbidity in a South Asian cohort in the UK: A multistate modelling analysis
Source: PLoS Med. 2026 Jul 9;23(7):e1004844. doi: 10.1371/journal.pmed.1004844 (PMC13349187; doi:10.1371/journal.pmed.1004844)
Supplement: S1 Text — (DOCX) [file pmed.1004844.s001.docx]

**LINC Statement:**

*“Our vision is to characterize the development of Physical and Mental Health Multimorbidity across the lifespan and identify opportunities for prevention and intervention. We will examine key genetic and environmental contributors Physical and Mental Health Multimorbidity development and investigate health inequalities based on ethnicity and sex.”*

The ICM-MM definition is based on broad LINC collaboration and discussion across key stakeholders involved in the research of multiple long-term conditions. The definition of ICM-MM used within LINC is bespoke and designed to both be practical (in terms of assessment, clinical gravity and frequency) and also to be a recognisable entity against the focus phenotype/outcomes of the LINC project.

The LINC definition of multi-morbidity (ICM-MM) is the result of discussion with primary and secondary care doctors, patient and public involvement advisors, as well as the broader research team. It is not feasible to have a single multi-morbidity definition that meets all requirements, so the one described seeks to be an acceptable compromise in terms of having face validity from a clinical and lay perspective, and being suitable for implementation from a research perspective.

The conditions included in the definition are restricted to those that generally occur earlier in life than established cardiovascular disease, and therefore can be followed for a longer period. The conditions are generally considered to be important risk factors for atherosclerosis, but do not include established atherosclerotic disease (e.g. stroke, ischaemic heart disease) or other cardiovascular disease (e.g. atrial fibrillation). The conditions do not include other long-term conditions for which atherosclerotic risk is generally a secondary concern (e.g. rheumatoid arthritis). Additionally, they do not include those risk factors for atherosclerosis that are predominantly behaviour related (e.g. smoking, lack of physical activity).

***ICM-MM Definition***

**Internalising conditions** are defined as the presence of at least one of the following **three** conditions, identified by participant self-report or the presence of at least one relevant **diagnostic or administrative clinical code** within primary or secondary care electronic health records (EHRs)

- Depression
- Anxiety
- Somatoform disorder

Somatoform disorders have been included in our definition due to high genetic,[^1^](https://paperpile.com/c/AzCT9V/63fd) diagnostic,[^2,3^](https://paperpile.com/c/AzCT9V/tYnI+GKjo) and symptomatic overlap with depression and anxiety - particularly in UK south Asian populations.[^4^](https://paperpile.com/c/AzCT9V/v4ZC)

**Cardiometabolic conditions** will be more precisely described using the term “cardiometabolic risk-factor conditions”, and is defined as the presence of at least one of the following **five** conditions, identified by participant self-report or the presence of at least one relevant **diagnostic or administrative clinical code** within primary or secondary care electronic health records (EHRs):

· Type 2 diabetes

· Hypertension

· Chronic kidney disease

· Obesity

· Dyslipidaemia

Additional references

1. [Fominykh V, Jaholkowski P, Shadrin AA, Koch E, Luitva LB, Mikkelsen DH, et al. Genome-wide study of somatic symptom and related disorders identifies novel genomic loci and map genetic architecture [Internet]. medRxiv. 2025. p. 2025.07.16.25331639. Available from:](http://paperpile.com/b/AzCT9V/63fd) <http://dx.doi.org/10.1101/2025.07.16.25331639v1>

2. [Tesfaye M, Jaholkowski P, Shadrin AA, van der Meer D, Hindley GFL, Holen B, et al. Identification of novel genomic loci for anxiety symptoms and extensive genetic overlap with psychiatric disorders. Psychiatry Clin Neurosci. 2024 Dec;78(12):783–91.](http://paperpile.com/b/AzCT9V/tYnI)

3. [Eto F, Samuel M, Henkin R, Mahesh M, Ahmad T, Angdembe A, et al. Ethnic differences in early onset multimorbidity and associations with health service use, long-term prescribing, years of life lost, and mortality: A cross-sectional study using clustering in the UK Clinical Practice Research Datalink. PLoS Med. 2023 Oct;20(10):e1004300.](http://paperpile.com/b/AzCT9V/GKjo)

4. [Mumford DB, Bavington JT, Bhatnagar KS, Hussain Y, Mirza S, Naraghi MM. The Bradford Somatic Inventory. A multi-ethnic inventory of somatic symptoms reported by anxious and depressed patients in Britain and the Indo-Pakistan subcontinent. Br J Psychiatry. 1991 Mar;158:379–86.](http://paperpile.com/b/AzCT9V/v4ZC)
