# Supplementary material for: Genetic and sociodemographic factors associated with trajectories of physical and mental health multimorbidity in a South Asian cohort in the UK: A multistate modelling analysis
Source: PLoS Med. 2026 Jul 9;23(7):e1004844. doi: 10.1371/journal.pmed.1004844 (PMC13349187; doi:10.1371/journal.pmed.1004844)

Supplemental modelling: Knot selection and plots of fitted vs observed survival for all transitions in the multi state model.

Table of Contents

[Transition 1 model fit 2](#_Toc224906939)

[Transition 2 model fit 3](#_Toc224906940)

[Transition 3 model fit 4](#_Toc224906941)

[Transition 4 model fit 5](#_Toc224906942)

[Transition 5 model fit 6](#_Toc224906943)

[Transition 6 model fit 7](#_Toc224906944)

[Transition 7 model fit 8](#_Toc224906945)

[Transition 8 model fit 9](#_Toc224906946)

[Transition 9 model fit 10](#_Toc224906947)

[Transition 10 model fit 11](#_Toc224906948)

[Transition 11 model fit 12](#_Toc224906949)

[Transition 12 model fit 13](#_Toc224906950)

[Transition 13 model fit 14](#_Toc224906951)

[Transition 14 model fit 15](#_Toc224906952)

## Transition 1 model fit

23,554 individuals: 3 knots selected


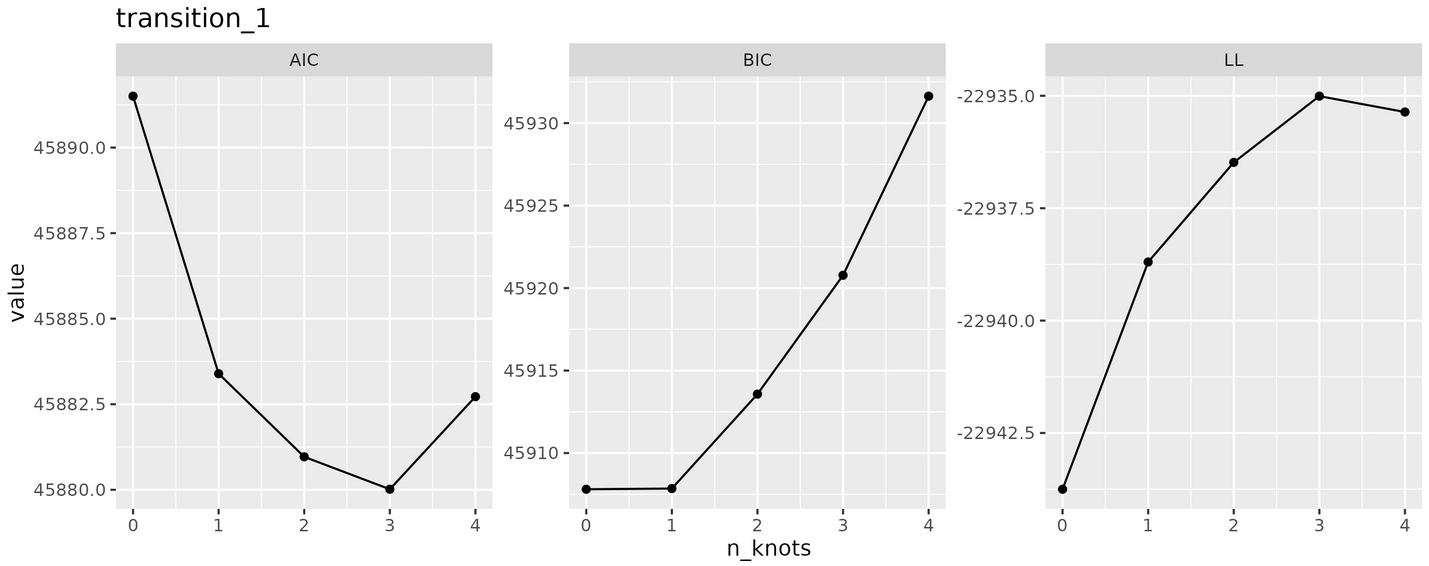


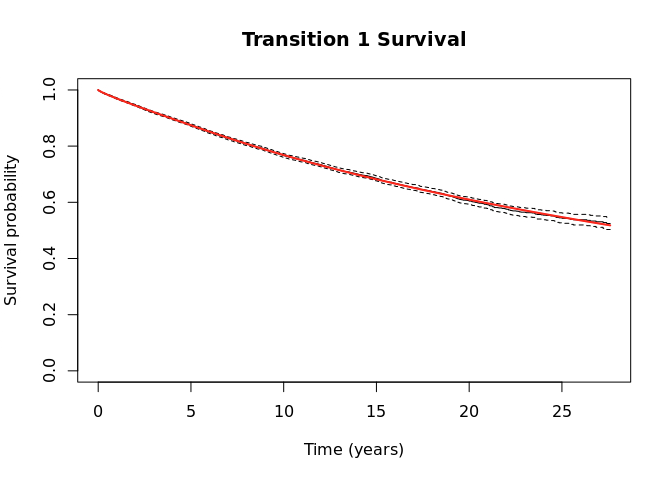


## Transition 2 model fit

23,554 individuals: 2 knots selected


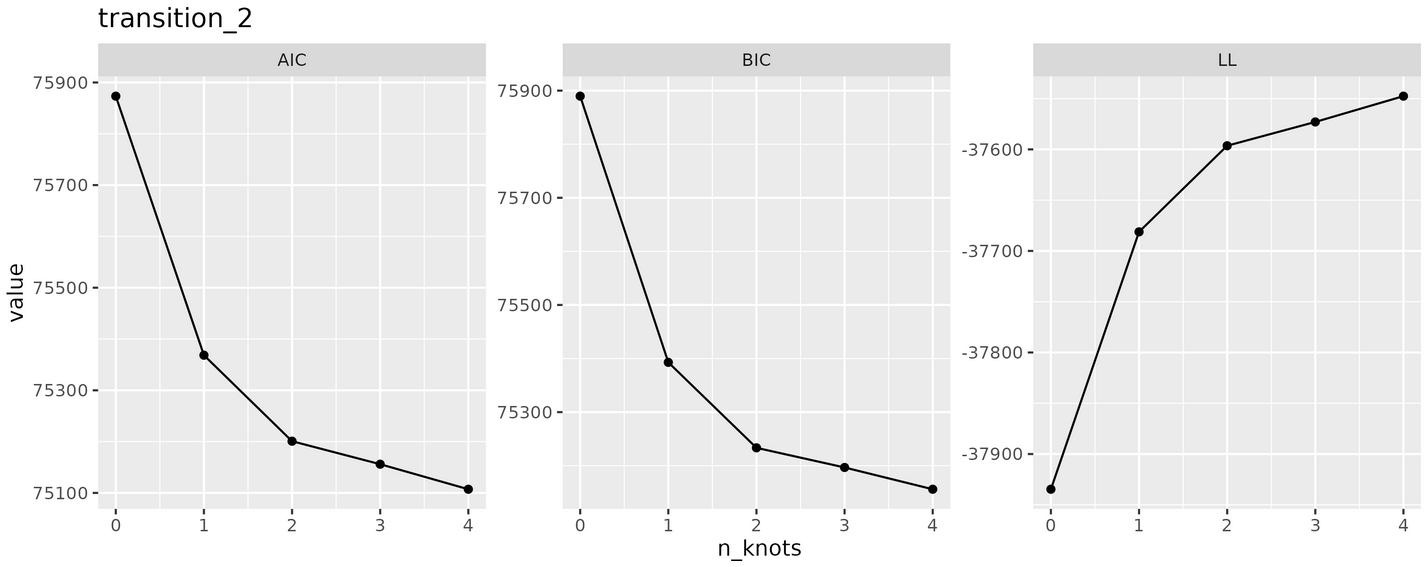


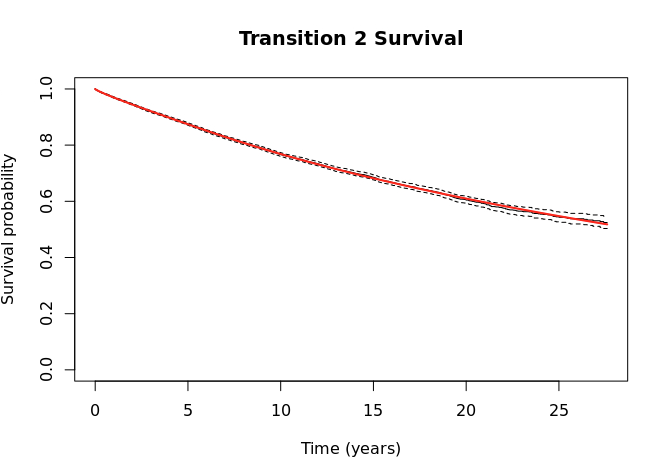


## Transition 3 model fit

23,554 individuals: 2 knots selected


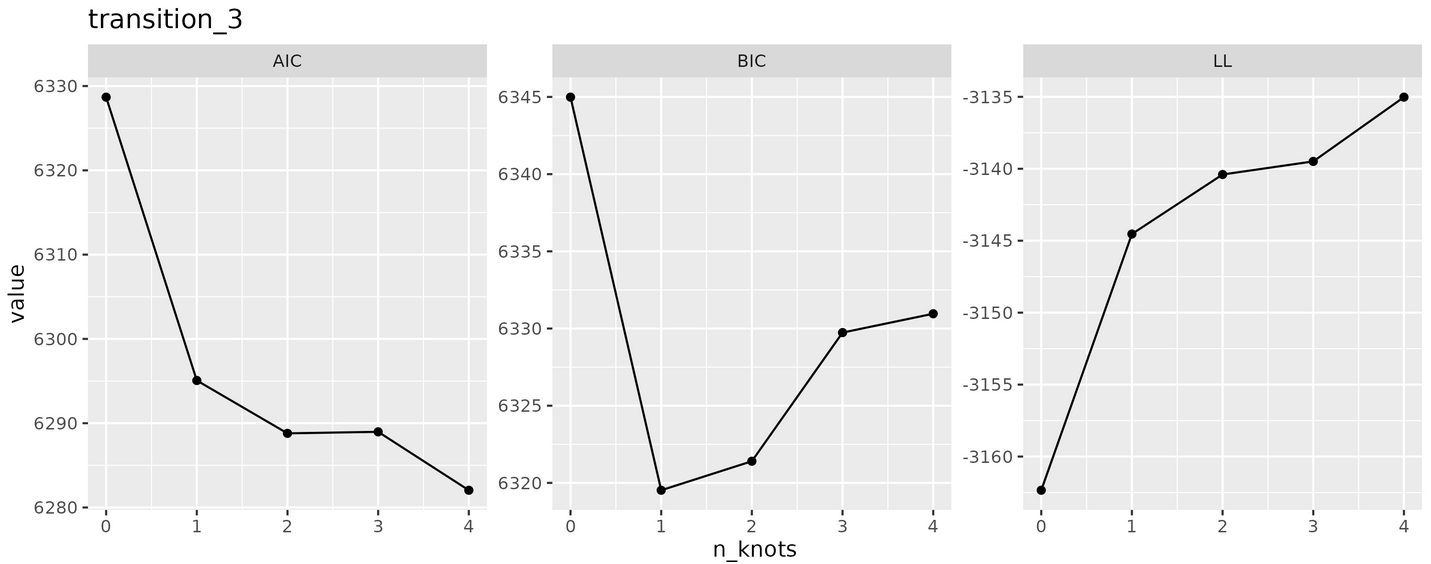


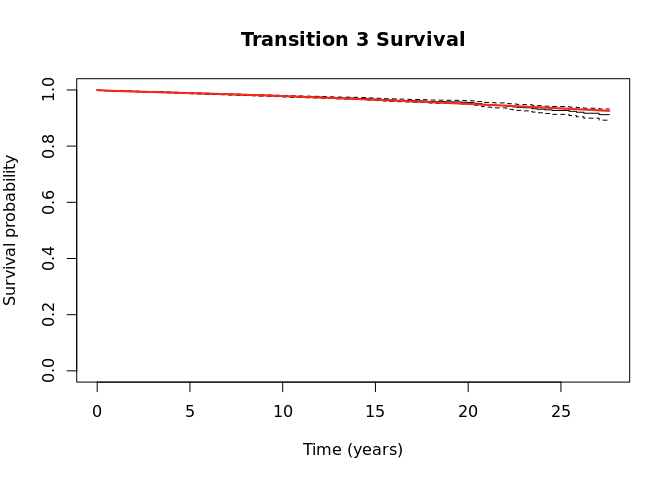


## Transition 4 model fit

23,554 individuals: 0 knots selected


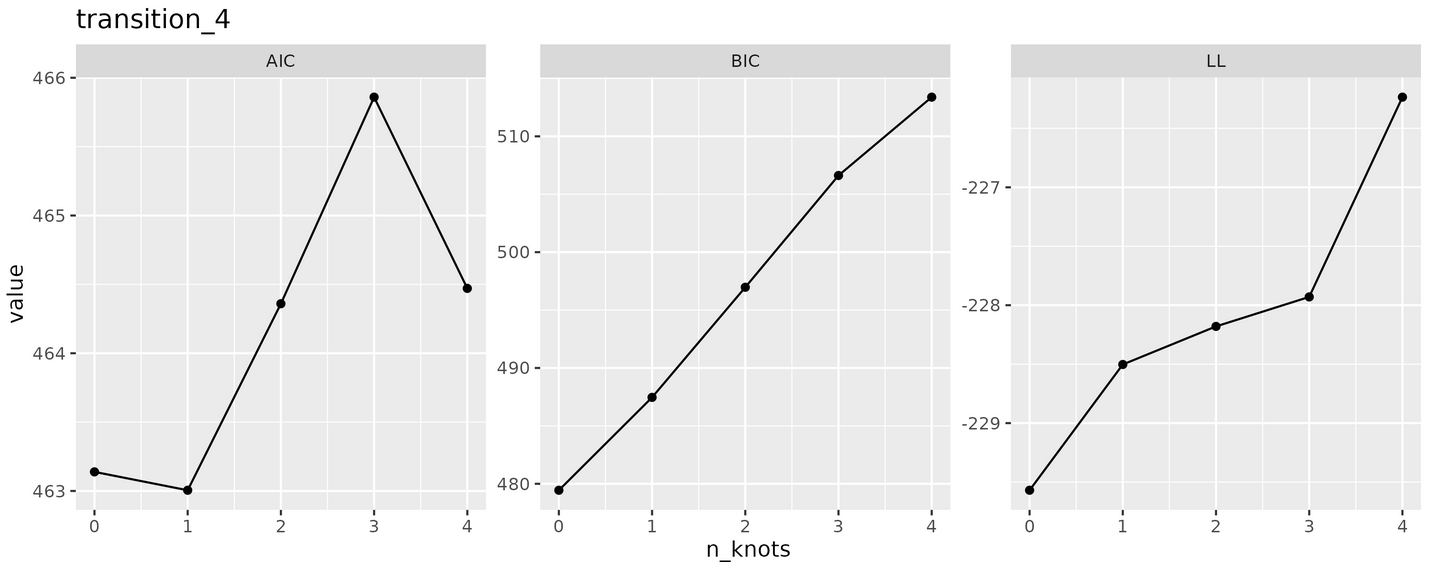


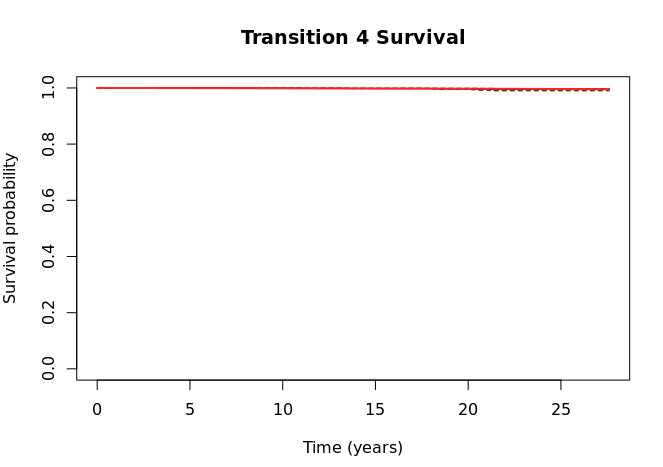


## Transition 5 model fit

4,473 individuals: 1 knot selected


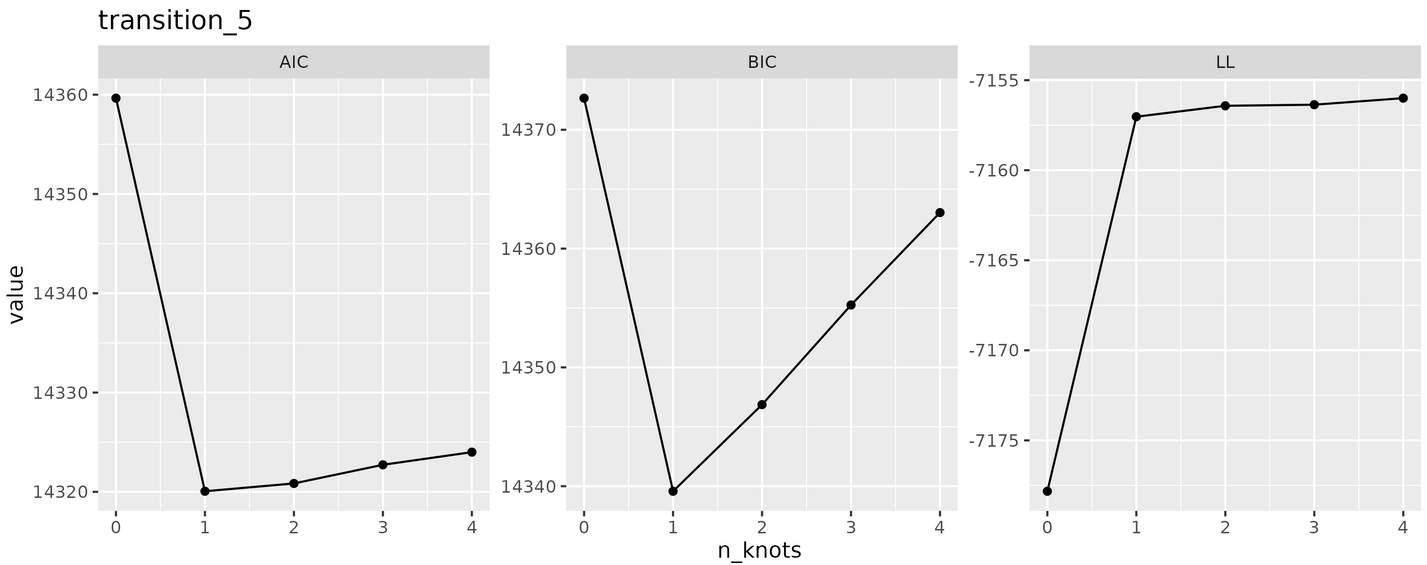


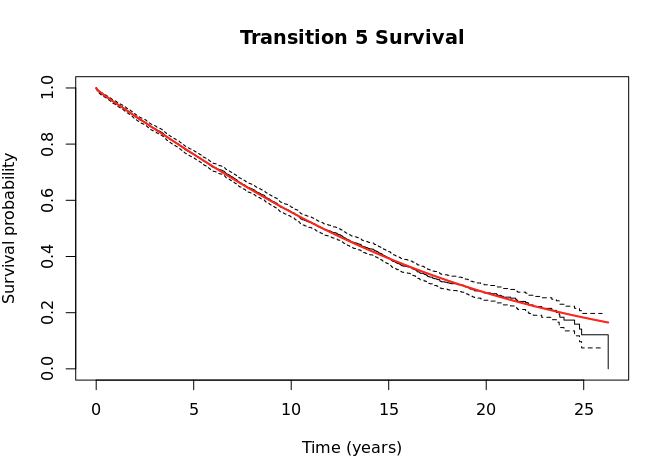


## Transition 6 model fit

4,473 individuals: 1 knot selected


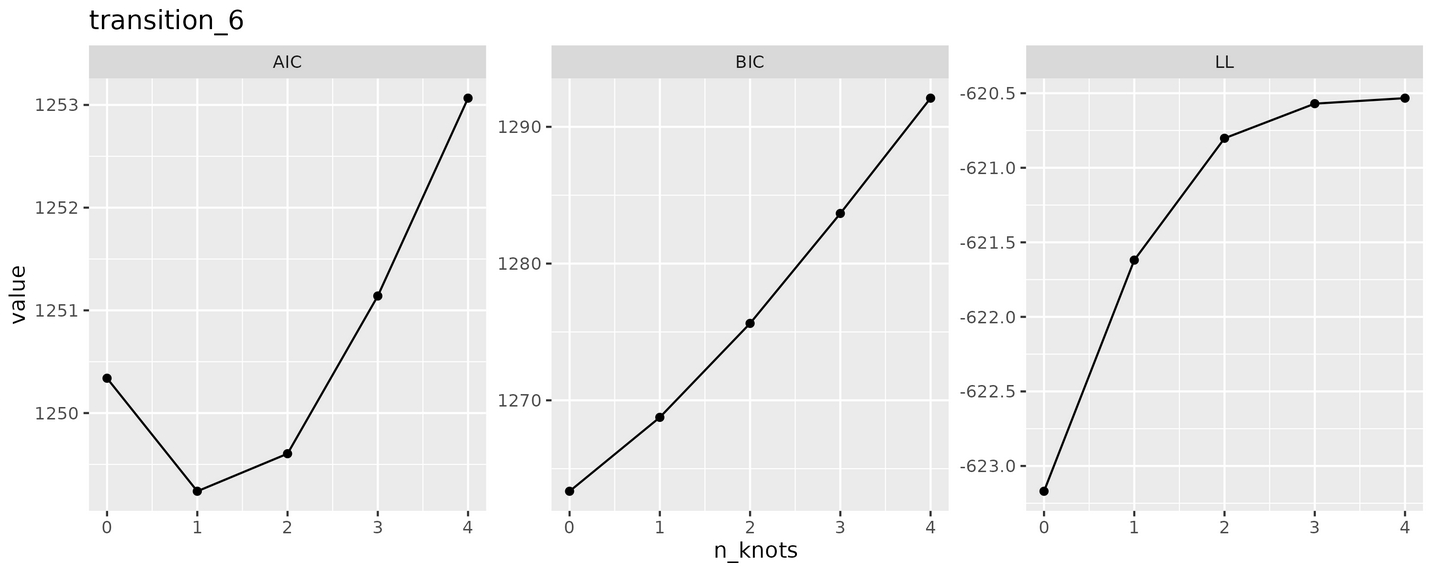


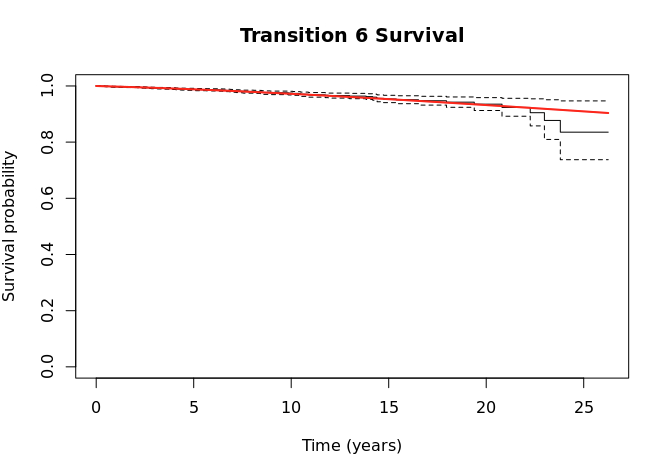


## Transition 7 model fit

4,473 individuals: 0 knots selected


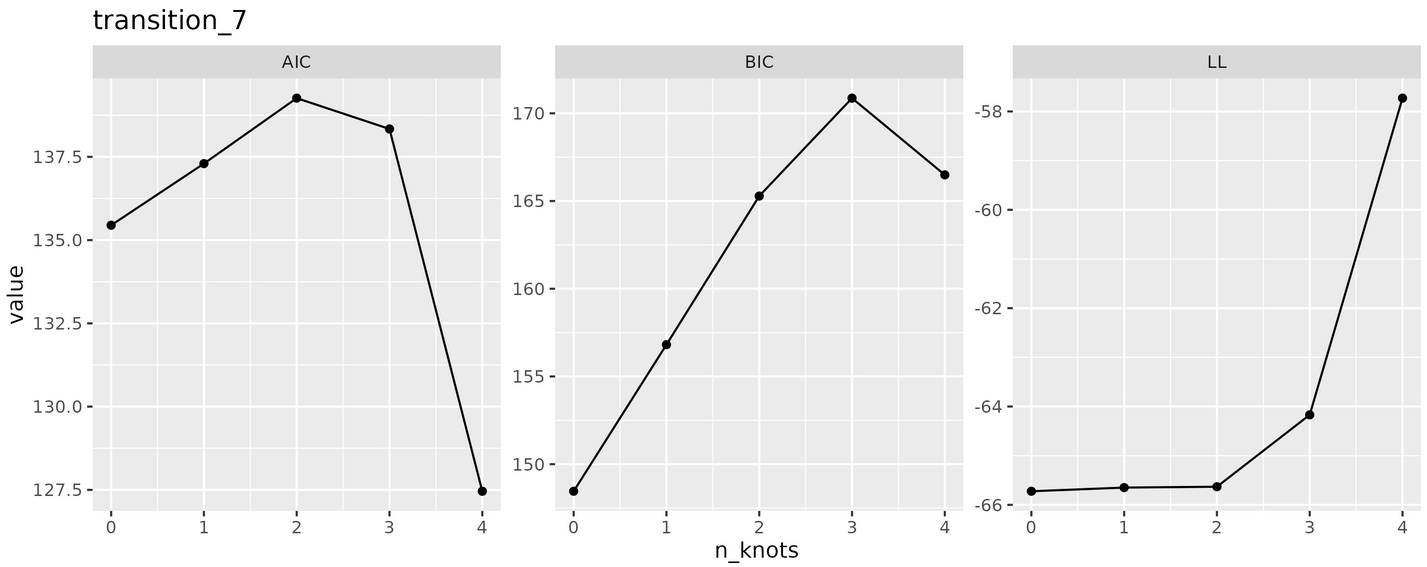


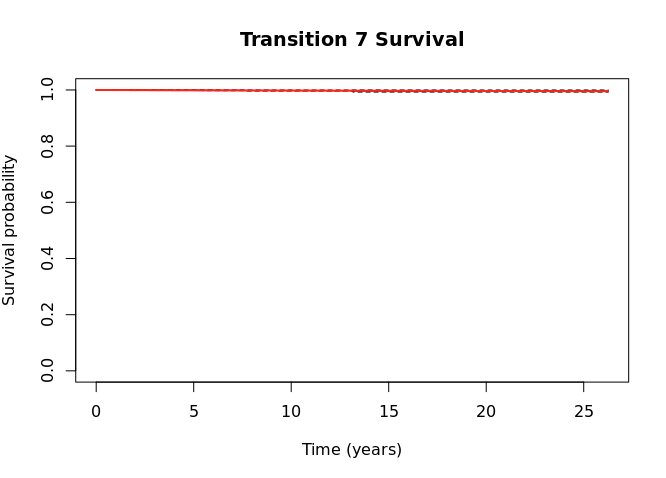


## Transition 8 model fit

8,214 individuals: 2 knots selected


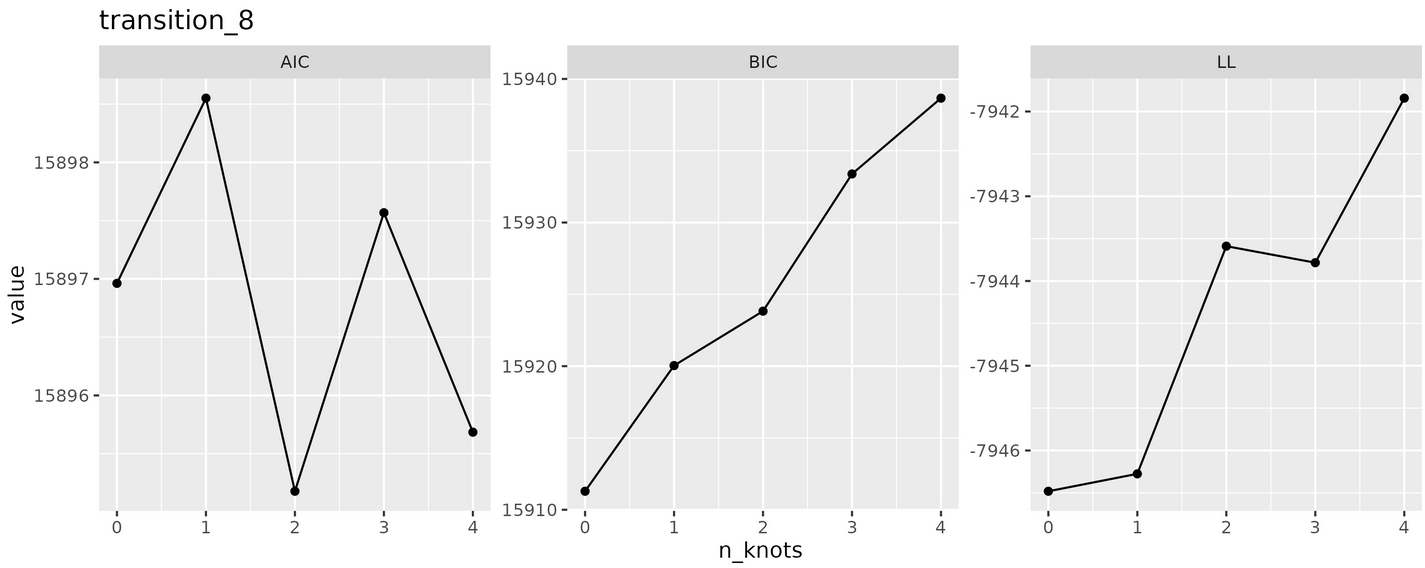


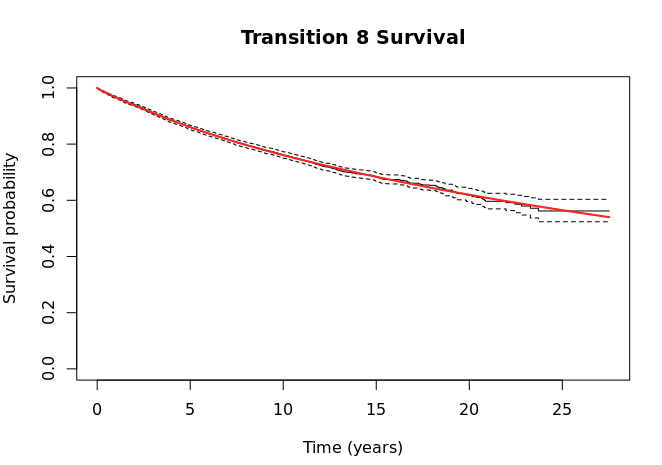


## Transition 9 model fit

8,214 individuals: 2 knots selected


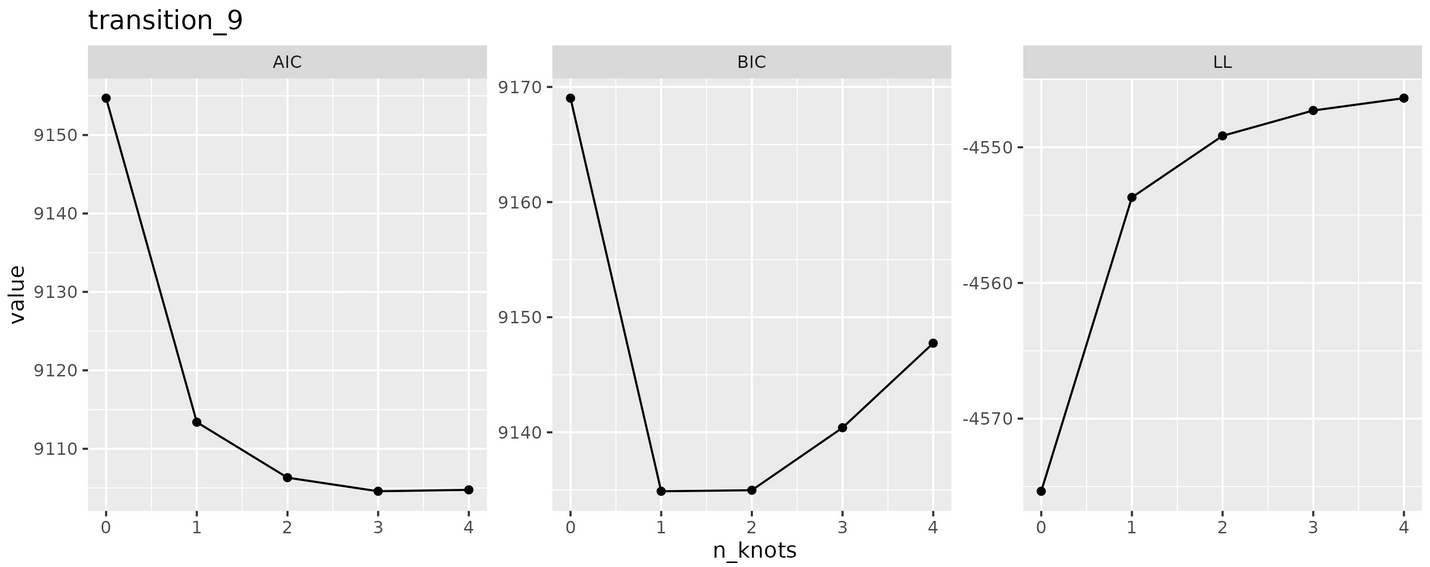


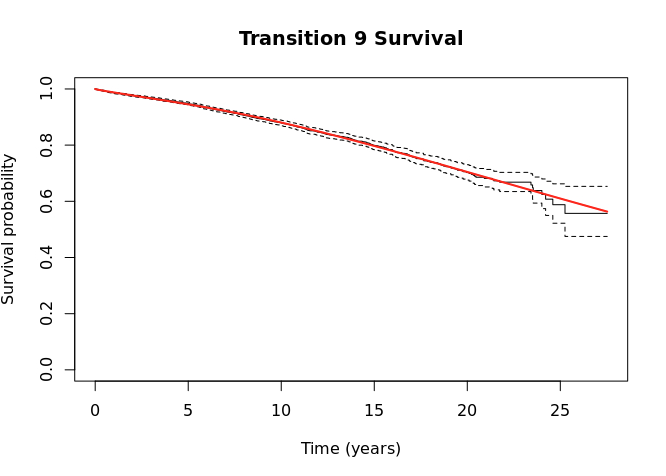


## Transition 10 model fit

8,214 individuals: 2 knots selected


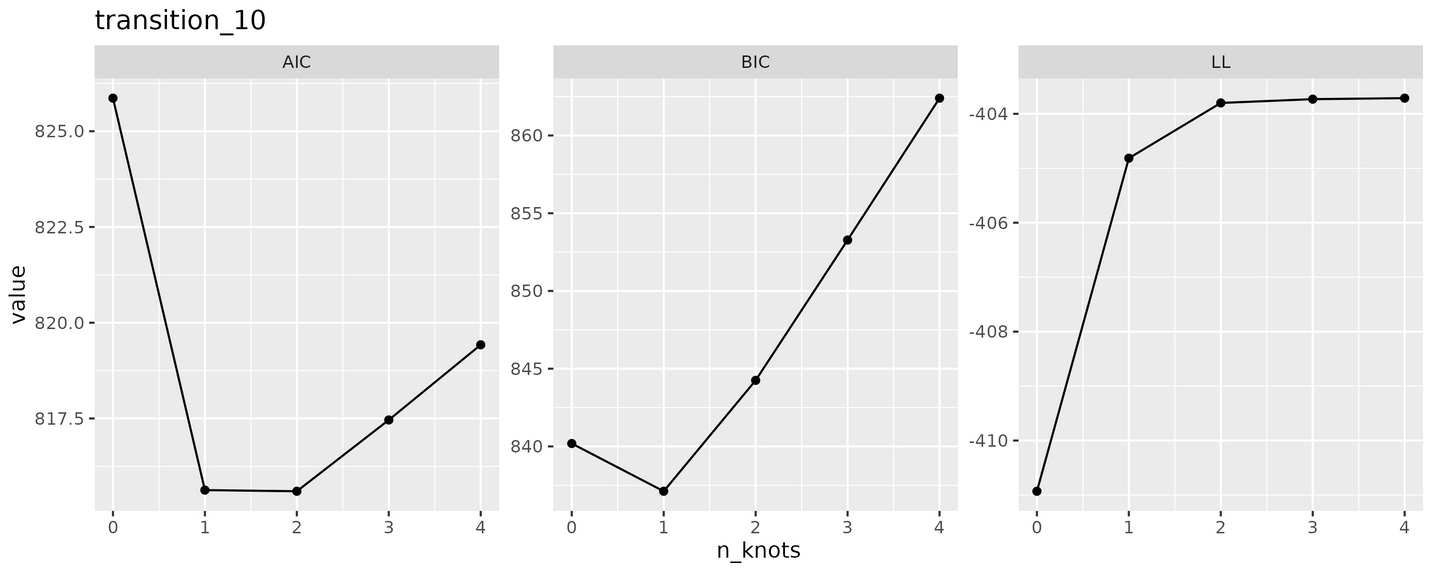


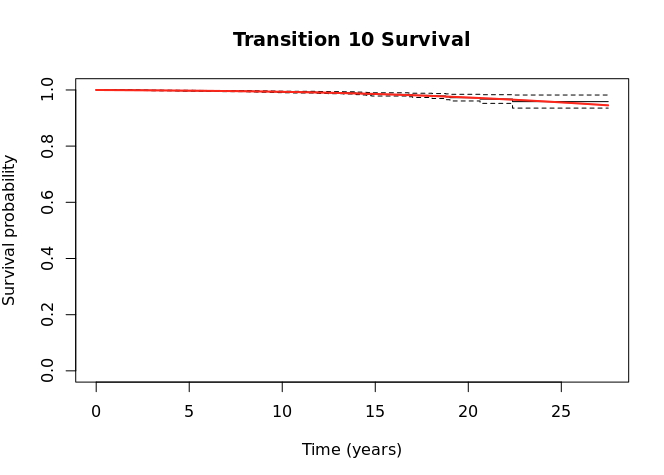


## Transition 11 model fit

1,684 individuals: 1 knot selected


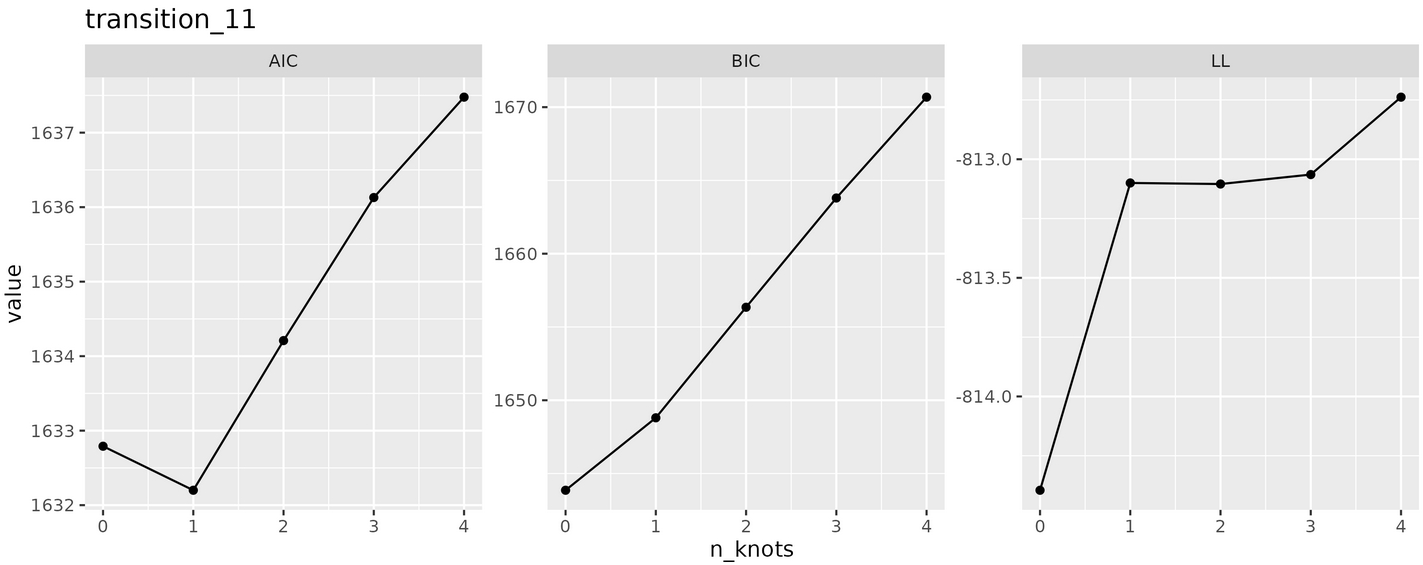


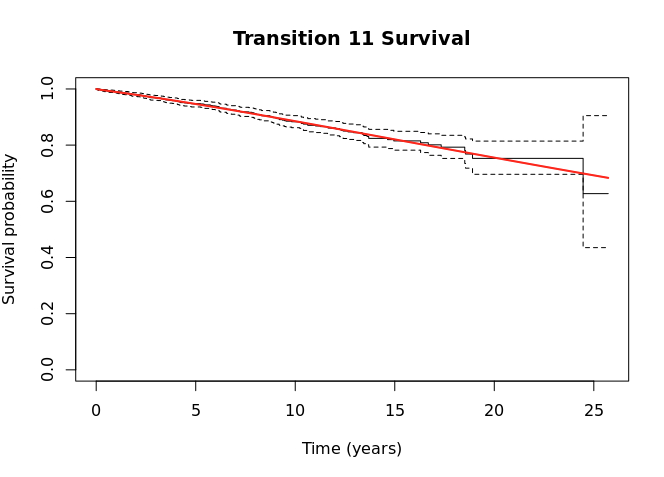


## Transition 12 model fit

1,684 individuals: 0 knots selected


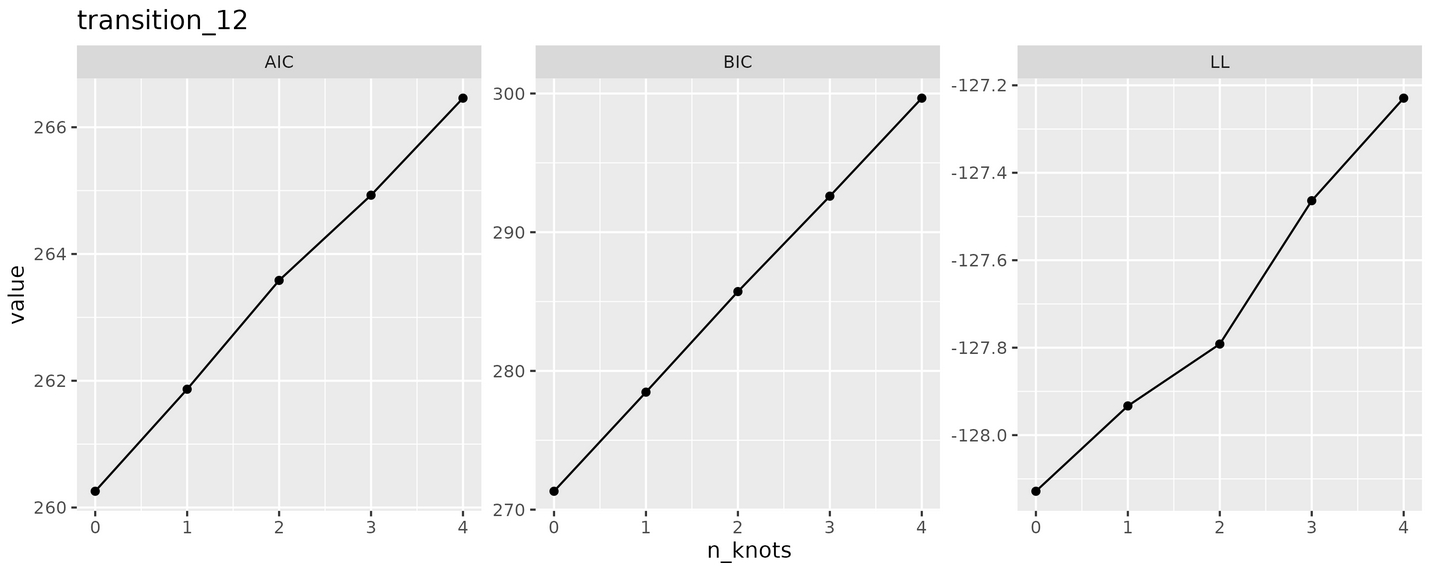


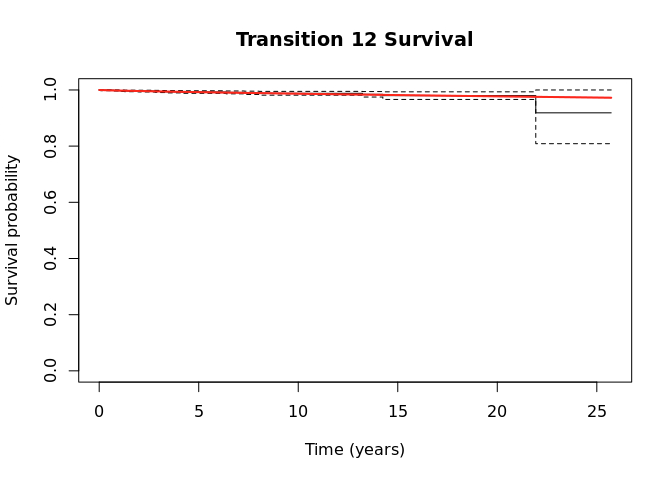


## Transition 13 model fit

1,474 individuals: 2 knots selected


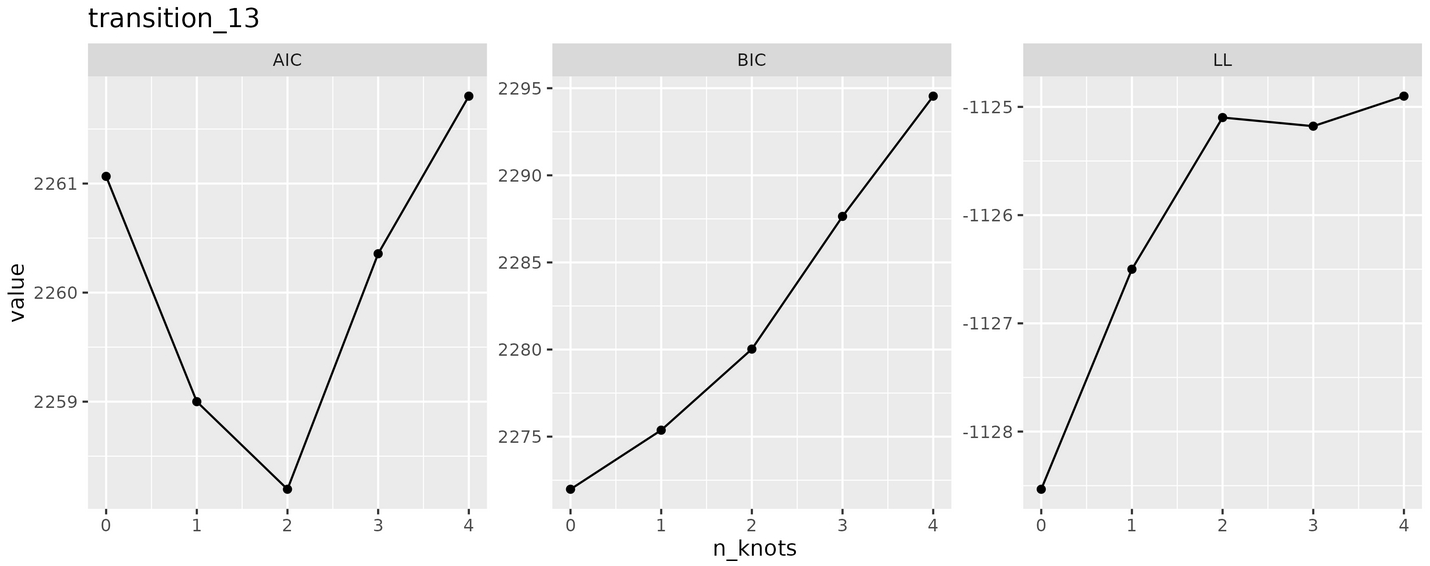


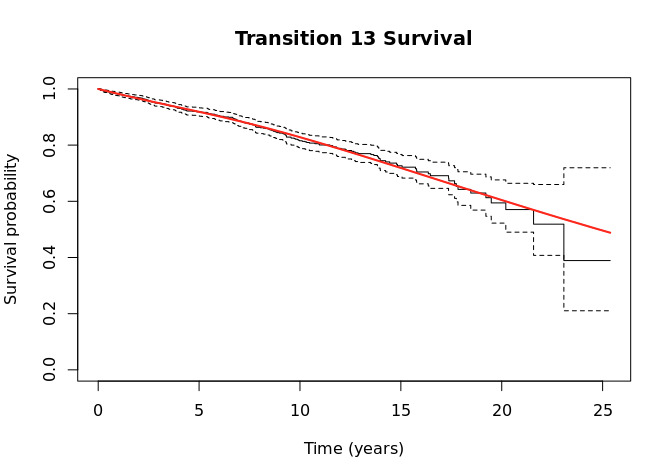


## Transition 14 model fit

1,474 individuals: 1 knot selected


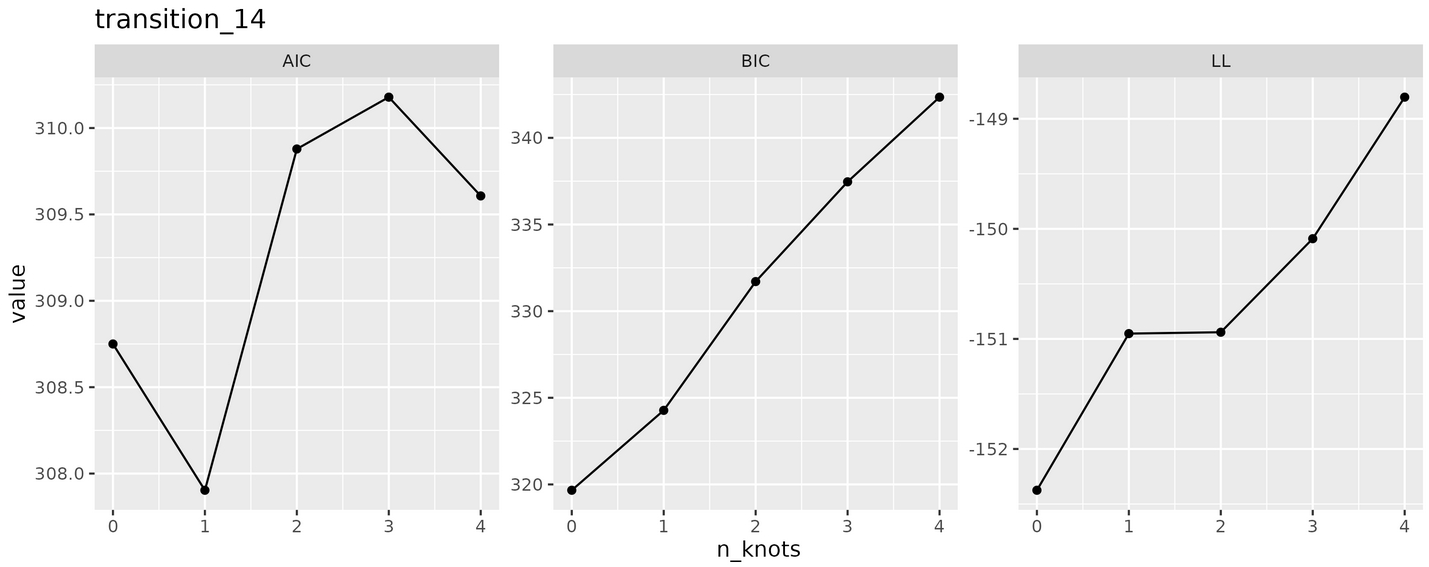


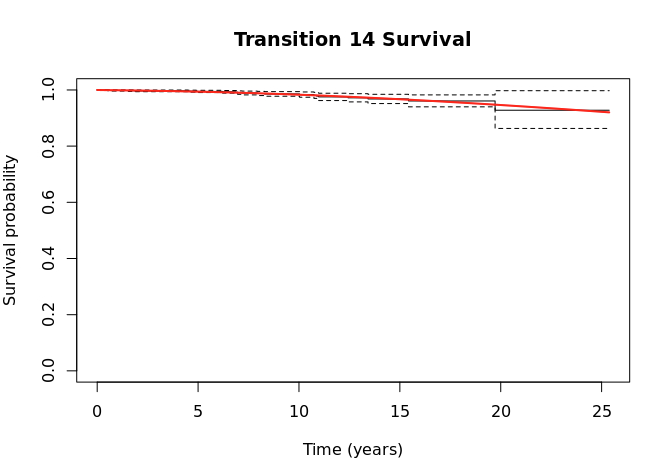

Supplement: S2 Text — (DOCX) [file pmed.1004844.s002.docx]
