# Supplementary material for: Genetic and sociodemographic factors associated with trajectories of physical and mental health multimorbidity in a South Asian cohort in the UK: A multistate modelling analysis
Source: PLoS Med. 2026 Jul 9;23(7):e1004844. doi: 10.1371/journal.pmed.1004844 (PMC13349187; doi:10.1371/journal.pmed.1004844)
Supplement: S3 Text — Table A: A comparison of sociodemographic characteristics of people included in the analytic sample with the Genes & Health sample in East London. Table B: Frequency of internalising and cardiometabolic conditions, and cardiovascular/renal events in the analytic sample. Table C: Proportion of participants with internalising and cardiometabolic conditions, ICM-MM, CVR and all-cause mortality by age group. Table D: State occupation probabilities 10 years after baseline by gender and 10-year baseline age groups (women). Table E: State occupation probabilities 10 years after baseline by gender and 10-year baseline age groups (men). Table F: Contrasts in state occupation probabilities 10 years after baseline. (DOCX) [file pmed.1004844.s003.docx]

**S3 Text: Supplemental tables**

Table of Contents

[Supplemental table A: A comparison of sociodemographic characteristics of people included in the analytic sample with the Genes & Health sample in East London 2](#_Toc232001712)

[Supplemental table B: Frequency of internalising and cardiometabolic conditions, and cardiovascular / renal events in the analytic sample 3](#_Toc232001713)

[Supplemental table C: Proportion of participants with internalising and cardiometabolic conditions, ICM-MM, CVR and all-cause mortality by age group. 4](#_Toc232001714)

[Supplemental table D: State occupation probabilities ten years after baseline by gender and ten-year baseline age groups (women) 5](#_Toc232001715)

[Supplemental table E: State occupation probabilities ten years after baseline by gender and ten-year baseline age groups (men) 6](#_Toc232001716)

[Supplemental table F: Contrasts in state occupation probabilities 10 years after baseline 7](#_Toc232001717)

### Supplemental table A: A comparison of sociodemographic characteristics of people included in the analytic sample with the Genes & Health sample in East London

|  | **Excluded** | **Included** | **Overall sample*** |
| --- | --- | --- | --- |
|  | **(N=22002)** | **(N=23554)** | **(N=45556)** |
| **Self-reported Gender** |  |  |  |
| Female | 11884 (54.0%) | 12934 (54.9%) | 24818 (54.5%) |
| Male | 10118 (46.0%) | 10620 (45.1%) | 20738 (45.5%) |
| **Self-reported year of birth** | |  |  |
| Median [Q1,Q3] | 1980 [1970,1990] | 1980 [1970,1990] | 1980 [1970,1990] |
| Mean [SD] | 1970 [15.5] | 1980 [12.5] | 1980 [14.3] |
| **Self-reported ethnicity** | |  |  |
| Bangladeshi | 14870 (67.6%) | 15451 (65.6%) | 30321 (66.6%) |
| Other | 194 (0.9%) | 0 (0%) | 194 (0.4%) |
| Pakistani | 6761 (30.7%) | 8103 (34.4%) | 14864 (32.6%) |
| Missing | 177 (0.8%) | 0 (0%) | 177 (0.4%) |
| **Smoking status**** |  |  |  |
| Never | 13095 (59.5%) | 16838 (71.5%) | 29933 (65.7%) |
| Ever | 7650 (34.8%) | 6716 (28.5%) | 14366 (31.5%) |
| Missing | 1257 (5.7%) | 0 (0%) | 1257 (2.8%) |
| **IMD**** |  |  |  |
| 1 | 6023 (27.4%) | 6415 (27.2%) | 12438 (27.3%) |
| 2 | 11939 (54.3%) | 12814 (54.4%) | 24753 (54.3%) |
| 3 | 3046 (13.8%) | 3274 (13.9%) | 6320 (13.9%) |
| 4 | 773 (3.5%) | 835 (3.5%) | 1608 (3.5%) |
| 5 | 198 (0.9%) | 216 (0.9%) | 414 (0.9%) |
| Missing | 23 (0.1%) | 0 (0%) | 23 (0.1%) |
| * Participants with primary care data - accessible for practices in East London only  ** IMD data and smoking status taken from primary care health records | | | |

### Supplemental table B: Frequency of internalising and cardiometabolic conditions, and cardiovascular / renal events in the analytic sample

|  | **Female** | **Male** | **Overall** |
| --- | --- | --- | --- |
|  | (N=12934) | (N=10620) | (N=23554) |
| **Cardiometabolic conditions** |  |  |  |
| Dyslipidaemia | 1289 (10.0%) | 1802 (17.0%) | 3091 (13.1%) |
| Obesity | 2116 (16.4%) | 605 (5.7%) | 2721 (11.6%) |
| Hypertension | 1118 (8.6%) | 848 (8.0%) | 1966 (8.3%) |
| Type 2 Diabetes | 939 (7.3%) | 1019 (9.6%) | 1958 (8.3%) |
| Chronic Kidney Disease | 54 (0.4%) | 108 (1.0%) | 162 (0.7%) |
| **Internalising conditions** |  |  |  |
| Anxiety and phobia | 1736 (13.4%) | 884 (8.3%) | 2620 (11.1%) |
| Depression | 1565 (12.1%) | 779 (7.3%) | 2344 (10.0%) |
| Somatoform and dissociative disorders | 732 (5.7%) | 252 (2.4%) | 984 (4.2%) |
| **Cardiovascular and renal events** |  |  |  |
| Atrial fibrillation and flutter | 43 (0.3%) | 43 (0.4%) | 86 (0.4%) |
| Peripheral arterial disease | 15 (0.1%) | 42 (0.4%) | 57 (0.2%) |
| End stage renal disease | 18 (0.1%) | 17 (0.2%) | 35 (0.1%) |
| Cerebrovascular disease | 80 (0.6%) | 128 (1.2%) | 208 (0.9%) |
| Death due to CVR | 6 (0.0%) | 14 (0.1%) | 20 (0.1%) |
| Heart failure | 57 (0.4%) | 45 (0.4%) | 102 (0.4%) |
| Coronary heart disease | 316 (2.4%) | 698 (6.6%) | 1014 (4.3%) |

### Supplemental table C: Proportion of participants with internalising and cardiometabolic conditions, ICM-MM, CVR and all-cause mortality by age group.

| **Baseline age** | | | | | | |
| --- | --- | --- | --- | --- | --- | --- |
|  | **20** | **30** | **40** | **50** | **60** | **70** |
|  | **(N=10810)** | **(N=8062)** | **(N=3591)** | **(N=832)** | **(N=219)** | **(N=40)** |
| **Follow up (years)** |  |  |  |  |  |  |
| Mean (SD) | 13.0 (7.29) | 10.8 (7.03) | 10.7 (7.50) | 11.5 (7.99) | 11.6 (7.60) | 7.45 (5.33) |
| **Internalising conditions** | |  |  |  |  |  |
| Yes | 3222 (29.8%) | 1795 (22.3%) | 704 (19.6%) | 175 (21.0%) | 46 (21.0%) | 6 (15.0%) |
| No | 7588 (70.2%) | 6267 (77.7%) | 2887 (80.4%) | 657 (79.0%) | 173 (79.0%) | 34 (85.0%) |
| **Cardiometabolic conditions** | |  |  |  |  |  |
| Yes | 3295 (30.5%) | 3713 (46.1%) | 2119 (59.0%) | 580 (69.7%) | 167 (76.3%) | 24 (60.0%) |
| No | 7515 (69.5%) | 4349 (53.9%) | 1472 (41.0%) | 252 (30.3%) | 52 (23.7%) | 16 (40.0%) |
| **ICM-MM via CMD->INT** | |  |  |  |  |  |
| Yes | 505 (4.7%) | 534 (6.6%) | 308 (8.6%) | 96 (11.5%) | 28 (12.8%) | 4 (10.0%) |
| No | 10305 (95.3%) | 7528 (93.4%) | 3283 (91.4%) | 736 (88.5%) | 191 (87.2%) | 36 (90.0%) |
| **ICM-MM via INT->CMD** | |  |  |  |  |  |
| Yes | 819 (7.6%) | 557 (6.9%) | 235 (6.5%) | 58 (7.0%) | 14 (6.4%) | SDC |
| No | 9991 (92.4%) | 7505 (93.1%) | 3356 (93.5%) | 774 (93.0%) | 205 (93.6%) | SDC |
| **Cardiovascular or renal event*** | |  |  |  |  |  |
| Yes | 232 (2.1%) | 441 (5.5%) | 505 (14.1%) | 231 (27.8%) | 89 (40.6%) | 24 (60.0%) |
| No | 10578 (97.9%) | 7621 (94.5%) | 3086 (85.9%) | 601 (72.2%) | 130 (59.4%) | 16 (40.0%) |
| **Died**** |  |  |  |  |  |  |
| Yes | 22 (0.2%) | 25 (0.3%) | 23 (0.6%) | 13 (1.6%) | 16 (7.3%) | SDC |
| No | 10788 (99.8%) | 8037 (99.7%) | 3568 (99.4%) | 819 (98.4%) | 203 (92.7%) | SDC |
| * Including deaths due to CVR, ** All other cause mortality (ONS civil registrations) SDC: statistical disclosure control <5 cases | | | | | | |

### Supplemental table D: State occupation probabilities ten years after baseline by gender and ten-year baseline age groups (women)

| **Age group** | **Health state** | **Probability [estimate]** | **95%CI:[low]** | **95%CI:[up]** |
| --- | --- | --- | --- | --- |
| **20 years old** | Healthy | 0.57 | 0.54 | 0.58 |
|  | INT | 0.25 | 0.23 | 0.28 |
|  | CMD | 0.10 | 0.10 | 0.12 |
|  | INT->CMD | 0.04 | 0.02 | 0.05 |
|  | CMD->INT | 0.03 | 0.03 | 0.04 |
|  | CVR | 0.00 | 0.00 | 0.01 |
|  | Death | 0.00 | 0.00 | 0.00 |
| **30 years old** | Healthy | 0.44 | 0.42 | 0.46 |
|  | INT | 0.18 | 0.17 | 0.21 |
|  | CMD | 0.24 | 0.23 | 0.26 |
|  | INT->CMD | 0.07 | 0.04 | 0.07 |
|  | CMD->INT | 0.06 | 0.05 | 0.07 |
|  | CVR | 0.01 | 0.01 | 0.02 |
|  | Death | 0.00 | 0.00 | 0.00 |
| **40 years old** | Healthy | 0.25 | 0.24 | 0.27 |
|  | INT | 0.11 | 0.09 | 0.14 |
|  | CMD | 0.43 | 0.41 | 0.45 |
|  | INT->CMD | 0.07 | 0.04 | 0.09 |
|  | CMD->INT | 0.09 | 0.08 | 0.10 |
|  | CVR | 0.04 | 0.03 | 0.04 |
|  | Death | 0.00 | 0.00 | 0.01 |
| **50 years old** | Healthy | 0.17 | 0.15 | 0.18 |
|  | INT | 0.06 | 0.05 | 0.09 |
|  | CMD | 0.50 | 0.49 | 0.54 |
|  | INT->CMD | 0.08 | 0.04 | 0.09 |
|  | CMD->INT | 0.09 | 0.09 | 0.11 |
|  | CVR | 0.08 | 0.07 | 0.09 |
|  | Death | 0.01 | 0.00 | 0.01 |
| **60 years old** | Healthy | 0.11 | 0.09 | 0.13 |
|  | INT | 0.03 | 0.02 | 0.06 |
|  | CMD | 0.53 | 0.49 | 0.56 |
|  | INT->CMD | 0.07 | 0.04 | 0.08 |
|  | CMD->INT | 0.12 | 0.10 | 0.15 |
|  | CVR | 0.13 | 0.11 | 0.15 |
|  | Death | 0.02 | 0.01 | 0.04 |
| **70 years old** | Healthy | 0.06 | 0.04 | 0.08 |
|  | INT | 0.02 | 0.01 | 0.04 |
|  | CMD | 0.50 | 0.44 | 0.55 |
|  | INT->CMD | 0.04 | 0.02 | 0.07 |
|  | CMD->INT | 0.14 | 0.11 | 0.20 |
|  | CVR | 0.19 | 0.16 | 0.22 |
|  | Death | 0.05 | 0.03 | 0.09 |
| Probabilities for the male and female reference covariate pattern [Bangladeshi participant living in IMD quintile 1 (most deprived) with ICM-MMPRS z-score of 0 (‘average risk’), and a ‘never’ smoker age 40 at the start of follow-up] | | | | |

### Supplemental table E: State occupation probabilities ten years after baseline by gender and ten-year baseline age groups (men)

| **Age group** | **Health state** | **Probability [estimate]** | **95%CI:[low]** | **95%CI:[up]** |
| --- | --- | --- | --- | --- |
| **20 years old** | Healthy | 0.69 | 0.68 | 0.71 |
|  | INT | 0.14 | 0.13 | 0.16 |
|  | CMD | 0.12 | 0.11 | 0.13 |
|  | INT->CMD | 0.02 | 0.01 | 0.03 |
|  | CMD->INT | 0.02 | 0.01 | 0.02 |
|  | CVR | 0.01 | 0.01 | 0.01 |
|  | Death | 0.00 | 0.00 | 0.00 |
| **30 years old** | Healthy | 0.55 | 0.53 | 0.57 |
|  | INT | 0.10 | 0.09 | 0.12 |
|  | CMD | 0.26 | 0.24 | 0.28 |
|  | INT->CMD | 0.04 | 0.02 | 0.04 |
|  | CMD->INT | 0.03 | 0.03 | 0.04 |
|  | CVR | 0.02 | 0.02 | 0.03 |
|  | Death | 0.00 | 0.00 | 0.00 |
| **40 years old** | Healthy | 0.33 | 0.31 | 0.35 |
|  | INT | 0.06 | 0.05 | 0.08 |
|  | CMD | 0.46 | 0.43 | 0.47 |
|  | INT->CMD | 0.04 | 0.02 | 0.04 |
|  | CMD->INT | 0.05 | 0.04 | 0.06 |
|  | CVR | 0.07 | 0.06 | 0.08 |
|  | Death | 0.00 | 0.00 | 0.01 |
| **50 years old** | Healthy | 0.21 | 0.19 | 0.24 |
|  | INT | 0.03 | 0.03 | 0.05 |
|  | CMD | 0.52 | 0.48 | 0.54 |
|  | INT->CMD | 0.04 | 0.02 | 0.05 |
|  | CMD->INT | 0.05 | 0.04 | 0.06 |
|  | CVR | 0.15 | 0.12 | 0.17 |
|  | Death | 0.01 | 0.00 | 0.02 |
| **60 years old** | Healthy | 0.13 | 0.11 | 0.16 |
|  | INT | 0.02 | 0.01 | 0.03 |
|  | CMD | 0.52 | 0.47 | 0.54 |
|  | INT->CMD | 0.03 | 0.02 | 0.04 |
|  | CMD->INT | 0.06 | 0.04 | 0.07 |
|  | CVR | 0.22 | 0.20 | 0.26 |
|  | Death | 0.02 | 0.01 | 0.05 |
| **70 years old** | Healthy | 0.07 | 0.05 | 0.10 |
|  | INT | 0.01 | 0.00 | 0.02 |
|  | CMD | 0.46 | 0.40 | 0.50 |
|  | INT->CMD | 0.02 | 0.01 | 0.03 |
|  | CMD->INT | 0.06 | 0.03 | 0.08 |
|  | CVR | 0.34 | 0.28 | 0.39 |
|  | Death | 0.05 | 0.03 | 0.11 |
| Probabilities for the male and female reference covariate pattern [Bangladeshi participant living in IMD quintile 1 (most deprived) with ICM-MMPRS z-score of 0 (‘average risk’), and a ‘never’ smoker age 40 at the start of follow-up] | | | | |

### Supplemental table F: Contrasts in state occupation probabilities 10 years after baseline

| **Contrast** |  | **State** | **Contrast in probability** | **Lower 95%CI** | **Upper 95%CI** |  |
| --- | --- | --- | --- | --- | --- | --- |
| **Gender (Male vs female reference)** |  | Healthy | 0.06 | 0.04 | 0.08 |  |
|  |  | INT | -0.04 | -0.06 | -0.03 |  |
|  |  | CMD | 0.03 | 0.01 | 0.05 |  |
|  |  | INT -> CMD | -0.04 | -0.05 | -0.02 |  |
|  |  | CMD -> INT | -0.04 | -0.06 | -0.03 |  |
|  |  | CVR | 0.04 | 0.03 | 0.05 |  |
|  |  | Death | 0.00 | 0.00 | 0.00 |  |
| **Ethnicity (Pakistani vs Bangladeshi reference)** |  | Healthy | 0.08 | 0.06 | 0.09 |  |
|  |  | INT | 0.01 | 0.00 | 0.03 |  |
|  |  | CMD | -0.06 | -0.08 | -0.04 |  |
|  |  | INT -> CMD | -0.01 | -0.02 | 0.00 |  |
|  |  | CMD -> INT | -0.02 | -0.03 | -0.01 |  |
|  |  | CVR | 0.00 | -0.01 | 0.00 |  |
|  |  | Death | 0.00 | 0.00 | 0.00 |  |
| **IMD (3+ vs 1 reference)** |  | Healthy | 0.07 | 0.05 | 0.09 |  |
|  |  | INT | -0.01 | -0.04 | 0.00 |  |
|  |  | CMD | -0.01 | -0.03 | 0.01 |  |
|  |  | INT -> CMD | -0.02 | -0.03 | 0.00 |  |
|  |  | CMD -> INT | -0.02 | -0.04 | -0.01 |  |
|  |  | CVR | 0.00 | -0.01 | 0.00 |  |
|  |  | Death | 0.00 | 0.00 | 0.01 |  |
| **Smoking (ever vs never reference)** |  | Healthy | -0.04 | -0.05 | -0.02 |  |
|  |  | INT | 0.02 | 0.00 | 0.03 |  |
|  |  | CMD | -0.04 | -0.06 | -0.01 |  |
|  |  | INT -> CMD | 0.02 | 0.01 | 0.03 |  |
|  |  | CMD -> INT | 0.02 | 0.01 | 0.03 |  |
|  |  | CVR | 0.01 | 0.00 | 0.02 |  |
|  |  | Death | 0.00 | 0.00 | 0.00 |  |
| **ICMM PRS (+2SD vs average [0])** |  | Healthy | -0.07 | -0.08 | -0.06 |  |
|  |  | INT | -0.02 | -0.03 | 0.00 |  |
|  |  | CMD | 0.04 | 0.02 | 0.06 |  |
|  |  | INT -> CMD | 0.01 | -0.01 | 0.02 |  |
|  |  | CMD -> INT | 0.03 | 0.01 | 0.04 |  |
|  |  | CVR | 0.01 | 0.00 | 0.01 |  |
|  |  | Death | 0.00 | 0.00 | 0.01 |  |
| Contrasts between the female reference covariate pattern [Bangladeshi participant living in IMD quintile 1 (most deprived) with ICM-MMPRS z-score of 0 (‘average risk’), and a ‘never’ smoker age 40 at the start of follow-up], and the contrast of interest. 95% Confidence intervals [Cis] generated by bootstrapping | | | | | |  |
|  |  |  |  |  |  |  |
