# Supplementary material for: Genetic and sociodemographic factors associated with trajectories of physical and mental health multimorbidity in a South Asian cohort in the UK: A multistate modelling analysis
Source: PLoS Med. 2026 Jul 9;23(7):e1004844. doi: 10.1371/journal.pmed.1004844 (PMC13349187; doi:10.1371/journal.pmed.1004844)

### Fig A: Probabilities of CVE following ICM-MM over a ten-year window (men)

##
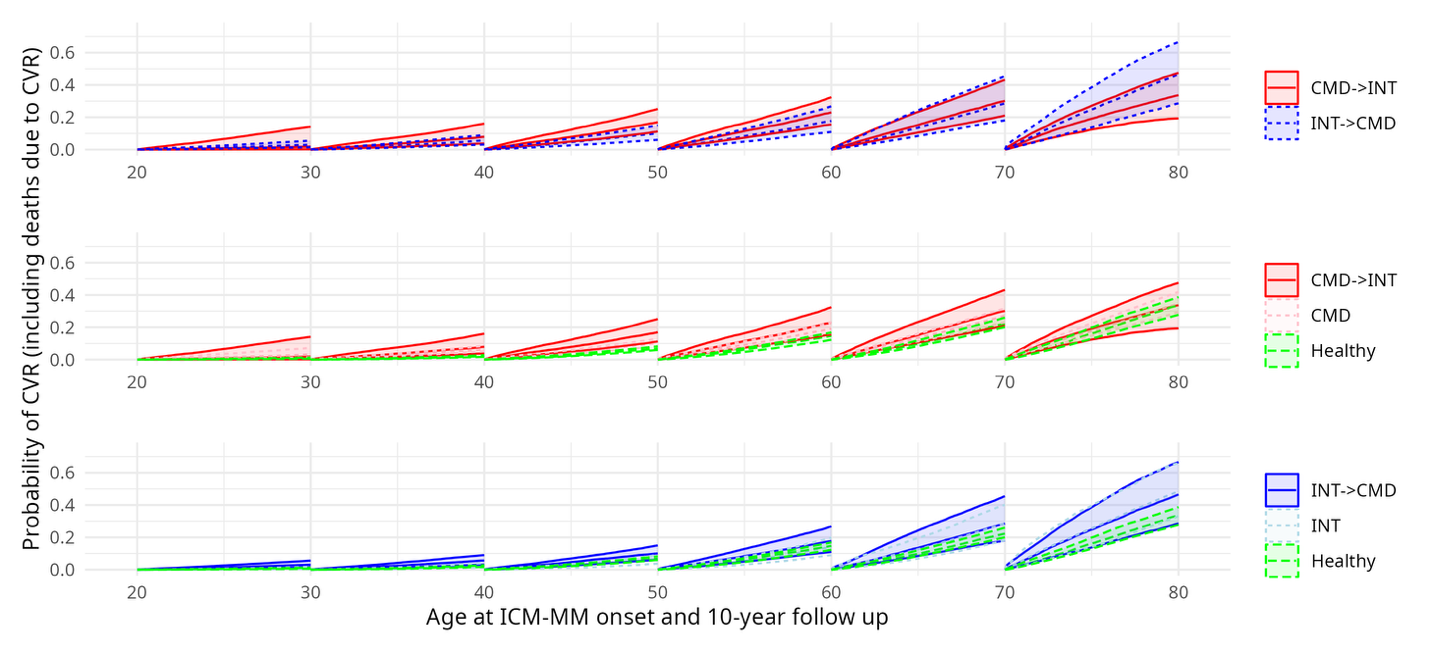


### Fig B: Probabilities of CVE following ICM-MM over a ten-year window (women)


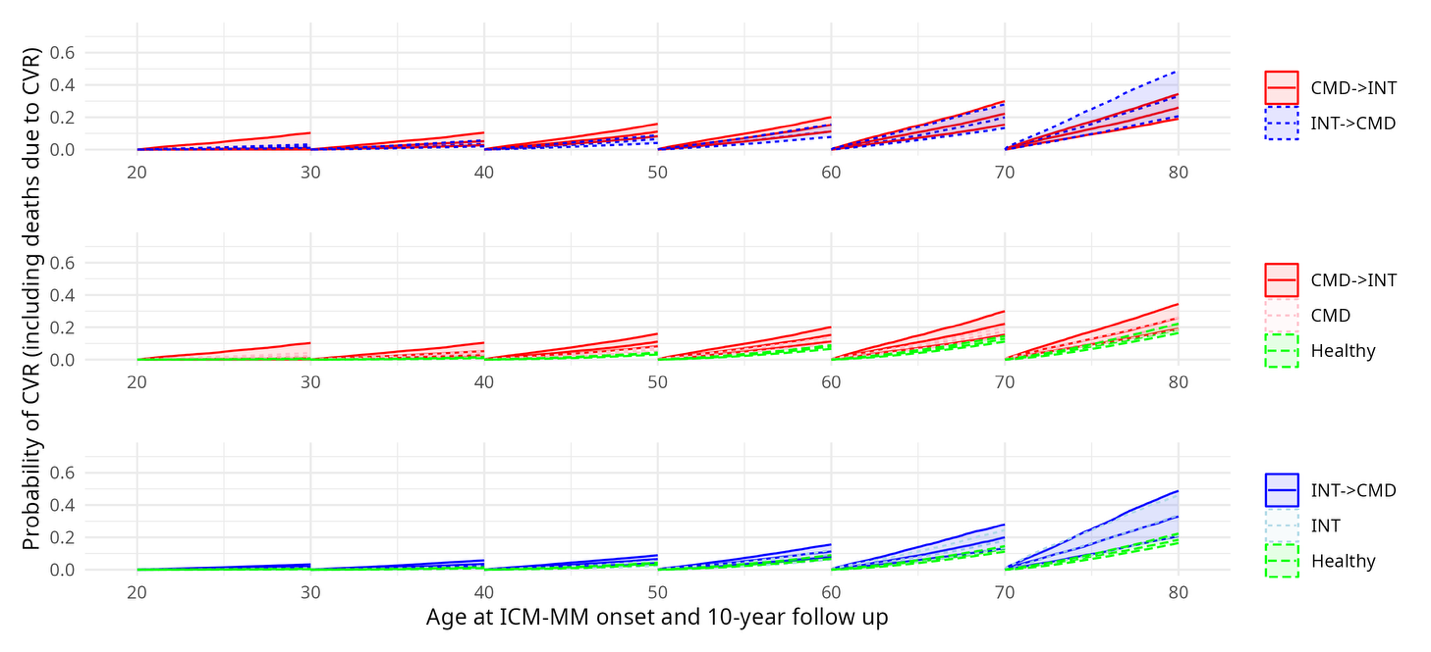


### Fig C: Contrast in probability of CVE following CMD→INT vs INT→ CMD(reference) ten years after ICM-MM onset (men)


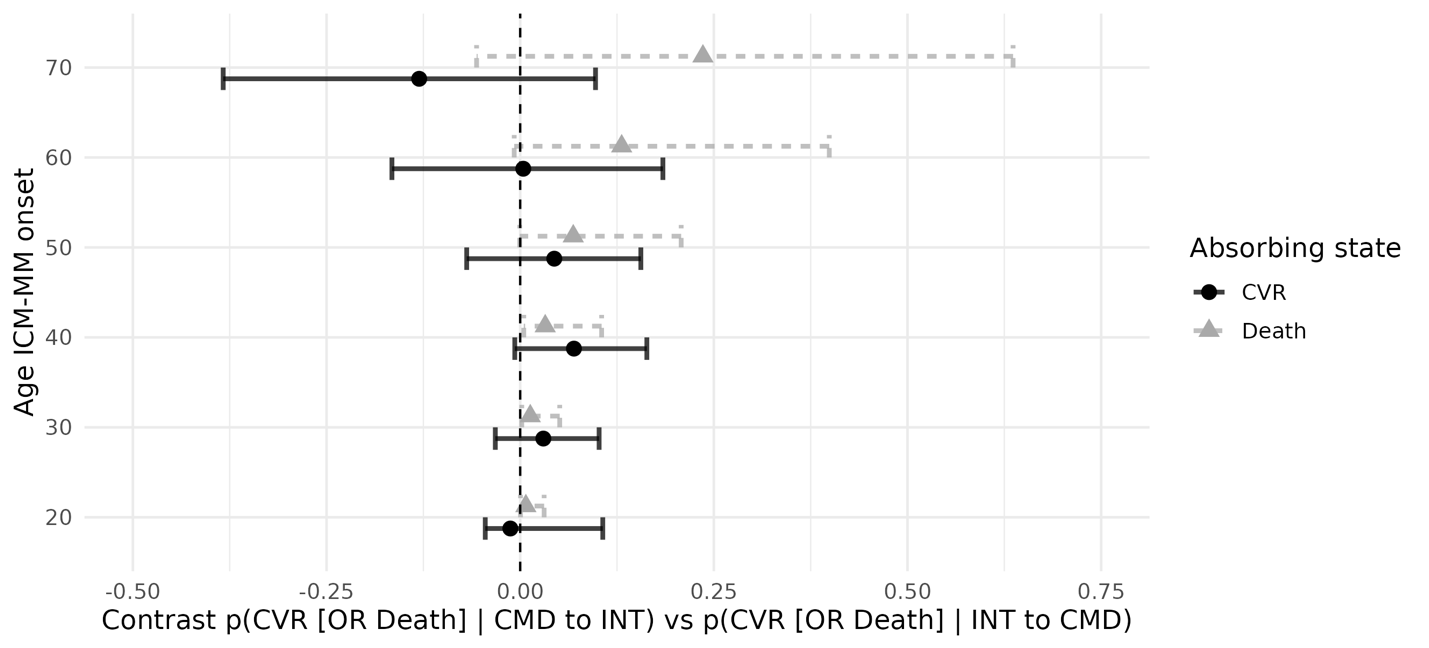


### Fig D: Contrast in probability of CVE following CMD→INT vs INT→ CMD(reference) ten years after ICM-MM onset (women)


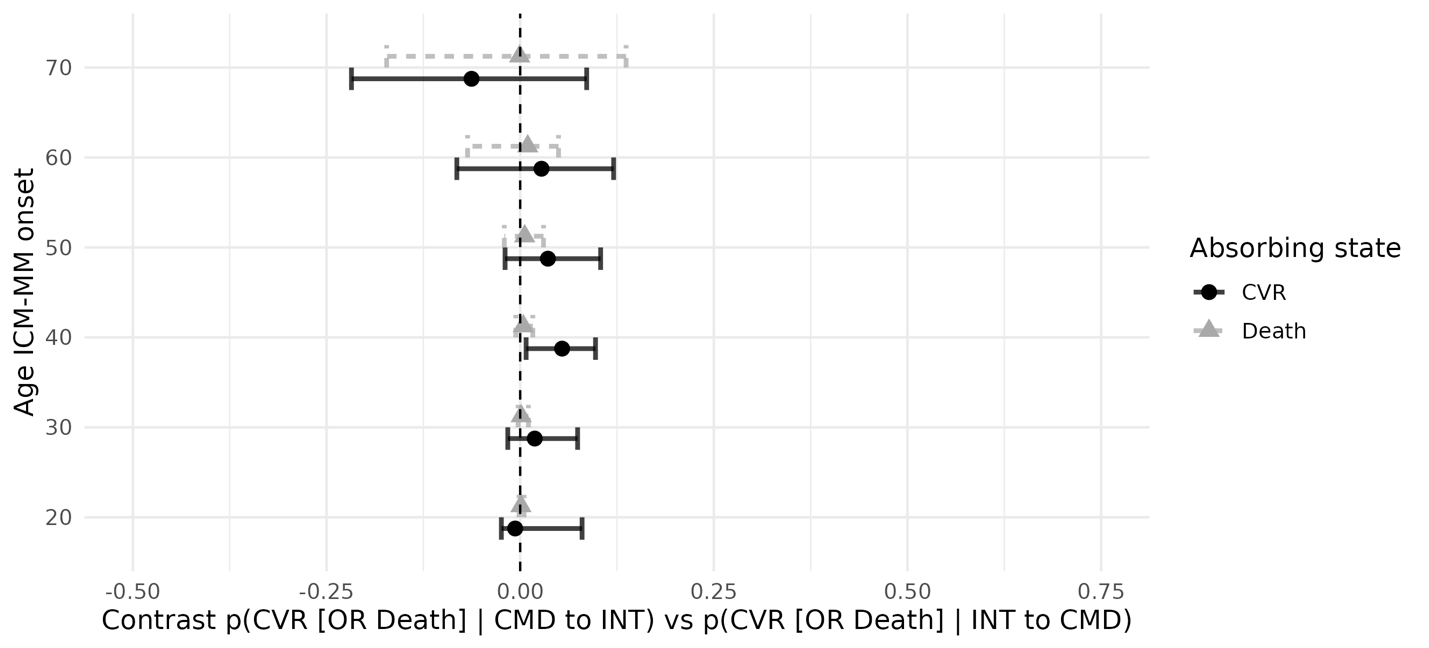

Supplement: S4 Text — Fig A: Probabilities of CVE following ICM-MM over a 10-year window (men). Fig B: Probabilities of CVE following ICM-MM over a 10-year window (women). Fig C: Contrast in probability of CVE following CMD → INT vs INT → CMD(reference) 10 years after ICM-MM onset (men). Fig D: Contrast in probability of CVE following CMD → INT vs INT → CMD(reference) 10 years after ICM-MM onset (women). (DOCX) [file pmed.1004844.s004.docx]
