# Supplementary material for: Genetic and sociodemographic factors associated with trajectories of physical and mental health multimorbidity in a South Asian cohort in the UK: A multistate modelling analysis
Source: PLoS Med. 2026 Jul 9;23(7):e1004844. doi: 10.1371/journal.pmed.1004844 (PMC13349187; doi:10.1371/journal.pmed.1004844)
Supplement: S1 Checklist — (DOCX) [file pmed.1004844.s005.docx]

STROBE Statement—Checklist of items that should be included in reports of ***cohort studies***

|  | Item No | Recommendation | Page No |
| --- | --- | --- | --- |
| **Title and abstract** | 1 | (*a*) Indicate the study’s design with a commonly used term in the title or the abstract | Page 1, line 1 title.  Page 4, line 41 onwards abstract |
|  |  | (*b*) Provide in the abstract an informative and balanced summary of what was done and what was found |  |
| Introduction | | | |
| Background/rationale | 2 | Explain the scientific background and rationale for the investigation being reported | Page 4-5, line 144-160, rationale. |
| Objectives | 3 | State specific objectives, including any prespecified hypotheses | Page 5 lines 173-185, objectives |
| Methods | | | |
| Study design | 4 | Present key elements of study design early in the paper | Page 6, line 187: methods |
| Setting | 5 | Describe the setting, locations, and relevant dates, including periods of recruitment, exposure, follow-up, and data collection | Page 6 line 198 setting, page 7 line 231 cohort design including dates |
| Participants | 6 | (*a*) Give the eligibility criteria, and the sources and methods of selection of participants. Describe methods of follow-up | Page 7 line 245 – participant eligibility |
|  |  | (*b*) For matched studies, give matching criteria and number of exposed and unexposed |  |
| Variables | 7 | Clearly define all outcomes, exposures, predictors, potential confounders, and effect modifiers. Give diagnostic criteria, if applicable | Page 8 line 257 onwards outcome measures, line 276 onwards exposure measures |
| Data sources/ measurement | 8* | For each variable of interest, give sources of data and details of methods of assessment (measurement). Describe comparability of assessment methods if there is more than one group | Page 6 , line 210 data sources |
| Bias | 9 | Describe any efforts to address potential sources of bias | Page 7, line 231 onwards, cohort design |
| Study size | 10 | Explain how the study size was arrived at | Page 12, figure 2 eligibility criteria flowchart |
| Quantitative variables | 11 | Explain how quantitative variables were handled in the analyses. If applicable, describe which groupings were chosen and why | Page 8, line 267 onwards, variable description |
| Statistical methods | 12 | (*a*) Describe all statistical methods, including those used to control for confounding | Page 9 line 303 onwards, statistical analysis., p10, line 334 complete case analysis, Supplemental table 1 contains information about excluded participants. Page 7, line 240 loss to followup |
|  |  | (*b*) Describe any methods used to examine subgroups and interactions |  |
|  |  | (*c*) Explain how missing data were addressed |  |
|  |  | (*d*) If applicable, explain how loss to follow-up was addressed |  |
|  |  | (*e*) Describe any sensitivity analyses |  |
| Results | | |  |
| Participants | 13* | (a) Report numbers of individuals at each stage of study—eg numbers potentially eligible, examined for eligibility, confirmed eligible, included in the study, completing follow-up, and analysed | Page 12, figure 2 |
|  |  | (b) Give reasons for non-participation at each stage |  |
|  |  | (c) Consider use of a flow diagram |  |
| Descriptive data | 14* | (a) Give characteristics of study participants (eg demographic, clinical, social) and information on exposures and potential confounders | Page 13 table 1, supplemental table 1 (missing data) |
|  |  | (b) Indicate number of participants with missing data for each variable of interest | Page 13 table 1 – follow up time |
|  |  | (c) Summarise follow-up time (eg, average and total amount) |  |
| Outcome data | 15* | Report numbers of outcome events or summary measures over time | Page 14, table 2 – health events during follow up |

| Main results | 16 | (*a*) Give unadjusted estimates and, if applicable, confounder-adjusted estimates and their precision (eg, 95% confidence interval). Make clear which confounders were adjusted for and why they were included | Figs 3a 3b – state occupation probabilties |
| --- | --- | --- | --- |
|  |  | (*b*) Report category boundaries when continuous variables were categorized | Figs 4 – 8 state occupation probabilities to contrast covariate patterns |
|  |  | (*c*) If relevant, consider translating estimates of relative risk into absolute risk for a meaningful time period |  |
| Other analyses | 17 | Report other analyses done—eg analyses of subgroups and interactions, and sensitivity analyses | NA |
| Discussion | | | |
| Key results | 18 | Summarise key results with reference to study objectives | Page 20, line 523 - 523 |
| Limitations | 19 | Discuss limitations of the study, taking into account sources of potential bias or imprecision. Discuss both direction and magnitude of any potential bias | Page 22, line 606 onwards |
| Interpretation | 20 | Give a cautious overall interpretation of results considering objectives, limitations, multiplicity of analyses, results from similar studies, and other relevant evidence | Page 23, line 660 onwards |
| Generalisability | 21 | Discuss the generalisability (external validity) of the study results | See limitations, also in page2 abstract and page 3, author summary |
| Other information | | | |
| Funding | 22 | Give the source of funding and the role of the funders for the present study and, if applicable, for the original study on which the present article is based | Page 24 line 690 Funding |

*Give information separately for exposed and unexposed groups.

**Note:** An Explanation and Elaboration article discusses each checklist item and gives methodological background and published examples of transparent reporting. The STROBE checklist is best used in conjunction with this article (freely available on the Web sites of PLoS Medicine at http://www.plosmedicine.org/, Annals of Internal Medicine at http://www.annals.org/, and Epidemiology at http://www.epidem.com/). Information on the STROBE Initiative is available at http://www.strobe-statement.org.
