# Supplementary material for: Genetic and sociodemographic factors associated with trajectories of physical and mental health multimorbidity in a South Asian cohort in the UK: A multistate modelling analysis
Source: PLoS Med. 2026 Jul 9;23(7):e1004844. doi: 10.1371/journal.pmed.1004844 (PMC13349187; doi:10.1371/journal.pmed.1004844)
Supplement: Attachment — Submitted filename: paper review PLOS medicine.docx [file pmed.1004844.s007.docx]

**General Comments**

The manuscript addresses an important topic, I think overall the methods used are appropriate. However, several areas need clarification and refinement to strengthen its contribution. Below are detailed comments organised by section. I think the paper needs to be clearer on what is novel about this research – for example are the results different for the ethnic populations considered in the paper compared with the rest of the UK? What are the impacts of this work for policy and practice?

**Introduction**

- Please clarify that multimorbidity is also referred to as *Multiple Long-Term Conditions (MLTCs)*, as this term is becoming increasingly comment in multimorbidity research.
- Consider introducing multimorbidity within the UK context, as the paper focuses on UK data.
- It would also be helpful to quantify the impact of multimorbidity on the health service.

**Methods**

- Why focus only on cardiometabolic conditions? Multimorbidity typically includes a broader range of conditions. Given this restriction, can the paper claim to examine multimorbidity, or is it more accurately mental health and cardiometabolic comorbidity?
- Patient and public involvement is commendable, but more detail is needed—how many participants, and what influence did they have?
- You state that *Genes & Health* was approved by the National Research Ethics Committee, but no reference is provided.
- Consider starting this section with Data sources and setting; move ethics information after that.
- There is a random “)” in the Data sources and setting section.
- Clarify how data access was granted for confidential data—e.g., was this through secure servers?
- The statement “no record of any internalising or cardiometabolic conditions before start of follow-up” does not account for a lag period. Similar studies often use a condition-free period (e.g., 5 years), why was this not used in this study?
- For demographic variables, please include categories for gender and ethnicity. Also, clarify whether you report gender or biological sex—they are distinct measures.
- I understand why you collapsed IMD categories 3, 4, and 5 into a single ‘least deprived’ group; however, this should be discussed in the Discussion or Limitations section. Socioeconomic factors are important for health, and by collapsing these categories you have reduced a lot of the granularity of this measure.
- Why include smoking status but not other cardiovascular risk factors such as diet, obesity, exercise, or alcohol consumption?
- Under genetic risk, there is a placeholder “[Additional ref]”—please correct.
- Expand on why pulse pressure is used as a proxy for hypertension, as this seems unusual.
- Clarify whether BMI is used as a proxy for obesity or calculated using the standard BMI formula.
- What happens if someone reports two states simultaneously? How was this handled?
- You should really define what you mean by internalising conditions (particular in the abstract) some readers may be unaware of what this includes.
- The authors used complete case analysis for missing data. Please clarify the proportion of participants excluded due to missingness and discuss potential bias introduced by this approach. Consider whether multiple imputation or sensitivity analyses would strengthen the findings.
- The paper states that it examined analysis by ethnicity, but this was limited to a comparison between British Pakistani and British Bangladeshi groups. This should be made explicit, as the results do not reflect a broader analysis across multiple ethnicities.

**Results**

- Some findings may lack novelty:
  - Younger age and internalising conditions are expected.
  - Declining probability of remaining healthy over time, especially in older groups, is well established.
- The differences between British Pakistani and British Bangladeshi are central to the paper—draw these out more clearly.
- It would have been good to compare British Pakistani and Bangladeshi participants with British White ethnicity for more striking contrasts. Although I assume data is not available to do this, it could however still be mentioned in the discussions.
- Throughout the results – particularly the figures it would be good to present the sample size of the models

**Discussion**

- **Limitations**:
  - The multistate trajectory does not allow return to previous states—important for internalising conditions that may fluctuate over time.
  - Summarise limitations of electronic health records relevant to this study.
  - Missing covariates that could improve your modelling should be discussed in the limitations.
- There is a lot more research that considers multimorbidity complexity (beyond simple counts of conditions), this should be mentioned.
- Given you talk about the UK Pakistani and Bangladeshi populations as a whole it would be good to reflect briefly on how representative the Genes & Health participants are to UK population.
- I think it would be helpful to discuss a bit more what these findings mean for policy and practice.
- I think it would be useful to discuss findings in relation to other ethnic groups, what stands out about the UK Pakistani and Bangladeshi populations particularly compared to other ethnic groups.

**Minor Points**

- Review acronyms—are they all necessary?
- Be consistent with decimal places in reported results.
- You refer to your sample being both Pakistani South Asian and British Pakistani – these seem slightly different to me and it would be good to clarify and use consistently throughout the paper
