# Supplementary material for: Genetic and sociodemographic factors associated with trajectories of physical and mental health multimorbidity in a South Asian cohort in the UK: A multistate modelling analysis
Source: PLoS Med. 2026 Jul 9;23(7):e1004844. doi: 10.1371/journal.pmed.1004844 (PMC13349187; doi:10.1371/journal.pmed.1004844)
Supplement: Attachment — Submitted filename: 2026-03-27_REVIEW_RESPONSE.docx [file pmed.1004844.s009.docx]

We would like to thank all of the reviewers for the time spent reading and considering our article, and for the positive comments acknowledging the strengths of our study. We are very grateful for the constructive, insightful comments and suggestions: we have thoroughly revised the article to address these, and hope the reviewers find the latest version an improvement on the previous submission.

Daniel Stow, on behalf of all co-authors

| **Reviewer #1:** |  |
| --- | --- |
| The study by Stow et al. represents a timely and novel contribution to the literature on multimorbidity, particularly at the interface of internalising mental health conditions, cardiometabolic disease, and genetic risk in under-represented British Bangladeshi and Pakistani populations. The manuscript is generally well-written, the statistical analysis is appropriate and carefully executed, and the authors have thoughtfully addressed key limitations. However, I have some comments/concerns regarding the definition of the primary endpoint and the need for additional detail and clarification in the presentation of the results and figures, which I believe would further improve the clarity and interpretability of the paper |  |
| **Major comments:** |  |
| 1) Regarding the definition of the primary endpoint, the authors have defined a composite "cardiovascular event (CVE)" endpoint that includes end-stage renal disease (ESRD). ESRD is conventionally regarded as a renal outcome rather than a cardiovascular one. Including ESRD within a composite labelled as purely "cardiovascular" introduces a conceptual inconsistency between the name of the endpoint and its components and may be incorrect. I recommend either: (i) removing ESRD from the primary CVE composite and analysing renal outcomes separately as a pre-specified secondary endpoint, or (ii) renaming the composite to reflect its broader scope (e.g. "major cardiovascular or renal event (CVR event)") and explicitly justifying the inclusion of ESRD as part of a combined cardio-renal endpoint. | We agree that this labelling was unclear, and have updated the article throughout to clarify that our primary endpoint is “major CardioVascular and Renal event” (CVR) – thank you very much for this suggestion.  The selection of conditions that make up our CVR endpoint was arrived at in collaboration with clinical members of the LINC project, and patients and members of the public who wanted to understand how sociodemographic and genetic factors modify risk of ICM-MM and related future health events. End stage renal disease was added to our study endpoint because it reflects a serious downstream consequence of some of the conditions in the ICM-MM definition: chronic kidney disease especially. |
| 2) Please add a supplementary table reporting, for each age category, the number of patients and the number (and percentage) of events at the end of follow-up. This should correspond to the number (percentage) of events shown in Figure 3a and Figure 3b. | We have added supplemental table 3 – referred to under ‘sequence and number of events’  Please see supplemental table 3:  *“Proportion of participants with internalising and cardiometabolic conditions, ICM-MM, CVR and all-cause mortality by age group..”* |
| 3) Please add a supplementary table reporting the frequency of each internalising mental health condition and each cardiometabolic condition in the study population. | We have added supplemental table 2 – referred to under ‘results’  Please see supplemental table 2:  *“Frequency of internalising and cardiometabolic conditions, and cardiovascular / renal events in the analytic sample”* |
| 3b) I also suggest adding a brief section to the Results and Discussion describing which specific conditions were most prevalent within each group, and commenting on how this pattern of prevalence may influence the interpretation of the multimorbidity trajectories. | We have added to the results section to highlight the most common index internalising and cardiometabolic conditions:  *“of which anxiety and phobia was the most common index condition (n=2,620 [11.**1%] – Supplemental table 2)”*  *“of which dyslipidaemia was the most common index condition (n=3,091 [13.1%]] – Supplemental table 2);”*  We have also added to the discussion  *“This earlier onset cardiometabolic profile with higher internalising condition risk (and subsequent risk of cardiovascular event) should be a target for early screening and intervention.* *Lipid profiles are one of the current screening measures used in the NHS health check(50) and dyslipidaemia was the most common index cardiometabolic condition in our study. This builds on other recent studies in UK biobank that found lipid profiles are an important precursor to cardiometabolic multimorbidity.(60) “* |
| **Minor comments:** |  |
| 4) In the Methods-Genetic Risk section, the references to the previous study/studies that described the methods used to derive the novel PRS for ICM-MM are missing. | We apologise for this error: the reference should have been to a medRxiv preprint detailing the development of the ICM-MM PRS – this is now *in press* with Human Molecular Genetics, and we have updated the reference accordingly. |
| 5) Some abbreviations are spelled out in full and then repeated inconsistently in different sections of the manuscript. I suggest revising the entire manuscript to ensure that all abbreviations are introduced and used consistently, in line with the journal's guidelines. | We have added a list of acronyms to the end of the manuscript and attempted to tidy up / be more consistent with acronym usage throughout |
| 6) In Table 1, there appears to be an error in the column heading for the Pakistani female group (it is incorrectly labelled as male). | Thank you for spotting this - please see revised table 1 |
| 6b) In addition, please report all numerical values with a consistent number of decimal places (e.g. 1 or 2 decimals, according to the journal's guidelines). For example, some numbers for the ICMM PGS and follow-up duration are currently reported with up to 5 decimal places. | Thank you for spotting this too – please see revised table 1 |
| 7) For Figure 1, I recommend adding the definitions of the internalising mental health conditions and cardiometabolic conditions to the figure legend, to help readers fully understand how the model works and what the target multimorbidity cluster comprises. | We have revised the captions for this figure to clarify the conditions included in each of the states.  *“**Fig 1: The seven-state, 14 transition multi state model used to define health transitions. Internalising conditions are anxiety, depression, and somatoform disorders; cardiometabolic conditions are hypertension, type 2 diabetes, obesity, dyslipidaemia, chronic kidney disease. CVR is occurrence of or death due to: atrial fibrillation and flutter, heart failure, coronary heart disease, peripheral vascular disease, cerebrovascular disease (TIA/stroke), and end stage renal disease. Death is all non-CVR mortality. “* |
| 8) Figure 3a and Figure 3b are very important and informative. However, with seven classes (six blue coloured states), it is difficult for readers to distinguish between the "multimorbid (INT→CMD)", "multimorbid (CMD→INT)", and "cardiovascular event" states. I highly recommend using different colours/shades to increase visual separation between these states. | We have updated these figures to use a different colour pallette:  Please see revised figures 3a and 3b   |
| 9) For Figure 6: I suggest adding the full term "Index of Multiple Deprivation (IMD)" in the Figure 6 legend, and using "Index of Multiple Deprivation contrast" instead of "IMD contrast" in the figure title, to aid reader understanding, particularly for those unfamiliar with the abbreviation. | Thank you for this recommendation – we have revised this figure caption to make the abbreviation clearer.  “State occupation probability contrasts for highest Index of Multiple deprivation (IMD) tertile (3+) versus reference (IMD=1)” |

| **Reviewer 2:** |  |
| --- | --- |
| The manuscript addresses an important topic, I think overall the methods used are appropriate. However, several areas need clarification and refinement to strengthen its contribution. Below are detailed comments organised by section. I think the paper needs to be clearer on what is novel about this research – for example are the results different for the ethnic populations considered in the paper compared with the rest of the UK? What are the impacts of this work for policy and practice? |  |
| **Introduction** |  |
| Please clarify that multimorbidity is also referred to as *Multiple Long-Term Conditions (MLTCs)*, as this term is becoming increasingly common in multimorbidity research. | We have added to the introduction:  *“Over a third of the world’s population live with multimorbidity,(1) often referred to as multiple long-term conditions, and defined as the co-occurrence(2) or lifetime occurrence(3) of two or more long-term health conditions”* |
| Consider introducing multimorbidity within the UK context, as the paper focuses on UK data. | Thank you for this recommendation - we have extended the introduction and clarified that the studies mentioned in the introduction are UK based  *“**Population based studies in the United Kingdom (UK) have identified patterns of frequently co-occurring long-term conditions (sometimes referred to as multimorbidity clusters), highlighting some of the most prevalent combinations, including hypertension, type 2 diabetes, dyslipidemia, obesity, depression, and anxiety.(7) UK based studies have also highlighted the impact of multimorbidity on health service utilisation and associated costs (including increased frequency of primary and secondary healthcare contacts, and number of prescribed medications).(8, 9)”* |
| It would also be helpful to quantify the impact of multimorbidity on the health service. | We have expanded on the impacts on health service use in the introduction:  *“impact on health service utilisation and cost (including increased frequency of primary and secondary healthcare contacts, and number of prescribed medications).”* |
| **Methods** |  |
| Why focus only on cardiometabolic conditions? Multimorbidity typically includes a broader range of conditions. Given this restriction, can the paper claim to examine multimorbidity, or is it more accurately mental health and cardiometabolic comorbidity? | The LINC collaborative was formed and funded to investigate a specific form of internalising and cardiometabolic multimorbidity. We have updated the introduction to highlight that many different operationalisations of multimorbidity have been used in multimorbidity research to date, all falling under the canonical definition of ‘two or more long term conditions’. We have also clarified that ours is a collaborative-specific multimorbidity phenotype as early as possible, to complement the rationale contained in the S1 text.  *“Many operationalisations of multimorbidity have been used in existing research.(2, 4) Here, we use a collaborative-specific operationalisation: the lifetime co-occurrence of internalising (depression, anxiety, somatoform) and cardiometabolic conditions (hypertension, obesity, type 2 diabetes) to characterise physical-mental health multimorbidity health trajectories in a large UK sample of British Bangladeshi and Pakistani individuals…”* |
| Patient and public involvement is commendable, but more detail is needed—how many participants, and what influence did they have? | Thank you for this recommendation, we have updated the PPI section to provide greater detail on the number of people involved, and the specific, important, contributions made, as well as acknowledging key members in the body of the text.  *“**Nine members of the public with lived experiences of multiple long-term conditions worked with the LINC study, including Jane Sprackman and Shahid Khan who were also PPI co-applicants on LINC and represented the wider PPI group at study management meetings. PPI partners worked in collaboration with primary and secondary care doctors and other members of the research team to define the study specific multimorbidity operationalisation. They also contributed to the design of this analysis - highlighting priority areas and informing the research questions, including the focus on the impact of condition ordering on future health events related to multimorbidity.”* |
| You state that *Genes & Health* was approved by the National Research Ethics Committee, but no reference is provided. | We apologise that this section was not worded clearly: we have clarified that  *“Genes & Health was approved by the National Research Ethics Committee - London and Southeast (reference 13/LO/124).”* |
| Consider starting this section with Data sources and setting; move ethics information after that. | We have relocated the ethics section below the data sources and setting section. |
| There is a random “)” in the Data sources and setting section. | Thank you for spotting this – now removed |
| Clarify how data access was granted for confidential data—e.g., was this through secure servers? | We have clarified that access to data is via an ISO27001 compliant trusted research environment.  *“Genes & Health hosts data for researchers in a secure ISO27001 certified Trusted Research Environment. The Trusted Research Environment is currently hosted by Google Cloud Platform in London. The Data Controller is Queen Mary University of London.”* |
| The statement “no record of any internalising or cardiometabolic conditions before start of follow-up” does not account for a lag period. Similar studies often use a condition-free period (e.g., 5 years), why was this not used in this study? | Thank you for this suggestion. We have revised the cohort specification to include a six month washout period following cohort entry, including a reference to Lewis et al., (our ref 34) which suggests 3 months as a suitable minimum to avoid prevalence/incidence misclassification.  All results and figures have been revised throughout to reflect the updated cohort design.  *“Start of follow-up was defined as the cohort entry date, plus a six-month lag (or ‘washout’) period, to exclude prevalent or pre-existing conditions from the incidence risk set.(34, 35) “*  *“Eligible participants were age ≥16 years old, with ≥1 record in G&H primary care data, no record of any internalising or cardiometabolic conditions before start of follow-up, and total follow-up time >0 days. “* |
| For demographic variables, please include categories for gender and ethnicity. Also, clarify whether you report gender or biological sex—they are distinct measures. | Responses were taken from self- report questionnaires, and we refer to gender throughout. We have clarified the demographic variables section:  *“Participants’ self-reported gender, and ethnicity (Bangladeshi or Pakistani) were taken from the G&H demographic questionnaire. Participants responding ‘other’ to the ethnicity item were excluded from this analysis.”* |
| I understand why you collapsed IMD categories 3, 4, and 5 into a single ‘least deprived’ group; however, this should be discussed in the Discussion or Limitations section. Socioeconomic factors are important for health, and by collapsing these categories you have reduced a lot of the granularity of this measure. | Further to this and comments from other reviewers, we have included supplemental table 1 – this shows the distribution of IMD in our analytic sample, and in the G&H sample in East London, which is broadly representative of the background population as described in the Genes & Health cohort profile paper (Finer et al., 2020). We highlight that the majority of participants in the background population live in areas characterised by high deprivation (IMD 1-2)  **Setting**:  *“The convenience sample is broadly representative of the background population in East London – with some over-representation of women, and those aged <45 years.(28) The majority of G&H volunteers in East London live in areas characterised by high levels of deprivation (supplemental table 1).”*  We have also added a note to the limitation section:  “Due to the very low numbers of people living in IMD quintiles four and five in this population, we collapsed these categories, further reducing the granularity of this measure.” |
| Why include smoking status but not other cardiovascular risk factors such as diet, obesity, exercise, or alcohol consumption? | We have expanded our limitations section on EHR data to clarify that factors such as diet / exercise and alcohol are not well captured in EHR data in the UK (whereas smoking behaviours are relatively well captured).  *“Our study uses routinely collected electronic healthcare records, the limitations of which are well reported (see Farmer et al for an example).(66) One key limitation is the lack of information on individual-level risk factors associated with cardiometabolic and internalising conditions. With the notable exception of smoking(67) many of these factors, such as alcohol consumption(68) and exercise,(69) are poorly captured in EHR data “* |
| Under genetic risk, there is a placeholder “[Additional ref]”—please correct | We apologise for this error: the reference should have been to a medRxiv preprint for the development of the ICM-MM prs – this is now *in press* with Human Molecular Genetics, and we have updated the reference accordingly. |
| Expand on why pulse pressure is used as a proxy for hypertension, as this seems unusual. | We have revised the section on the development of the PRS for ICM-MM – summary statistics excluding UK Biobank were used: results for pulse pressure were available for a large (non UKB sample) and a PRS based on these was used as one of the inputs to the elastic net regression. |
| Clarify whether BMI is used as a proxy for obesity or calculated using the standard BMI formula. | Our ICM-MM definition includes diagnosis of obesity as one of the long-term conditions. We have clarified that diagnostic codes were used (as opposed to calculating BMI)  Methods  “Age of onset for each condition was defined as the first occurrence of any relevant diagnostic code in primary care or HES records. “  We have also acknowledged our use of diagnosed obesity as a potential limitation in our discussion:  “Using diagnosed obesity as part of the cardiometabolic condition definition also removed our ability to investigate BMI as a potential moderator on the proposed causal relationship between depression and diabetes.(71) “ |
| What happens if someone reports two states simultaneously? How was this handled? | We apologise that this was not clear in the methods section: as with previous studies using multi state models (Owen et al., Lancet Public Health 2023), individuals with health events recorded on the same date were excluded from the analysis – this was in figure 2 but the accompanying text in methods was missing. We have clarified that:  [Methods]  *“Individuals with internalising, cardiometabolic, CVR or death events recorded on the same day do not inform transitions in the multi state model, and these participants (n=56) were excluded from the study”* |
| You should really define what you mean by internalising conditions (particular in the abstract) some readers may be unaware of what this includes. | We have clarified the abstract:  ***“****internalising mental health condition (depression, anxiety, somatoform disorders)”*  And in the introduction  *“Here, we use a collaborative-specific operationalisation: the lifetime co-occurrence of internalising (depression, anxiety, somatoform)”*  And  *“In this study, we focus on a cluster of physical and mental health multimorbidity comprising internalising (as opposed to externalising)(22)”* |
| The authors used complete case analysis for missing data. Please clarify the proportion of participants excluded due to missingness and discuss potential bias introduced by this approach. Consider whether multiple imputation or sensitivity analyses would strengthen the findings. | We have clarified figure 2 – the penultimate box shows the proportion of people excluded due to missing sociodemographic data (9.3%).  Fig2. Penultimate box, 4.5% excluded due to missing sociodemographic information  Supplemental table 1 shows the proportion of missingness for key sociodemographic variables and highlights that smoking status is the most commonly missing variable (missing in 5.7% of the 22,002 excluded participants) |
| The paper states that it examined analysis by ethnicity, but this was limited to a comparison between British Pakistani and British Bangladeshi groups. This should be made explicit, as the results do not reflect a broader analysis across multiple ethnicities. | We have updated the final paragraph of the introduction to clarify that our investigation on the influence of ethnicity is for Bangladeshi and Pakistani ethnicities only  *“**We then investigated the influence of ethnicity (Bangladeshi and Pakistani), sociodemographic and genetic risk, using a novel polygenic risk score for multimorbidity, on progression through these trajectories.”* |
| **Results** |  |
| Some findings may lack novelty:   - Younger age and internalising conditions are expected. - Declining probability of remaining healthy over time, especially in older groups, is well established. | We apologise if the novelty of these findings is overemphasised: the findings you highlight here reflect a validation of established health patterns in epidemiological data: we are grateful for your suggestion below to highlight further the differences in health states between British Pakistani and British Bangladeshi participants. |
| The differences between British Pakistani and British Bangladeshi are central to the paper—draw these out more clearly. | Thank you for this recommendation – we agree this is an important finding and have revised our discussion to prioritise this, and highlight the availability of self-described ethnicity as a key strength of our study.  **Please see discussion paragraph starting**  *“A key strength of our study is the availability of self-reported information on ethnicity, meaning we can highlight important health inequalities within UK South Asian populations”* |
| It would have been good to compare British Pakistani and Bangladeshi participants with British White ethnicity for more striking contrasts. Although I assume data is not available to do this, it could however still be mentioned in the discussions. | We agree that this comparison would be very useful – we investigated a replication study in UK Biobank but primary care data are available for only ~100 Bangladeshi participants. We have added a recommendation for future research to the end of the discussion  *“Future work should also replicate our findings in larger cohorts, with genetic and healthcare data for a wider range of ancestral groups – this may be possible with the emerging availability of data from the ‘Our Future Health’ study.(79)”* |
| Throughout the results – particularly the figures it would be good to present the sample size of the models | We have updated the results section to show the number of observed health events (table 2) underlying each of the survival models, and have added the number of observations in each model to the modelling supplement. All plots in the main article use a combination of these survival models to calculate state occupation probabilities via simulation using 10,000 individuals |
| **Discussion** |  |
| There is a lot more research that considers multimorbidity complexity (beyond simple counts of conditions), this should be mentioned. | This is an important point – we have updated our discussion to highlight that multimorbidity research has progressed beyond condition counts (citing a review published after our initial submission [https://www.nature.com/articles/s41467-025-67372-6]), and use this to introduce our findings on condition ordering.  “Methodological advances mean that multimorbidity research has progressed beyond simple counts of conditions to account for complexity and clustering, but temporality is still under-studied.(52)” |
| Given you talk about the UK Pakistani and Bangladeshi populations as a whole it would be good to reflect briefly on how representative the Genes & Health participants are to UK population. | Following this comment, and comments from previous reviewers we have updated our methods section to comment on the G&H sample in East London – noting that it is broadly representative of the background population, referencing the cohort specification paper (Finer et al, 2020 – our ref 28)    *“The convenience sample is broadly representative of the background population in East London….(28)”*  We have also added to the discussion to suggest future work to investigate reweighting the sample to even more closely match the background population.  “The G&H sample in East London is broadly representative of the British Bangladeshi and British Pakistani population in East London, but over-represents slightly women and people age <45. Future work should target strategies to reweight the G&H sample to fully reflect the background population in terms of sociodemographic structure, and health.(73) |
| I think it would be helpful to discuss a bit more what these findings mean for policy and practice. | Thank you for this recommendation: we have substantially revised our discussion and clarified that earlier intervention and screening should be made available via the NHS health check in our conclusion.  **Conclusions**  *“The early onset of many of the ICM-MM conditions, and ICM-MM itself* *suggests earlier implementation of NHS health check”* |
| I think it would be useful to discuss findings in relation to other ethnic groups, what stands out about the UK Pakistani and Bangladeshi populations particularly compared to other ethnic groups. | We have revised the introduction to expand on UK Pakistani and Bangladeshi populations .  *“In the UK, South Asian populations are at the highest risk of early-onset multimorbidity, leading to greater health burden, and reduced life expectancy.(13) Pakistani and Bangladeshi populations specifically are also more likely than other South Asian (and European populations) to live with multiple long-term conditions and report poorer health.(14, 15)”*  And highlight the higher genetic risk of cardiometabolic conditions  *“The early emergence of multimorbidity in these populations, is supported by epidemiological and genomic literature demonstrating high genetic risk, and early onset of conditions such as type 2 diabetes,(16) which are a common component of most multimorbidity clusters.”*  We have also added recommendations for future work to investigate multimorbidty in more diverse ancestral and ethnic groups  *“Future work should also replicate our findings in larger cohorts, with genetic and healthcare data for diverse ancestral and ethnic groups – this may be possible with the emerging availability of data from the ‘Our Future Health’ study.”* |
| **Limitations** |  |
| The multistate trajectory does not allow return to previous states—important for internalising conditions that may fluctuate over time. | Thank you for highlighting this - we have updated the limitation section to reflect the implications of the assumption of chronicity of conditions on the multi state model.  *“This has implications for our multi-state framework as individuals could not re-enter a previous health state, which may not reflect the episodic nature of the internalising mental health conditions.”* |
| Summarise limitations of electronic health records relevant to this study. | We have updated our limitations section to clarify some of the specific limitations of EHRs relevant to our study  Please see:  *“**Our study uses routinely collected electronic healthcare records, the limitations of which are well reported (see Farmer et al for an example).(67) One key limitation is the lack of information on individual-level risk factors associated with cardiometabolic and internalising conditions. With the notable exception of smoking(68) many of these factors, such as alcohol consumption(69) and exercise,(70) are poorly captured in EHR data. “*  and:  *“In common with other studies that have used electronic healthcare records to define health phenotypes we have made a series of assumptions about ascertainment and chronicity of the conditions, as there is often no triggering contact that would make a clinician record remission/recovery. This has implications for our multi-state framework as individuals could not re-enter a previous health state, which may not reflect the episodic nature of the internalising mental health conditions.”* |
| Missing covariates that could improve your modelling should be discussed in the limitations. | Following your previous recommendation, we have highlighted information on individual level exposures that are not well captured in EHRs as one of the key limitations to the study. |
| **Minor Points** |  |
| Review acronyms—are they all necessary? | We have reviewed and revised our use of acronyms throughout – we have also added a list of acronyms at the end of the article |
| Be consistent with decimal places in reported results. | Thank you for highlighting this – we have updated tables and figures to be more consistent with decimal places. |
| You refer to your sample being both Pakistani South Asian and British Pakistani – these seem slightly different to me and it would be good to clarify and use consistently throughout the paper | Thank you for spotting this – ‘Pakistani South Asian’ was an error – we have removed this. |

| **Reviewer 3** |  |
| --- | --- |
| Reviewer #3: The manuscript aimed to examine life course of mental-physical multimorbidity among UK South Asian population between 1997 to 2024. Multi-state models were applied to explore trajectories of internalising and cardiometabolic multimorbidity and risk of cardiovascular event or death. The study observed that 14% of 25641 Bangladeshi and Pakistani participants developed the multimorbidity and 7% with cardiovascular events and 0.5% died. Women, younger age, people with higher deprivation, and people who smoked tend to report multimorbidity. The study focused on a prevalent and important health issue-co-existence of mental-physical multimorbidity. At the same time, sociocultural issues (racialized populations) are intertwined with the disease outcome. |  |
| **Major issues** |  |
| **Introduction** |  |
| The Introduction explained why decided to examine the disease outcome among UK South Asian population, but from the readers of the journal perspectives, a global literature review on the life course of multimorbidity is necessary. What has been discovered?  What are the remaining knowledge gaps? Challenges and opportunities to address the necessity of life course of multimorbidity? Why are UK South Asians being studied in this context? And others to elaborate on the contextual information around the study topic. | We have revised our introduction to reflect the UK specific data in line with recommendations from other reviewers – we have also updated the introduction to highlight a previous study that demonstrates the high burden of early onset multimorbidity in UK South Asian populations, and subsequent impact on years life lost, as well as highlighting the impact of high genetic risk for cardiometabolic conditions in South Asian populations  “In the UK, South Asian populations are at the highest risk of early-onset multimorbidity, leading to greater health burden, and reduced life expectancy.(13) Pakistani and Bangladeshi populations specifically are also more likely than other South Asian (and European populations) to live with multiple long-term conditions and report poorer health.(14, 15) The early emergence of multimorbidity in these populations, is supported by epidemiological and genomic literature demonstrating high genetic risk, and early onset of conditions such as type 2 diabetes,(16) which are a common component of most multimorbidity clusters.” |
| **Methods** |  |
| What is the coverage of G&H for UK South Asians? Similarly, what is the coverage of outcome measures for the studied populations? | Thank you for this recommendation: we have revised our methods and discussion substantially discuss the representativeness of the G&H sample  **Setting**  *“The convenience sample is broadly representative of the background population in East London – with some over-representation of women, and those aged <45 years.(28) The majority of G&H volunteers in East London live in areas characterised by high levels of deprivation (supplemental table 1).”*  We have also clarified our outcome measure (CV[R])  ***Outcome measures***  *“The primary endpoint for the multi-state model employed in this study was a major cardiovascular(36) or renal event (CVR). These are among the leading causes of death globally, and a common consequence of the cardiometabolic conditions in our ICM-MM definition.(37) CVR was defined as the earliest occurrence in the electronic healthcare record data of, or where the underlying cause of death in ONS mortality data(38) was due to:* *atrial fibrillation and flutter, heart failure, coronary heart disease, peripheral vascular disease, cerebrovascular disease (TIA/stroke), and end stage renal disease.”* |
| Were population stratifications being considered when producing PRSicm-mm? | We agree this was an important omission: following your comment and comments from other reviewers, we have heavily revised the section on genetic risk, and clarified the steps taken to address population stratification in the development of the ICMM PRS and in the G&H cohort:  *“The model was adjusted for self-reported gender and ten genetic principal components* *to account for population stratification.”*  And  *“To account for population stratification in G&H we regressed out the first 20 genetic principal components”* |
| What is the accuracy of the outcome measures (depression, hypertension, or others)?  Any washout period for internalizing disorders? | Thank you for highlighting this issue: following your suggestion and the suggestion from reviewer 2 we have respecified our cohort to include a 6 month washout period for all conditions, and updated all figures and tables accordingly.  *“Start of follow-up was defined as the cohort entry date, plus a six-month lag (or ‘washout’) period, to exclude prevalent or pre-existing conditions from the incidence risk set.(34, 35) “*  *“Eligible participants were age ≥16 years old, with ≥1 record in G&H primary care data, no record of any internalising or cardiometabolic conditions before start of follow-up, and total follow-up time >0 days. “*  We have also revised the methods section to clarify that these conditions are commonly diagnosed and managed in primary care  **Multimorbidity definition**  *“All of these conditions are commonly diagnosed and managed in primary care settings in the UK - for further information see supplemental text”* |
| Though the study focused on mental-physical multimorbidity, to what extend developing other relevant diseases/disorders, for instance neurobiological conditions ( healthy-> internalising-> dementia-> cardiometabolic) would affect the assumptions of pre-specified the multistate and how the other co-occurring diseases would influence the results? or how other pre-existing conditions, such as cancers would change the order of multimorbidity or death? | Thank you for highlighting these approaches to us: we have updated the article throughout to highlight the study specific operationalisation of multimorbidity and study endpoint, as well as the important contribution made to these by our PPI group.  Following your recommendation we have revised our limitations section further, and included some specific recommendations for future research, referencing recent review articles on the topic of multimorbidity in dementia and cancer.  *“In this study we have investigated a specific form of internalising and cardiometabolic multimorbidity, and downstream health events that are common consequences of this cluster of conditions. Future work should focus on alternate definitions of multimorbidity, and examine the impact of other conditions such as dementia and cancers on multimorbidity trajectories.(78, 79)* |
| Sociodemographic information captured sex or gender? If biological or sex at birth, then gender terminology should be replaced by females and males. | We have clarified the demographic variables section. Responses were taken from self- report questionnaire, and we refer to gender throughout.  *“Participants’ self-reported gender, and ethnicity (Bangladeshi or Pakistani) were taken from the G&H demographic questionnaire. Participants responding ‘other’ to the ethnicity item were excluded from this analysis.”* |
| Assumption of the multistate model used the baseline (static)information to predict the risk of the studied outcomes, which is not consistent with the dynamic nature of several studied risk factors (income, smoking status, the interactions between genetics and behaviors). How did the analyses consider these impacts? | We have clarified this in our limitations section. At the time of cohort specification, we were able to access IMD and smoking status at baseline – which does not necessarily reflect the dynamic nature of these events (people can and do move to areas of higher or lower deprivation, and do start / stop smoking).  *“With the notable exception of smoking(70) many of these factors, such as alcohol consumption(71) and exercise,(72) are poorly captured in EHR data.* *We were able to adjust for smoking behaviours at study baseline, but we note that some ‘never’ smokers may have started smoking after study baseline, and ‘ever’ smokers may have stopped smoking: cessation is likely to reduce or delay onset of cardiometabolic multimorbidity.(60)*  *We adjusted our models for deprivation using the index of multiple deprivation (IMD), which is a commonly used metric to assess area-level environmental risk exposures in England, but we note this is subject to the ecological fallacy.(73) Additionally, as IMD was ascertained at study baseline, any changes in living circumstances (and their impact on health trajectories over the course of follow-up) are not reflected in our results”* |
| Another major point is the multimorbidity is more closely tied to stressors (bio-psychosocial attributes) and behavioral factors (exercise, drinking, etc.). What factors are available in the database? How much change can be explored when these factors are being considered? | We have expanded our limitations section on EHR data to clarify that factors such as diet / exercise and alcohol are not well captured in EHR data and are therefore not available for most UK EHR studies  *“One key limitation is the lack of information on individual-level risk factors associated with cardiometabolic and internalising conditions. With the notable exception of smoking(70) many of these factors, such as alcohol consumption(71) and exercise,(72) are poorly captured in EHR data. “* |
| Discussion mentioned several major limitations that are threats to the accuracy of the research findings. I wonder if some sensitivity analyses can be done to examine how much influence or impact would be if some factors (say population stratification for PRS) are being corrected, how much this would change the results. | We have revised our description of the ICM-MM PRS to clarify that population stratification has been addressed by the addition of 10 genetic PCs to the elastic net regression model in UK Biobank. We further accounted for any ancestry-driven effects in G&H by regressing out 20 genetic principal components after calculating the ICM-MM PRS, and prior to standardisation.  **Please see revised methods section ‘ Genetic risk’** |

| **Reviewer 4** |  |
| --- | --- |
| Reviewer #4: Thank you the authors for submitted this interesting article on an important topic, I enjoyed reading it. I have several comments, mainly relating to the methodology, population and the presentation of the results. |  |
| **Introduction** |  |
| In the first paragraph, you could perhaps add an extra sentence explaining what some of the most co-occurring long term conditions are, for those who aren't as familiar with multimorbidity research. | We have re-worked the introduction to give examples of commonly co-occurring long-term conditions:  **Introduction**:  *“Population based studies in the United Kingdom (UK) have identified patterns of frequently co-occurring long-term conditions (sometimes referred to as multimorbidity clusters), highlighting some of the most prevalent combinations, including hypertension, type 2 diabetes, dyslipidaemia, obesity, depression, and anxiety.(7)”* |
| In the second paragraph, I think there needs to be a bit more justification as to why taking the "trajectory" approach can better help understand how and why multimorbidity develops. | We have added some more justification to support trajectory-based approaches to in multimorbidity research.  *“Some research is starting to address these knowledge gaps by examining longitudinal ordering of long-term conditions or ‘trajectories’ leading to multimorbidity.*  *Trajectory based approaches can offer insights as to the most common or harmful trajectories, as well as identifying index conditions to prioritise for intervention to prevent multimorbidity. Existing studies have identified the trajectories most strongly associated with reduced life-expectancy (in a physical-mental health multimorbidity cluster comprising diabetes, psychosis, and congestive heart failure),(10) and highlighted associations between sociodemographic factors and longitudinal accrual of long-term conditions leading to multimorbidity.(11, 12) “* |
| In the final paragraph, I think you need to further justify why in particular you focused on internalising behaviour and cardiometabolic conditions. Why not focus on other conditions? | We have revised the final two paragraphs of the introduction and the PPI section in methods to highlight that this is a study specific operationalisation of multimorbidity, developed by clinicians, members of the public with lived experiences of multimorbidity, and other members of the research team  **Introduction**  *“Many operationalisations of multimorbidity have been used in existing research.(2, 4)* *Here, we use a collaborative-specific operationalisation: the lifetime co-occurrence of internalising (depression, anxiety, somatoform) and cardiometabolic conditions (hypertension, obesity, type 2 diabetes) to characterise physical-mental health multimorbidity health trajectories in a large UK sample of British Bangladeshi and Pakistani individuals and investigate, for the first time, whether a multimorbidity polygenic risk tool alongside traditional risk factors would identify those at risk. We selected this population due to the greater burden and earlier onset of several of the single conditions in this multimorbidity cluster, and to redress the lack of representation of South Asian populations in genomic research.(21)*  *In this study, we focus on a cluster of physical and mental health multimorbidity* *comprising internalising (as opposed to externalising)(22) mental-health conditions and cardiometabolic conditions: one of the most common types of physical and mental health multimorbidity in older age.(23) This constellation of conditions, or similar, are commonly observed in clustering studies,(1, 24) and account for a significant cost to health systems.(25) and yet there is a lack of evidence on how to prevent, or manage them effectively in multimorbidity care models.(26, 27) In this study we used multi-source electronic health records to describe the transitions from a healthy state to the development of either an internalising or cardiometabolic condition and subsequent multimorbidity, followed by a major cardiovascular or renal event and death. We then investigated the influence of ethnicity (Bangladeshi and Pakistani), sociodemographic and genetic risk, using a novel polygenic risk score for multimorbidity, on progression through these trajectories.*  *”*  **Methods**  “PPI partners worked in collaboration with primary and secondary care doctors and other members of the research team to define the study specific multimorbidity operationalisation. They also contributed to the design of this analysis - highlighting priority areas and informing the research questions, including the focus on the impact of condition ordering on future health events related to multimorbidity. ” |
| **Methods** |  |
| In the first paragraph of the 'data sources and setting' sub-section I would like to see some more detail regarding the dataset, in particular the sampling framework. | We have separated ‘setting’ and ‘data sources’ into separate headings and added further detail on the convenience sample, noting the sample is broadly representative of the background population in East London.  *“Volunteers are recruited by bilingual health researchers from community (including mosques, markets and libraries), and health care settings (including GP surgeries and outpatient clinics). We restricted this study to participants with primary care data, meaning only individuals in East London were in the analysis sample (see ‘data sources’). The convenience sample is broadly representative of the background population in East London – with some over-representation of women, and those aged <45 years.(28) The majority of G&H volunteers in East London live in areas characterised by high levels of deprivation (supplemental table 1).*  *”* |
| In the second paragraph of the 'data sources and setting' sub-section, can you confirm that the phenotype data collection being in these London boroughs only means that only individuals from London are included in the analysis sample? It's a little confusing... | We apologise for the lack of clarity in this paragraph. We have clarified in ‘setting’ and ‘data sources’ – in G&H primary care data is available from London based practices only, secondary care data is national level (England and Wales). Because this study uses primary care data (linked to secondary care data), the sample is restricted to individuals in London. |
| in the outcome measures paragraph you clearly state that the primary endpoint is a CVE. This seems (for me) to be at odds with the rest of the paper which states the focus in on the impact of the risk scores and socioeconomic disadvantage on life course multimorbidity trajectories. | We have clarified that the primary study endpoint is CVR – this was highlighted by PPI partners as an outcome of interest given that mortality is a rare outcome in this relatively young cohort.  *“The primary endpoint for the multi-state model employed in this study was a cardiovascular or renal event (CVR),”* |
| In the demographic variables paragraph, I'm a little concerned about the representativeness of the sample, given some of the IMD levels have had to be collapsed. Perhaps something to be considered a little more in the discussion. | We have clarified in the ‘setting’ section that volunteers in East London live in areas characterised by high levels of deprivation.  We have also added supplemental table 1 to show IMD levels for the whole east London sample, vs this study sample. [IMD is taken from the primary care data]  Please see supplemental table 1:  “A comparison of sociodemographic characteristics of people included in the analytic sample with the Genes & Health sample in East London” |
| in the genetic risk paragraph I think you've missed a reference out by accident. Also what is PLINK 2.0? These paragraphs are full or acronyms and very difficult to read. I would try to put this is plainer language as relatively few readers of this journal will understand it as it stands. | We apologise for missing this reference – the original preprint is now *in press* with Human Molecular Genetics, and we have updated this reference accordingly  We have also revised this section thoroughly to make it clearer and easier to interpret.  **Excerpt from methods: ‘Genetic risk’**  *“In this study we used a novel PRS developed for ICM-MM called ‘ICM-MM_PRS_’, which we have described in full previously.(42) Briefly, the score was constructed in three steps: Firstly, we identified GWAS studies for each of the individual ICM-MM conditions, selecting those that did not include the UK Biobank study,(43) or made available summary statistics excluding UK Biobank participants. Secondly, we used a polygenic prediction method called ‘PRS-CS’(44) to create separate PRSs for each condition (referred to as ‘PRS_TRAIT_’ ) using UK Biobank data. Finally, we used elastic net regression, implemented in the R package ‘PRSmix’,(45) to find the optimal combination and weighting of the seven PRS_TRAIT_ scores for predicting ICM-MM in UK Biobank participants. The model was adjusted for self-reported gender and ten genetic principal components to account for population stratification.”* |
| **Statistical Analysis** |  |
| The schematic of the state transition doesn't show the ability for an individual to remain in the same state during a model cycle - can the authors confirm that the model was set up to account for this? | We can confirm that the model allows individuals to remain in a given state until they are censored at study end. |
| What software did you build your semi-markov model in? | We have updated the ‘statistical analysis’ section in methods to clarify:    *“*The multi-state model was made using the ‘fmsm’ command in the flexsurv package.(50)” |
| Why weren't transitions allowed from CVE to dead? I don't think CVE being an "endpoint" is enough justification for this. | We apologise for the lack of clarity in our definition of CVR and death as study endpoints: The definition of CV[R] includes deaths attributable to causes closely related to the ICM-MM conditions. CV[R] is the primary outcome of interest and ‘death’ in this study is then a competing risk state, comprising all other causes of death.  We have clarified this in ‘outcome measures’  *“All other underlying causes of mortality were grouped together as a competing risk state: ‘non-CVR death’ in the multi-state model”*  We have also clarified Fig1 and accompanying caption to clarify Death is all other non-CVR causes |
| I would like to see some (perhaps not all!) of the survival models plotted against the observed survival in the appendices, as well as the tables with the AIC values for different numbers of knots and the residual plots in the appendices. These are key bits of information that are missing. | We have added a modelling supplement containing plots for the knot selection process, and for observed vs modelled survival for all transitions.  **Methods**  *“and comparison of modelled vs observed survival plots for each transition (see modelling supplemental for details)”* |
| Can you further justify the 100 time points over 10 years? Why is this the model cycle length? | For simulating the state occupation probabilities, we selected ten years as the projection window to reflect the median follow up time across the study. The choice of 100 time points was based on similar analyses performed using the STATA ‘multistate’ package (and translated here into the equivalent framework in R). We concede that fewer timepoints would yield similar results – and have been computationally more efficient.  In the revised version of the analysis we have used 20 time points to improve computation time, but still with sufficient granularity for our plots 4-8. |
| **Results** |  |
| So your final sample is approximately 40% of the full Genes and Health sample. I think you need some more text about how representative this 40% is, especially as (I think?) they're all participants from London. | We have added a supplemental table to compare participants included in the final analysis with the total G&H sample in East London. This table shows that our analytic sample is very closely matched to the background sample in terms of IMD, ethnicity and gender. The analytic sample is slightly younger than the full sample.  We have also added to the ‘setting’ section some more detail on the sampling and representativeness of G&H in East London, and highlight the link to the cohort profile paper, which shows the full sample in East London (and hence the analytic sample) is broadly representative of the British Bangladeshi and British Pakistani population in East London.  **Please see supplemental table 1** |
| Why have you included the proportion of people dead in a table of baseline characteristics? | We have separated table 1 into two tables: one with characteristics at baseline and follow up years. And 2 containing health events during follow up  **Please see revised tables 1 and 2** |
| Across all age groups the probability of remaining decreased over the 10 year period" - does this pretty obvious sentence need to included? | We have combined this sentence with the next to emphasise that the pattern we describe applies to all age groups, but is especially pronounced in older age groups.  *“Across all age groups the probability of remaining healthy decreased over the ten-year period, with this decline especially pronounced in older age groups, where a high proportion of individuals were likely to experience cardiometabolic conditions”* |
| Are there any plausible mechanisms through which the multimorbid states (cardio -> internalising vs internalising -> cardio) may differ? Or is it just because of differences in age of onset? | We agree that differences in age of onset are likely to be an important factor – especially given our observation that younger cohort members are at a higher risk of internalising than cardiometabolic conditions as their index condition. Genetic risk as measured via the ICM-MM PRS seems to be expressed via higher likelihood of experiencing cardiometabolic rather than internalising as the index condition, with ICM-MM also increasing risk of internalising conditions after entering the cardiometabolic state. This may reflect higher shared genetic risk, but also the more severe expression of the cardiometabolic conditions in those with higher genetic risk. We have reflected this in the discussion. |
| You need to find a way of presenting the key results in tables rather than using multiple figures. It is extremely difficult to follow what is going on. I would need to see the results presented in a different manner before making a proper judgement. | We have created supplemental tables containing the numeric state occupation probabilities 10 years after baseline for a range of baseline ages (supplemental 4) and the contrast in state occupation probabilities for the relevant covariate patterns at 10 years (supplemental 5)  *“For details of ten-year state occupation probabilities for men and women, please see supplemental table 4.”*  *“For details of ten-year contrasts for all sociodemographic and genetic variables, please see supplemental table 5.”* |
| So the condition ordering actually might not matter that much? This needs to be fleshed out a little more and put into context with the rest of the results | We apologise if the language used was too strong: we have revised the results and discussion to emphasise that we found only limited evidence that condition ordering impacts later CV[R] risk in the discussion (please see our response to your comment on items in the discussion below) |
| What is "conventionally statistically significant"? 5%? 10%? | We have clarified that conventionally statistically significant in this case means p<0.05  Results  *“but the difference was only* *conventionally statistically significant (p<0.05)”* |
| **Discussion** |  |
| Although written well, I think the authors really need to consider whether the results from their empirical analysis back up some of the statements made in this section. For example, the results show limited evidence of any ordering effect, which I don't think is reflected in the text related to this in the discussion. | We apologise if the language used here was too strong – we have revised this section to highlight ‘only limited evidence”  *“We found only limited evidence that condition ordering impacts later CVR risk: people who developed ICM-MM along the trajectory cardiometabolic 🡪 internalising had a slightly higher probability of developing a cardiovascular event over the next ten years than the internalising 🡪 cardiometabolic trajectory, though the evidence for this was only conventionally statistically significant (p<0.05) for women who developed ICM-MM at age 40. Our findings contribute to an established literature showing that individuals with type 2 diabetes are more likely to have a CVR if they have co-existent depression.(53) The lack of longitudinal data in these prior studies meant it was not possible to see whether these associations were driven by depression prior to, or after, the diagnosis of type 2 diabetes. By characterising the ordering of conditions, we can hypothesise why there are small (but directionally consistent) differences in progression to CVR”* |
| **Limitations** |  |
| Any limitations related to the sample? | We have added a limitation about the over representation of women and people under 45 and added a recommendation for future work  *“The G&H sample in East London is broadly representative of the British Bangladeshi and British Pakistani population in East London, but over-represents slightly women and people age <45. Future work should target strategies to reweight the G&H sample to fully reflect the background population in terms of sociodemographic structure, and health.(73)”* |
| Any limitations related to the choice of empirical analysis? | Following this comment and comments from other reviewers, we have re-run our analyses using an updated cohort to allow for a 6 month washout period. |
| I'm not sure that "men are at a higher risk of CVE" is justified? | This statement is in reference to figs 3a, 3b (state occupations across varying baseline ages for men and women) and fig4: state occupation contrasts for men (vs women).  We agree that these figures were not clear, and the recommendation to add in tables summarising the numerical results makes this even clearer. We have revised the colour palette for figures 3a and b clearer, and also provided supplemental tables containing the numeric (rather than graphical) state occupation probabilities at 10 years for men and women (supplemental table 4), and the gender contrast for the reference covariate pattern (supplemental table 5) |
| "The burden of multimorbidity is high in UK South Asian population". It would good if we could compare this figure to the average in the UK and to other ethnicities. | We have added an additional reference to the introduction to highlight the higher burden of multimorbidity in UK Pakistani and Bangladeshi populations than other South Asian and European populations in the UK,  “In the UK, South Asian populations are at the highest risk of early-onset multimorbidity, leading to greater health burden, and reduced life expectancy.(13) Pakistani and Bangladeshi populations specifically are also more likely than other South Asian (and European populations) to live with multiple long-term conditions and report poorer health.(14, 15)’ |
