# Supplementary material for: Genetic and sociodemographic factors associated with trajectories of physical and mental health multimorbidity in a South Asian cohort in the UK: A multistate modelling analysis
Source: PLoS Med. 2026 Jul 9;23(7):e1004844. doi: 10.1371/journal.pmed.1004844 (PMC13349187; doi:10.1371/journal.pmed.1004844)
Supplement: Attachment — Submitted filename: 2026-06-03_changes_checklist.docx [file pmed.1004844.s010.docx]

- Title: Given how long the title has become we would suggest an alternative to improve clarity and readability ‘Genetic and sociodemographic factors associated with trajectories of physical and mental health multimorbidity in a South Asian cohort in the UK: a multistate modelling analysis’ **Genetic and sociodemographic factors associated with trajectories of physical and mental health multimorbidity in a South Asian cohort in the UK: a multistate modelling analysis**
- Abstract: We would suggest the following edits to the Abstract to improve clarity and readability. Please check for scientific accuracy: we believe the abstract is scientifically accurate with a few minor tweaks made in the latest revisions – thank you very much for the edits, which make the abstract much clearer
- Briefly define ‘internalising’ on first use. We studied Internalising (depression, anxiety, somatoform disorders) and Cardiometabolic (hypertension, obesity, type 2 diabetes, chronic kidney disease, dyslipidaemia) MultiMorbidity (ICM-MM)
- Please revise the sentence ‘10-year CVR risk was highest for transitioning to ICM-MM via cardiometabolic than internalising conditions in mid-life (age 40).’ to improve clarity. 10-year CVR risk was highest for people who developed ICM-MM via the trajectory cardiometabolic-to-internalising (versus internalising-to-cardiometabolic) in mid-life (age 40)

  Background: 
  UK South Asian populations are at high risk of physical and mental health multimorbidity, which means they live with multiple long-term conditions. The life course emergence of multimorbidity, its underlying aetiology, and consequences for future health and mortality have yet to be studied in this population. 
  Methods and Findings: 
  We studied Internalising and Cardiometabolic MultiMorbidity (ICM-MM): the lifetime occurrence of > 1 internalising mental health condition (depression, anxiety, somatoform disorders) AND > 1 cardiometabolic condition (hypertension, obesity, type 2 diabetes, chronic kidney disease, dyslipidaemia), in a longitudinal cohort of the Genes & Health project participants with linked genetic and health data from 1st April 1997 to 24th November 2024. We used multi-state models to investigate trajectories in ICM-MM and risk of major cardiovascular or renal events (CVR) or non-CVR death. We used flexible parametric models to estimate baseline hazard ratios for health state transitions, adjusting for sociodemographic factors, a polygenic risk score (PRS) for ICM-MM (ICM-MMPRS), and describe 10-year simulated health state probabilities. Over 10.2 years median follow-up of 23,554 British Bangladeshi and Pakistani participants (median baseline age 31.1 years, 12,934 [54.9%] women), 3,159
  (13.4%) developed ICM-MM; 1,522 (6.5%) CVR; and there were 103 (0.4%) non-CVR deaths. Women were less likely to remain healthy, with higher probability of developing internalising conditions and subsequent ICM-MM, but lower risk of CVR than men. Younger age was associated with higher risk of developing internalising conditions. Bangladeshi ethnicity, higher deprivation, and smoking were all associated with higher probability of ICM-MM. 10-year CVR risk was highest for transitioning to ICM-MM via cardiometabolic than internalising conditions in mid-life (age 40). Higher ICM-MMPRS was associated with higher probability of ICM-MM via cardiometabolic conditions rather than internalising conditions. Our findings are based on routinely collected electronic health records from East London, which incompletely capture individual-level and time-varying risk factors, remission or recovery, and may not reflect British Bangladeshi and British Pakistani communities across the UK. The PRS
  was derived largely from GWAS of European ancestry populations, which may limit its transferability to this cohort. 
  Conclusions: 
  The burden of multimorbidity is high in British Bangladeshi and British Pakistani populations. Young Bangladeshi women are at high risk of ICM-MM, while men are at higher risk of CVR.  Detection and intervention strategies for physical and mental health multimorbidity should be targeted early in the lifecourse, for those at highest risk. Thank you very much for the revisions to the abstract, which make the meaning clearer and easier to understand. We have incorporated all of these changes into the version in the attached full article with a few very minor changes for clarity (baseline hazards, not baseline hazard ratios, Genes & Health study – not project)

  * We would suggest some edits to the Author summary to improve clarity and readability. Please revise this sentence to make it clearer starting with a brief definition of multimorbidity and also explaining briefly what are internalising conditions. ‘A common and serious type of multimorbidity (multiple long-term conditions), involves internalising mental health conditions (depression, anxiety, somatoform disorders) and cardiometabolic conditions.’ We have reworked the first two bullet points to aid clarity and incorporate the suggested definitions
- Multimorbidity or multiple long-term conditions is defined as the co-occurrence or lifetime occurrence of two or more long term health conditions. South Asian populations living in the UK are at high risk of multimorbidity, which has a major impact on their health and quality of life.
- A common and serious type of multimorbidity, involves the co-occurrence of internalising mental health conditions (depression, anxiety, somatoform disorders) and cardiometabolic conditions. Little is known about the order in which these conditions develop, or what drives this in South Asian populations.

* Competing interests statement: Please replace the sentence ‘– none of the named authors have any competing interests’ with ‘The other authors declare that no competing interests exist.’ We have updated the COI statement online

*Data: If the data are not freely available please also include an appropriate contact (web or email address) for inquiries (again, this cannot be a study author). Our data sharing statement at the end of the article makes clear that the individual level data are available within a secure data environment to bona fide researchers - detailed instructions on how to apply for access are found at the web link provided (<https://www.genesandhealth.org/researchers/apply-for-access/>.)

*Code: Please review our guidelines at <https://eur01.safelinks.protection.outlook.com/?url=https%3A%2F%2Fjournals.plos.org%2Fplosmedicine%2Fs%2Fmaterials-software-and-code-sharing&data=05%7C02%7Cd.stow%40qmul.ac.uk%7Ccb039d4a67f74b83dad208deb2a1f20c%7C569df091b01340e386eebd9cb9e25814%7C0%7C0%7C639144605790147332%7CUnknown%7CTWFpbGZsb3d8eyJFbXB0eU1hcGkiOnRydWUsIlYiOiIwLjAuMDAwMCIsIlAiOiJXaW4zMiIsIkFOIjoiTWFpbCIsIldUIjoyfQ%3D%3D%7C0%7C%7C%7C&sdata=Jiww49b4aJglxxr3%2Bj5InQpaBh5xcZfsI5hcYXMkS4A%3D&reserved=0> and ensure that your code is shared in a way that follows best practice and facilitates reproducibility and reuse. Because Github depositions can be readily changed or deleted, we encourage you to make a permanent DOI'd copy (e.g. in Zenodo) and provide the URL.

The R code, for model fitting and associated plots, along with a minimal dataset required to reproduce the figures in the main article is available at <https://doi.org/10.5281/zenodo.20541299>
