# Supplementary material for: Genetic and sociodemographic factors associated with trajectories of physical and mental health multimorbidity in a South Asian cohort in the UK: A multistate modelling analysis
Source: PLoS Med. 2026 Jul 9;23(7):e1004844. doi: 10.1371/journal.pmed.1004844 (PMC13349187; doi:10.1371/journal.pmed.1004844)
Supplement: Peer review history

# Editor report: Decision Letter 0

**DOI:** 10.1371/journal.pmed.1004844.r001

**Authors:** Helen Howard

14 Nov 2025

Dear Dr Stow,

Thank you for submitting your manuscript entitled "The impact of polygenic risk and socioeconomic disadvantage on life course physical-mental health multimorbidity trajectories in a UK South Asian ancestry cohort" for consideration by PLOS Medicine.

Your manuscript has now been evaluated by the PLOS Medicine editorial staff, and I am writing to let you know that we would like to send your submission out for external peer review.

However, before we can send your manuscript to reviewers, we need you to complete your submission by providing the metadata that is required for full assessment. To this end, please login to Editorial Manager where you will find the paper in the 'Submissions Needing Revisions' folder on your homepage. Please click 'Revise Submission' from the Action Links and complete all additional questions in the submission questionnaire.

For clinical studies, please upload a copy of your trial study protocol as a supporting information file. The study protocol should be the version submitted for approval to the institutional review board or ethics committee, should include any amendments to the study protocol, as well as the date of their approval by the institutional review or ethics committee. Please also detail any deviations from the study protocol in the Methods section of your manuscript. The editors will consider the protocol and study conduct prior to a final decision for external review.

Please re-submit your manuscript within two working days, i.e. by Nov 18 2025 11:59PM.

Login to Editorial Manager here: https://www.editorialmanager.com/pmedicine

Once your full submission is complete, your paper will undergo a series of checks in preparation for peer review. Once your manuscript has passed all checks it will be sent out for review.

Feel free to email us at plosmedicine@plos.org if you have any queries relating to your submission.

Kind regards,

Helen Howard

PLOS Medicine

# Peer review: Decision Letter 1

**DOI:** 10.1371/journal.pmed.1004844.r002

**Authors:** Andreia Cunha

12 Jan 2026

Dear Dr Stow,

Apologies for the delay in getting back to you with a decision, which was due to challenges in securing all the necessary advice. Many thanks for submitting your manuscript "The impact of polygenic risk and socioeconomic disadvantage on life course physical-mental health multimorbidity trajectories in a UK South Asian ancestry cohort" (PMEDICINE-D-25-03970R1) to PLOS Medicine. The paper has been reviewed by subject experts and a statistician; their comments are included below and can also be accessed here: [LINK]

As you will see, all the reviewers find your study to on an important topic and of value to the field, but they have raised some important questions and concerns about it that we would need you to address in full. After discussing the paper with the editorial team and an academic editor with relevant expertise, I'm pleased to invite you to revise the paper in response to the reviewers' comments. We plan to send the revised paper to some or all of the original reviewers, and we cannot provide any guarantees at this stage regarding publication.

In addition to these revisions, you may need to complete some formatting changes, which you will receive in a follow up email. A member of our team will be in touch with a set of requests shortly. If you do not receive a separate email within a few days, please assume that checks have been completed, and no additional changes are required.

When you upload your revision, please include a point-by-point response that addresses all of the reviewer and editorial points, indicating the changes made in the manuscript and either an excerpt of the revised text or the location (eg: page and line number) where each change can be found. Please also be sure to check the general editorial comments at the end of this letter and include these in your point-by-point response. When you resubmit your paper, please include a clean version of the paper as the main article file and a version with changes tracked as a marked-up manuscript. It may also be helpful to check the guidelines for revised papers at http://journals.plos.org/plosmedicine/s/revising-your-manuscript for any that apply to your paper.

We ask that you submit your revision by Feb 12 2026 11:59PM. However, if this deadline is not feasible, please contact me by email, and we can discuss a suitable alternative.

Don't hesitate to contact me directly with any questions (acunha@plos.org).

Best regards,

Andreia

Andreia Cunha, PhD

Senior editor

PLOS Medicine

acunha@plos.org

-----------------------------------------------------------

Comments from the reviewers:

Reviewer #1: The study by Stow et al. represents a timely and novel contribution to the literature on multimorbidity, particularly at the interface of internalising mental health conditions, cardiometabolic disease, and genetic risk in under-represented British Bangladeshi and Pakistani populations. The manuscript is generally well-written, the statistical analysis is appropriate and carefully executed, and the authors have thoughtfully addressed key limitations. However, I have some comments/concerns regarding the definition of the primary endpoint and the need for additional detail and clarification in the presentation of the results and figures, which I believe would further improve the clarity and interpretability of the paper.

Major comments:

1) Regarding the definition of the primary endpoint, the authors have defined a composite "cardiovascular event (CVE)" endpoint that includes end-stage renal disease (ESRD). ESRD is conventionally regarded as a renal outcome rather than a cardiovascular one. Including ESRD within a composite labelled as purely "cardiovascular" introduces a conceptual inconsistency between the name of the endpoint and its components and may be incorrect. I recommend either: (i) removing ESRD from the primary CVE composite and analysing renal outcomes separately as a pre-specified secondary endpoint, or (ii) renaming the composite to reflect its broader scope (e.g. "major cardiovascular or renal event (CVR event)") and explicitly justifying the inclusion of ESRD as part of a combined cardio-renal endpoint.

2) Please add a supplementary table reporting, for each age category, the number of patients and the number (and percentage) of events at the end of follow-up. This should correspond to the number (percentage) of events shown in Figure 3a and Figure 3b.

3) Please add a supplementary table reporting the frequency of each internalising mental health condition and each cardiometabolic condition in the study population. I also suggest adding a brief section to the Results and Discussion describing which specific conditions were most prevalent within each group, and commenting on how this pattern of prevalence may influence the interpretation of the multimorbidity trajectories.

Minor comments:

4) In the Methods-Genetic Risk section, the references to the previous study/studies that described the methods used to derive the novel PRS for ICM-MM are missing.

5) Some abbreviations are spelled out in full and then repeated inconsistently in different sections of the manuscript. I suggest revising the entire manuscript to ensure that all abbreviations are introduced and used consistently, in line with the journal's guidelines.

6) In Table 1, there appears to be an error in the column heading for the Pakistani female group (it is incorrectly labelled as male). In addition, please report all numerical values with a consistent number of decimal places (e.g. 1 or 2 decimals, according to the journal's guidelines). For example, some numbers for the ICMM PGS and follow-up duration are currently reported with up to 5 decimal places.

7) For Figure 1, I recommend adding the definitions of the internalising mental health conditions and cardiometabolic conditions to the figure legend, to help readers fully understand how the model works and what the target multimorbidity cluster comprises.

8) Figure 3a and Figure 3b are very important and informative. However, with seven classes (six blue coloured states), it is difficult for readers to distinguish between the "multimorbid (INT→CMD)", "multimorbid (CMD→INT)", and "cardiovascular event" states. I highly recommend using different colours/shades to increase visual separation between these states.

9) For Figure 6: I suggest adding the full term "Index of Multiple Deprivation (IMD)" in the Figure 6 legend, and using "Index of Multiple Deprivation contrast" instead of "IMD contrast" in the figure title, to aid reader understanding, particularly for those unfamiliar with the abbreviation.

Sina Kazemian, MD

Dec/2/2025

Reviewer #3: The manuscript aimed to examine life course of mental-physical multimorbidity among UK South Asian population between 1997 to 2024. Multi-state models were applied to explore trajectories of internalising and cardiometabolic multimorbidity and risk of cardiovascular event or death. The study observed that 14% of 25641 Bangladeshi and Pakistani participants developed the multimorbidity and 7% with cardiovascular events and 0.5% died. Women, younger age, people with higher deprivation, and people who smoked tend to report multimorbidity. The study focused on a prevalent and important health issue-co-existence of mental-physical multimorbidity. At the same time, sociocultural issues (racialized populations) are intertwined with the disease outcome.

There are a few remaining concerns to be addressed in the manuscript.

Major issues:

The Introduction explained why decided to examine the disease outcome among UK South Asian population, but from the readers of the journal perspectives, a global literature review on the life course of multimorbidity is necessary. What has been discovered? What are the remaining knowledge gaps? Challenges and opportunities to address the necessity of life course of multimorbidity? Why are UK South Asians being studied in this context? And others to elaborate on the contextual information around the study topic.

Methods:

What is the coverage of G&H for UK South Asians? Similarly, what is the coverage of outcome measures for the studied populations?

Were population stratifications being considered when producing PRSicm-mm?

What is the accuracy of the outcome measures (depression, hypertension, or others)? Any washout period for internalizing disorders?

Though the study focused on mental-physical multimorbidity, to what extend developing other relevant diseases/disorders, for instance neurobiological conditions ( healthy-> internalising-> dementia-> cardiometabolic) would affect the assumptions of pre-specified the multistate and how the other co-occurring diseases would influence the results? or how other pre-existing conditions, such as cancers would change the order of multimorbidity or death?

Sociodemographic information captured sex or gender? If biological or sex at birth, then gender terminology should be replaced by females and males.

Assumption of the multistate model used the baseline (static)information to predict the risk of the studied outcomes, which is not consistent with the dynamic nature of several studied risk factors (income, smoking status, the interactions between genetics and behaviors). How did the analyses consider these impacts?

Another major point is the multimorbidity is more closely tied to stressors (bio-psychosocial attributes) and behavioral factors (exercise, drinking, etc.). What factors are available in the database? How much change can be explored when these factors are being considered?

Discussion mentioned several major limitations that are threats to the accuracy of the research findings. I wonder if some sensitivity analyses can be done to examine how much influence or impact would be if some factors (say population stratification for PRS) are being corrected, how much this would change the results.

Reviewer #4: Thank you the authors for submitted this interesting article on an important topic, I enjoyed reading it. I have several comments, mainly relating to the methodology, population and the presentation of the results.

Introduction

In the first paragraph, you could perhaps add an extra sentence explaining what some of the most co-occurring long term conditions are, for those who aren't as familiar with multimorbidity research.

In the second paragraph, I think there needs to be a bit more justification as to why taking the "trajectory" approach can better help understand how and why mulitmorbidity develops.

In the final paragraph, I think you need to further justify why in particular you focused on internalising behaviour and cardiometabolic conditions. Why not focus on other conditions?

Methods

In the first paragraph of the 'data sources and setting' sub-section I would like to see some more detail regarding the dataset, in particular the sampling framework.

In the second paragraph of the 'data sources and setting' sub-section, can you confirm that the phenotype data collection being in these London boroughs only means that only individuals from London are included in the analysis sample? It's a little confusing...

in the outcome measures paragraph you clearly state that the primary endpoint is a CVE. This seems (for me) to be at odds with the rest of the paper which states the focus in on the impact of the risk scores and socioeconomic disadvantage on life course multimorbidity trajectories.

In the demographic variables paragraph, I'm a little concerned about the representativeness of the sample, given some of the IMD levels have had to be collapsed. Perhaps something to be considered a little more in the discussion.

in the genetic risk paragraph I think you've missed a reference out by accident. Also what is PLINK 2.0? These paragraphs are full or acronyms and very difficult to read. I would try to put this is plainer language as relatively few readers of this journal will understand it as it stands.

Statistical Analysis

The schematic of the state transition doesn't show the ability for an individual to remain in the same state during a model cycle - can the authors confirm that the model was set up to account for this?

What software did you build your semi-markov model in?

Why weren't transitions allowed from CVE to dead? I don't think CVE being an "endpoint" is enough justification for this.

I would like to see some (perhaps not all!) of the survival models plotted against the observed survival in the appendices, as well as the tables with the AIC values for different numbers of knots and the residual plots in the appendices. These are key bits of information that are missing.

Can you further justify the 100 time points over 10 years? Why is this the model cycle length?

Results

So your final sample is approximately 40% of the full Genes and Health sample. I think you need some more text about how representative this 40% is, especially as (I think?) they're all participants from London.

Table 1

Why have you included the proportion of people dead in a table of baseline characteristics?

Multistate models

"Across all age groups the probability of remaining decreased over the 10 year period" - does this pretty obvious sentence need to included?

Are there any plausible mechanisms through which the multimorbid states (cardio -> internalising vs internalising -> cardio) may differ? Or is it just because of differences in age of onset?

You need to find a way of presenting the key results in tables rather than using multiple figures. It is extremely difficult to follow what is going on. I would need to see the results presented in a different manner before making a proper judgement.

Condition ordering and CVE risk

So the condition ordering actually might not matter that much? This needs to be fleshed out a little more and put into context with the rest of the results

What is "conventionally statistically significant"? 5%? 10%?

Discussion

Although written well, I think the authors really need to consider whether the results from their empirical analysis back up some of the statements made in this section. For example, the results show limited evidence of any ordering effect, which I don't think is reflected in the text related to this in the discussion.

Limitations

Any limitations related to the sample?

Any limitations related to the choice of empirical analysis?

I'm not sure that "men are at a higher risk of CVE" is justified?

"The burden of multimorbidity is high in UK South Asian population". It would good if we could compare this figure to the average in the UK and to other ethnicities.

Any attachments provided with reviews can be seen via the following link: [LINK]

--------------------------------------------------------- ---

General editorial requests:

(Note: not all will apply to your paper, but please check each item carefully)

* We ask every co-author listed on the manuscript to fill in a contributing author statement, making sure to declare all competing interests. If any of the co-authors have not filled in the statement, we will remind them to do so when the paper is revised. If all statements are not completed in a timely fashion this could hold up the re-review process. If new competing interests are declared later in the revision process, this may also hold up the submission. Should there be a problem getting one of your co-authors to fill in a statement we will be in contact. Please do not add or remove authors without first discussing this with the handling editor. You can see our competing interests policy here: http://journals.plos.org/plosmedicine/s/competing-interests.

* Please upload any figures associated with your paper as individual TIF or EPS files with 300dpi resolution at resubmission; please read our figure guidelines for more information on our requirements: http://journals.plos.org/plosmedicine/s/figures. While revising your submission, we strongly recommend that you use PLOS's NAAS tool (https://ngplosjournals.pagemajik.ai/artanalysis) to test your figure files. NAAS can convert your figure files to the TIFF file type and meet basic requirements (such as print size, resolution), or provide you with a report on issues that do not meet our requirements and that NAAS cannot fix.

After uploading your figures to PLOS's NAAS tool - https://ngplosjournals.pagemajik.ai/artanalysis, NAAS will process the files provided and display the results in the "Uploaded Files" section of the page as the processing is complete.

If the uploaded figures meet our requirements (or NAAS is able to fix the files to meet our requirements), the figure will be marked as "fixed" above. If NAAS is unable to fix the files, a red "failed" label will appear above.

When NAAS has confirmed that the figure files meet our requirements, please download the file via the download option, and include these NAAS processed figure files when submitting your revised manuscript.

* Please ensure that the paper adheres to the PLOS Data Availability Policy (see http://journals.plos.org/plosmedicine/s/data-availability), which requires that all data underlying the study's findings be provided in a repository or as Supporting Information. For data residing with a third party, authors are required to provide instructions with contact information (web or email address) for obtaining the data. Please note that a study author cannot be the contact person for the data. PLOS journals do not allow statements supported by "data not shown" or "unpublished results." For such statements, authors must provide supporting data or cite public sources that include it.

* We expect all researchers with submissions to PLOS in which author-generated code underpins the findings in the manuscript to make all author-generated code available without restrictions upon publication of the work. In cases where code is central to the manuscript, we may require the code to be made available as a condition of publication. Authors are responsible for ensuring that the code is reusable and well documented. Please make any custom code available, either as part of your data deposition or as a supplementary file. Please add a sentence to your data availability statement regarding any code used in the study, e.g. "The code used in the analysis is available from Github [URL] and archived in Zenodo [DOI link]" Please review our guidelines at https://journals.plos.org/plosmedicine/s/materials-software-and-code-sharing and ensure that your code is shared in a way that follows best practice and facilitates reproducibility and reuse. Because Github depositions can be readily changed or deleted, we encourage you to make a permanent DOI'd copy (e.g. in Zenodo) and provide the URL.

FORMATTING - GENERAL

* Abstract: Please structure your abstract using the PLOS Medicine headings (Background, Methods and Findings, Conclusions). Please combine the Methods and Findings sections into one section.

* At this stage, we ask that you include a short, non-technical Author Summary of your research to make findings accessible to a wide audience that includes both scientists and non-scientists. The Author Summary should immediately follow the Abstract in your revised manuscript. This text is subject to editorial change and should be distinct from the scientific abstract. Ideally each sub-heading should contain 2-3 single sentence, concise bullet points containing the most salient points from your study. In the final bullet point of 'What Do These Findings Mean?', please include the main limitations of the study in non-technical language. Please see our author guidelines for more information: https://journals.plos.org/plosmedicine/s/revising-your-manuscript#loc-author-summary.

* Please express the main results with 95% CIs as well as p values. When reporting p values please report as p<0.001 and where higher as the exact p value p=0.002, for example. Throughout, suggest reporting statistical information as follows to improve clarity for the reader "22% (95% CI [13%,28%]; p</=)". Please be sure to define all numerical values at first use.

* Please include page numbers and line numbers in the manuscript file. Use continuous line numbers (do not restart the numbering on each page).

* Please cite the reference numbers in square brackets. Citations should precede punctuation.

FIGURES AND TABLES

* Please provide titles and legends for all figures and tables (including those in Supporting Information files).

* Please define all abbreviations used in each figure/table (including those in Supporting Information files).

* Please consider avoiding the use of red and green in order to make your figure more accessible to those with color blindness.

SUPPLEMENTARY MATERIAL

* Please note that supplementary material will be posted as supplied by the authors. Therefore, please amend it according to the relevant comments outlined here.

* Please cite your Supporting Information as outlined here: https://journals.plos.org/plosmedicine/s/supporting-information

REFERENCES

* PLOS uses the numbered citation (citation-sequence) method and first six authors, et al.

* Please ensure that journal name abbreviations match those found in the National Center for Biotechnology Information (NCBI) databases (http://www.ncbi.nlm.nih.gov/nlmcatalog/journals), and are appropriately formatted and capitalised.

* Where website addresses are cited, please include the complete URL and specify the date of access (e.g. [accessed: 12/06/2023]).

* Please also see https://journals.plos.org/plosmedicine/s/submission-guidelines#loc-references for further details on reference formatting.

OBSERVATIONAL STUDIES

* Abstract: Please include the study design, population and setting, number of participants, years during which the study took place (enrollment and follow up), length of follow up, and main outcome measures.

* Please ensure that the study is reported according to the STROBE (or appropriate STOBE extension) guideline (available from: https://www.equator-network.org/reporting-guidelines/strobe) and include the completed STROBE (or STROBE extension) checklist as Supporting Information. Please add the following statement, or similar, to the Methods: "This study is reported as per the Strengthening the Reporting of Observational Studies in Epidemiology (STROBE) guideline (S1 Checklist)." When completing the checklist, please use section and paragraph numbers, rather than page numbers.

* POPULATION HEALTH/REGISTRY STUDIES: Please ensure that the study is reported according to the RECORD guideline (available from https://www.record-statement.org) and include the completed checklist as Supporting Information. Please add the following statement, or similar, to the Methods: "This study is reported as per the Reporting of Studies Conducted using Observational Routinely-Collected Data (RECORD) guideline (S1 Checklist)." When completing the checklist, please use section and paragraph numbers, rather than page numbers.

* POPULATION HEALTH ESTIMATES STUDIES: Please ensure that the study is reported according to the GATHER statement (available from https://www.equator-network.org/reporting-guidelines/gather-statement) and include the completed checklist as Supporting Information. Please add the following statement, or similar, to the Methods: "This study is reported as per the Guidelines for Accurate and Transparent Health Estimates Reporting (GATHER) statement (S1 Checklist)." When completing the checklist, please use section and paragraph numbers, rather than page numbers.

* MEDIATION ANALYSES: We recommend that the study is reported according to the AGReMA statement (https://agrema-statement.org/#:~:text=AGReMA%20is%20an%20evidence%2D%20and,randomised%20trials%20and%20observational%20studies) and include the completed checklist as Supporting Information. Please add the following statement, or similar, to the Methods: "This study is reported as per the Guideline for Reporting Mediation Analyses (AGReMA) statement (S1 Checklist)." When completing the checklist, please use section and paragraph numbers, rather than page numbers.

* For all observational studies, in the manuscript text, please indicate: (1) the specific hypotheses you intended to test, (2) the analytical methods by which you planned to test them, (3) the analyses you actually performed, and (4) when reported analyses differ from those that were planned, transparent explanations for differences that affect the reliability of the study's results. If a reported analysis was performed based on an interesting but unanticipated pattern in the data, please be clear that the analysis was data driven.

* Please state in the Methods section whether the study had a prospective protocol or analysis plan. If a prospective analysis plan (from your funding proposal, IRB or other ethics committee submission, study protocol, or other planning document written before analyzing the data) was used in designing the study, please include the relevant document(s) with your revised manuscript as a Supporting Information file to be published alongside your study and cite it in the Methods section. A legend for this file should be included at the end of your manuscript. If no such document exists, please make sure that the Methods section transparently describes when analyses were planned, and when/why any data-driven changes to analyses took place. Changes in the analysis, including those made in response to peer review comments, should be identified as such in the Methods section of the paper, with rationale.

MODELLING STUDIES

The following list is derived from Geoffrey P Garnett, Simon Cousens, Timothy B Hallett, Richard Steketee, Neff Walker. Mathematical models in the evaluation of health programmes. (2011) Lancet DOI:10.1016/S0140-6736(10)61505-X:

* If pertinent, please provide a diagram that shows the model structure, including how the natural history of the disease is represented, the process and determinants of disease acquisition, and how the putative intervention could affect the system.

* Please provide a complete list of model parameters, including clear and precise descriptions of the meaning of each parameter, together with the values or ranges for each, with justification or the primary source cited and important caveats about the use of these values noted.

* Please provide a clear statement about how the model was fitted to the data, including goodness-of-fit measure, the numerical algorithm used, which parameter varied, constraints imposed on parameter values, and starting conditions.

* For uncertainty analyses, please state the sources of uncertainties quantified and not quantified [can include parameter, data, and model structure].

* Please provide sensitivity analyses to identify which parameter values are most important in the model. Uncertainty estimates seek to derive a range of credible results on the basis of an exploration of the range of reasonable parameter values. The choice of method should be presented and justified.

* Please discuss the scientific rationale for the choice of model structure and identify points where this choice could influence conclusions drawn. Please also describe the strength of the scientific basis underlying the key model assumptions.

* For studies that develop a prediction model or evaluate its performance, please ensure that the study is reported according to the TRIPOD statement (https://www.equator-network.org/reporting-guidelines/tripod-statement) and include the completed checklist as Supporting Information. Please add the following statement, or similar, to the Methods: "This study is reported as per the Transparent Reporting of a Multivariable Prediction Model for Individual Prognosis Or Diagnosis (TRIPOD) statement (S1 Checklist)." For studies using machine learning, please use the TRIPOD-AI checklist. When completing the checklist, please use section and paragraph numbers, rather than page numbers.

Attachment Submitted filename: paper review PLOS medicine.docx

# Author comment: Author response to Decision Letter 2

**DOI:** 10.1371/journal.pmed.1004844.r003

27 Mar 2026

Attachment Submitted filename: 2026-03-27_REVIEW_RESPONSE.docx

# Peer review: Decision Letter 2

**DOI:** 10.1371/journal.pmed.1004844.r004

**Authors:** Andreia Cunha

22 Apr 2026

Dear Dr. Stow,

Sincere apologies for the delay in getting back to you with a decision, which was due to challenges in securing all the necessary advice. Thank you very much for re-submitting your manuscript "The impact of genetic and sociodemographic factors on life course trajectories of physical-mental health multimorbidity in a UK South Asian cohort" (PMEDICINE-D-25-03970R2) for review by PLOS Medicine.

I have discussed the paper with my colleagues and the academic editor, who looked at your response to Reviewer 3, and it was also seen again by the other original reviewers. I am pleased to say that provided the remaining editorial and production issues are dealt with we are planning to accept the paper for publication in the journal.

The remaining issues that need to be addressed are listed at the end of this email. Any accompanying reviewer attachments can be seen via the link below. Please take these into account before resubmitting your manuscript:

[LINK]

***Please note while forming your response, if your article is accepted, you may have the opportunity to make the peer review history publicly available. The record will include editor decision letters (with reviews) and your responses to reviewer comments. If eligible, we will contact you to opt in or out.***

In revising the manuscript for further consideration here, please ensure you address the specific points made by each reviewer and the editors. In your rebuttal letter you should indicate your response to the reviewers' and editors' comments and the changes you have made in the manuscript. Please submit a clean version of the paper as the main article file. A version with changes marked must also be uploaded as a marked up manuscript file.

Please also check the guidelines for revised papers at http://journals.plos.org/plosmedicine/s/revising-your-manuscript for any that apply to your paper. If you haven't already, we ask that you provide a short, non-technical Author Summary of your research to make findings accessible to a wide audience that includes both scientists and non-scientists. The Author Summary should immediately follow the Abstract in your revised manuscript. This text is subject to editorial change and should be distinct from the scientific abstract.

In addition to these revisions, you may need to complete some formatting changes, which you will receive in a follow up email. A member of our team will be in touch with a set of requests shortly. If you do not receive a separate email within a few days, please assume that checks have been completed, and no additional changes are required.

We expect to receive your revised manuscript within 1 week. Please email us (plosmedicine@plos.org) if you have any questions or concerns.

We ask every co-author listed on the manuscript to fill in a contributing author statement. If any of the co-authors have not filled in the statement, we will remind them to do so when the paper is revised. If all statements are not completed in a timely fashion this could hold up the re-review process. Should there be a problem getting one of your co-authors to fill in a statement we will be in contact. YOU MUST NOT ADD OR REMOVE AUTHORS UNLESS YOU HAVE ALERTED THE EDITOR HANDLING THE MANUSCRIPT TO THE CHANGE AND THEY SPECIFICALLY HAVE AGREED TO IT.

Please ensure that the paper adheres to the PLOS Data Availability Policy (see http://journals.plos.org/plosmedicine/s/data-availability), which requires that all data underlying the study's findings be provided in a repository or as Supporting Information. For data residing with a third party, authors are required to provide instructions with contact information for obtaining the data. PLOS journals do not allow statements supported by "data not shown" or "unpublished results." For such statements, authors must provide supporting data or cite public sources that include it.

To enhance the reproducibility of your results, we recommend that you deposit your laboratory protocols in protocols.io, where a protocol can be assigned its own identifier (DOI) such that it can be cited independently in the future. Additionally, PLOS ONE offers an option to publish peer-reviewed clinical study protocols. Read more information on sharing protocols at https://plos.org/protocols?utm_medium=editorial-email&utm_source=authorletters&utm_campaign=protocols

Please review your reference list to ensure that it is complete and correct. If you have cited papers that have been retracted, please include the rationale for doing so in the manuscript text, or remove these references and replace them with relevant current references. Any changes to the reference list should be mentioned in the rebuttal letter that accompanies your revised manuscript.

Please note, when your manuscript is accepted, an uncorrected proof of your manuscript will be published online ahead of the final version, unless you've already opted out via the online submission form. If, for any reason, you do not want an earlier version of your manuscript published online or are unsure if you have already indicated as such, please let the journal staff know immediately at plosmedicine@plos.org.

If you have any questions in the meantime, please contact me or the journal staff on plosmedicine@plos.org.

We look forward to receiving the revised manuscript by Apr 29 2026 11:59PM.

Sincerely,

Andreia Cunha, PhD

Senior Editor

PLOS Medicine

plosmedicine.org

------------------------------------------------------------

Requests from Editors:

GENERAL EDITORIAL REQUESTS

* At this stage, we ask that you include a short, non-technical Author Summary of your research to make findings accessible to a wide audience that includes both scientists and non-scientists. The Author Summary should immediately follow the Abstract in your revised manuscript. This text is subject to editorial change and should be distinct from the scientific abstract. Ideally each sub-heading should contain 2-3 single sentence, concise bullet points containing the most salient points from your study. In the final bullet point of ‘What Do These Findings Mean?’ Please include the main limitations of the study in non-technical language.

Please see our author guidelines for more information: https://journals.plos.org/plosmedicine/s/revising-your-manuscript#loc-author-summary.

* Please revise your title to comply with PLOS Medicine's style. Your title must be nondeclarative and not a question. It should begin with main concept if possible. "Effect of" should be used only if causality can be inferred, i.e., for an RCT. Please remove the word impact. Please also place the study design ("A randomized controlled trial," "A retrospective study," "A modelling study," etc.) in the subtitle (ie, after a colon).

* Please confirm that your abstract complies with our requirements, including format (three sections: Background, Methods and Findings, and Conclusions) and providing all the information relevant to this study type https://journals.plos.org/plosmedicine/s/submission-guidelines#loc-abstract

* Please ensure that the Introduction ends with a clear description of the study question or hypothesis.

* Please ensure that all abbreviations are defined at first use throughout the text.

* Please confirm that all numbers presented in the abstract are present and identical to numbers presented in the main manuscript text.

GENERAL

* Since the manuscript focuses on British Pakistani and British Bangladeshi ethnicity please consider using more specific language in the title, abstract and manuscript instead of referring to a South Asian cohort.

* Please review your text for claims of novelty or primacy (e.g. 'for the first time') and remove this language. In addition, please check that any use of statistical terms (such as trend or significant) are supported by the data, and if not please remove them.

* Please remove the 'conclusions' subheading from the discussion. Please also remove any other subheadings from the discussion.

* Statistical reporting: Please revise throughout the manuscript, including tables and figures.

- Please report statistical information as follows to improve clarity for the reader 22% (95% CI [13,28]; p</=).

- Please separate upper and lower bounds with commas instead of hyphens as the latter can be confused with reporting of negative values.

- Please repeat statistical definitions (HR, CI etc.) for each set of parentheses.

* In the last sentence of the Abstract Methods and Findings section, please describe the main limitation(s) of the study's methodology.

* In the author summary, in the final bullet point of 'What Do These Findings Mean?', please include the main limitations of the study in non-technical language.

FUNDING STATEMENT

* The funding statement should include: specific grant numbers, initials of authors who received each award, URLs to sponsors’ websites. Also, please state whether any sponsors or funders (other than the named authors) played any role in study design, data collection and analysis, the decision to publish, or preparation of the manuscript. If they had no role in the research, include this sentence: “The funders had no role in study design, data collection and analysis, decision to publish, or preparation of the manuscript.”

* We would suggest moving the following text from the funding statement to the acknowledgements section:

'We thank Social Action for Health, Centre of The Cell, members of our Community Advisory Group, and staff who have recruited and collected data from volunteers. We thank the NIHR National Biosample Centre (UK Biocentre), the Social Genetic & 684 Developmental Psychiatry Centre (King's College London), Wellcome Sanger Institute, and Broad Institute for sample processing, genotyping, sequencing and variant annotation.

COMPETING INTERESTS STATEMENT

* All authors must declare their relevant competing interests per the PLOS policy, which can be seen here: https://journals.plos.org/plosmedicine/s/competing-interests For authors with ties to industry, please indicate whether any of the interests has a financial stake in the results of the current study.

DATA AVAILABILITY

* PLOS defines the “minimal data set” to consist of the data set used to reach the conclusions drawn in the manuscript with related metadata and methods, and any additional data required to replicate the reported study findings in their entirety. Authors do not need to submit their entire data set, or the raw data collected during an investigation. Please submit the following data:

The values behind the means, standard deviations and other measures reported;

The values used to build graphs;

The points extracted from images for analysis.

* The Data Availability Statement (DAS) requires revision. For each data source used in your study:

a) If the data are freely or publicly available, note this and state the location of the data: within the paper, in Supporting Information files, or in a public repository (include the DOI or accession number).

b) If the data are owned by a third party but freely available upon request, please note this and state the owner of the data set and contact information for data requests (web or email address). Note that a study author cannot be the contact person for the data.

c) If the data are not freely available, please describe briefly the ethical, legal, or contractual restriction that prevents you from sharing it. Please also include an appropriate contact (web or email address) for inquiries (again, this cannot be a study author).

* For studies in which a novel model is central to the manuscript's findings, as is the case here, authors are responsible for providing the source code needed to replicate the study's findings in a repository (such as GitHub, SourceForge or Bitbucket) or a cloud computing service (such as Code Ocean). Protection of authors’ intellectual property will not be cause for exception. Please explain in the manuscript’s Data Availability Statement how readers can access the shared code.

* Please include the statement on code availability in the data availability statement.

FIGURES

* Please provide titles and legends for all figures and tables (including those in Supporting Information files). Please define all acronyms used in each figure or table in its corresponding legend.

* Please ensure that where relevant figures include 95% CIs.

* Please consider avoiding the use of red and green in order to make your figure more accessible

OBSERVATIONAL, COHORT, CROSS-SECTIONAL, AND CASE CONTROL STUDIES

* Please ensure that the study is reported according to the STROBE guideline, and include the completed STROBE checklist as Supporting Information. Please add the following statement, or similar, to the Methods: ""This study is reported as per the Strengthening the Reporting of Observational Studies in Epidemiology (STROBE) guideline (S1 Checklist).""

The STROBE guideline can be found here: http://www.equator-network.org/reporting-guidelines/strobe/

When completing the checklist, please use section and paragraph numbers, rather than page numbers.

* Did your study have a prospective protocol or analysis plan? Please state this (either way) early in the Methods section.

a) If a prospective analysis plan (from your funding proposal, IRB or other ethics committee submission, study protocol, or other planning document written before analyzing the data) was used in designing the study, please include the relevant prospectively written document with your revised manuscript as a Supporting Information file to be published alongside your study, and cite it in the Methods section. A legend for this file should be included at the end of your manuscript.

b) If no such document exists, please make sure that the Methods section transparently describes when analyses were planned, and when/why any data-driven changes to analyses took place.

c) In either case, changes in the analysis-- including those made in response to peer review comments-- should be identified as such in the Methods section of the paper, with rationale.

* Your study is observational and therefore causality cannot be inferred. Please remove language that implies causality and refer to associations instead.

* For all observational studies, in the manuscript text, please indicate: (1) the specific hypotheses you intended to test, (2) the analytical methods by which you planned to test them, (3) the analyses you actually performed, and (4) when reported analyses differ from those that were planned, transparent explanations for differences that affect the reliability of the study's results. If a reported analysis was performed based on an interesting but unanticipated pattern in the data, please be clear that the analysis was data-driven.

MODELING STUDIES

For more details on the requests below please see Geoffrey P Garnett, Simon Cousens, Timothy B Hallett, Richard Steketee, Neff Walker. Mathematical models in the evaluation of health programmes. (2011) Lancet DOI:10.1016/S0140-6736(10)61505-X.

* Please provide a diagram that shows the model structure, including how the disease natural history is represented, the process and determinants of disease acquisition, and how the putative intervention could affect the system.

* Please provide a complete list of model parameters, including clear and precise descriptions of the meaning of each parameter, together with the values or ranges for each, with justification or the primary source cited, and important caveats about the use of these values noted.

* Please provide a clear statement about how the model was fitted to the data including where relevant goodness-of-fit measure, the numerical algorithm used, which parameter varied, constraints imposed on parameter values, and starting conditions.

* For uncertainty analyses, please state the sources of uncertainties quantified and not quantified this can include parameter, data, and model structure.

* Please provide sensitivity analyses to identify which parameter values are most important in the model. Uncertainty estimates seek to derive a range of credible results on the basis of an exploration of the range of reasonable parameter values. The choice of method should be presented and justified.

* Please discuss the scientific rationale for this choice of model structure and identify points where this choice could influence conclusions drawn. Please also describe the strength of the scientific basis underlying the key model assumptions.

Comments from Reviewers:

Reviewer #1: The authors have addressed all comments and edits perfectly during the revision, and I have no additional comments.

Reviewer #2: I thank the authors for their detailed and considered response to my comments. I am happy with their responses and feel the paper has been strengthened accordingly.

Reviewer #4: Thank you to the authors for comprehensively responding to my comments. I am happy to approve for publication.

Any attachments provided with reviews can be seen via the following link:

[LINK]

# Author comment: Author response to Decision Letter 3

**DOI:** 10.1371/journal.pmed.1004844.r005

4 Jun 2026

Attachment Submitted filename: 2026-06-03_changes_checklist.docx

# Editor report: Decision Letter 3

**DOI:** 10.1371/journal.pmed.1004844.r006

**Authors:** Andreia Cunha

5 Jun 2026

Dear Dr Stow,

On behalf of my colleagues and the Academic Editor, Dr Stefan Stender, I am pleased to inform you that we have agreed to publish your manuscript "Genetic and sociodemographic factors associated with trajectories of physical and mental health multimorbidity in a South Asian cohort in the UK: a multistate modelling analysis" (PMEDICINE-D-25-03970R3) in PLOS Medicine.

Before your manuscript can be formally accepted you will need to complete some formatting changes, which you will receive in a follow up email. Namely, the wording of the data availability statement needs rephrasing. Please be aware that it may take several days for you to receive this email; during this time no action is required by you. Once you have received these formatting requests, please note that your manuscript will not be scheduled for publication until you have made the required changes.

In the meantime, please log into Editorial Manager at http://www.editorialmanager.com/pmedicine/, click the "Update My Information" link at the top of the page, and update your user information to ensure an efficient production process.

PRESS

We frequently collaborate with press offices. If your institution or institutions have a press office, please notify them about your upcoming paper at this point, to enable them to help maximise its impact. If the press office is planning to promote your findings, we would be grateful if they could coordinate with medicinepress@plos.org. If you have not yet opted out of the early version process, we ask that you notify us immediately of any press plans so that we may do so on your behalf.

We also ask that you take this opportunity to read our Embargo Policy regarding the discussion, promotion and media coverage of work that is yet to be published by PLOS. As your manuscript is not yet published, it is bound by the conditions of our Embargo Policy. Please be aware that this policy is in place both to ensure that any press coverage of your article is fully substantiated and to provide a direct link between such coverage and the published work. For full details of our Embargo Policy, please visit http://www.plos.org/about/media-inquiries/embargo-policy/.

To enhance the reproducibility of your results, we recommend that you deposit your laboratory protocols in protocols.io, where a protocol can be assigned its own identifier (DOI) such that it can be cited independently in the future. Additionally, PLOS ONE offers an option to publish peer-reviewed clinical study protocols. Read more information on sharing protocols at https://plos.org/protocols?utm_medium=editorial-email&utm_source=authorletters&utm_campaign=protocols

Thank you again for submitting to PLOS Medicine. We look forward to publishing your paper.

Sincerely,

Andreia Cunha, PhD

Senior Editor

PLOS Medicine
